# Prognostic and Immunological Value of *CTNNB1* in Pancreatic Adenocarcinoma: A Comprehensive NGS and Multicomponent Analysis

**DOI:** 10.1155/genr/8700258

**Published:** 2026-09-09

**Authors:** ShuYan Liu, Yichen Wu, Hong Zhou, Ying Ding

**Affiliations:** ^1^ Department of Pathology, Nantong First People’s Hospital, Nantong Jiangsu, 226001, China, nt2191.com; ^2^ Department of Pharmaceutical Engineering, Nanjing University of Technology, Nanjing 210000, China, njtech.edu.cn; ^3^ Department of Pathology, The First Affiliated Hospital With Nanjing Medical University, Nanjing 210000, China, njmu.edu.cn

**Keywords:** biomarker, *CTNNB1*, immune microenvironment, immunotherapy, pancreatic cancer, prognosis

## Abstract

**Background:**

To explore the prognostic value and immunomodulatory role of *CTNNB1* in pancreatic ductal adenocarcinoma (PDAC), so as to provide evidence for the diagnosis and individualized treatment of PDAC.

**Methods:**

Next‐generation sequencing (NGS) was performed to detect 425 cancer‐associated genes in PDAC specimens and matched normal pancreatic tissues. Integrative transcriptome analysis based on TCGA, GTEx, and multiple GEO datasets was conducted to identify the differential expression of *CTNNB1* between PDAC and normal pancreatic tissues. The Human Protein Atlas (HPA, https://www.proteinatlas.org/) database was used to verify the protein expression and subcellular localization of *CTNNB1* in normal pancreatic tissues and pancreatic ductal adenocarcinoma (PDAC) tissues. Single‐cell RNA‐sequencing and spatial transcriptomic data were further supplemented to delineate its cellular expression landscape. Forest plot, Kaplan–Meier survival analysis, and ROC curve analysis were applied to evaluate the correlations between *CTNNB1* expression and prognostic and to verify its prognostic accuracy. The prognostic reliability was further validated using the independent cohort GSE57495. Immune cell infiltration and immune regulatory factors were quantified via TIMER, EPIC, MCPcounter, CIBERSORT, and xCell algorithms, and TMEscore was calculated to predict the responsiveness to immune checkpoint blockade (ICB). Pharmacogenomic datasets including GDSC1, GDSC2, PRISM, and CTRP were utilized to screen optimal candidate drugs for *CTNNB1*‐high PDAC patients.

**Results:**

*CTNNB1* was significantly overexpressed in PDAC compared with normal pancreatic tissues. HPA immunohistochemistry confirmed *CTNNB1* shifts from membranous to cytoplasmic localization in PDAC, indicating activated Wnt signaling and concordant dysregulation at mRNA and protein levels. *CTNNB1* protein was mainly localized in malignant epithelial cells of PDAC, with elevated expression also observed in partial immune cell subsets by single‐cell sequencing and spatial transcriptome assessment. Upregulated *CTNNB1* was significantly linked to unfavorable clinical prognosis in PDAC. ROC analysis verified its good diagnostic efficiency for distinguishing PDAC from peritumoral tissues with favorable sensitivity and specificity. *CTNNB1* expression was closely correlated with multiple immune biomarker molecules and ICB therapeutic efficacy; patients with low *CTNNB1* expression tended to be more responsive to ICB treatment. Pharmacological prediction revealed that PDAC patients with high *CTNNB1* expression exhibited enhanced sensitivity to clofibrate and several other small‐molecule compounds.

**Conclusion:**

*CTNNB1* may be remarkably effective as a suitable biomarker for the prognosis prediction of PDAC patients and is closely related to the tumor immune microenvironment. Such an association could provide novel thoughts for the therapy tactics and mechanistic studies toward PDAC.

## 1. Introduction

Pancreatic cancer represents a significant contributor to global cancer mortality, with its elevated prevalence attributed to a multitude of risk factors. Over the last 25 years, the worldwide burden of pancreatic cancer has more than doubled [1–3]. Genetic mutations in cancer‐causing genes and common variants identified through genomewide association studies play crucial roles in the susceptibility to pancreatic cancer, influencing its advancement and metastasis. Pancreatic ductal adenocarcinoma (PDAC) is a prevalent form of pancreatic cancer, representing a significant portion of cases, known for its high malignancy and unfavorable prognosis. Identifying the genetic alterations associated with PDAC not only enhances our understanding of its pathogenesis but also presents prospects for informing early detection methods and prognostic assessment [4–6]. *CTNNB1*, a gene responsible for encoding β‐catenin, is pivotal in the Wnt signaling pathway and serves as a central downstream effector in the canonical Wnt signaling cascade [7, 8]. Dysregulation of the *CTNNB1* gene expression has been implicated in the pathogenesis of several cancers, such as endometrial cancer, colorectal cancer, and hepatocellular carcinoma (HCC) [9–11]. *CTNNB1* mutations are detected in 20%–39% of HCC cases, primarily located at serine/threonine sites or neighboring amino acids within *CTNNB1* exon 3 (93.38%). These mutations commonly trigger Wnt/β‐catenin (Wnt‐on) activation. Consequently, β‐catenin relocates from the cytoplasm to the nucleus, facilitating the activation of target genes through its interaction with T‐cell factor (TCF) family proteins [12–15]. In colorectal cancer, β‐catenin signaling is closely associated with malignant progression, and pharmacological modulation of this pathway has been shown to inhibit the proliferative capacity of colon cancer cells [16]. Genetic investigations have further verified that *CTNNB1* genetic variants are significantly correlated with gastric cancer susceptibility and clinical prognosis in the Chinese population [17]. In breast cancer, the increased expression of β‐catenin helps maintain the self‐renewal ability of cancer stem cells and promotes the secretion of MMP‐related proteins, thereby facilitating tumor invasion and the development of drug resistance [18]. During embryonic prostate development, aberrant β‐catenin overactivation contributes to the development of invasive prostate cancer, and such abnormal upregulation is predominantly induced by *CTNNB1* mutations and the dysregulated expression of upstream and downstream Wnt pathway modulators [19, 20]. In glioblastoma (GBM), excessive Wnt/β‐catenin activation is tightly linked to unfavorable patient survival outcomes. β‐Catenin phosphorylation promotes glioma cell migration and activation, and its binding to cadherin further enhances tumor motility and epithelial–mesenchymal transition (EMT), accelerating malignant progression [21–24]. Some studies have also identified rare *CTNNB1* mutations (c.94G > C, p.D32H) in perivascular epithelioid cell tumors, which can aid in diagnosis [25]. However, the clinical prognostic significance of *CTNNB1* expression and its specific association with the tumor immune microenvironment (TIME) remain poorly elucidated in PDAC. Accordingly, the present study systematically investigated the prognostic value of *CTNNB1* and explored its potential immunomodulatory roles in PDAC, aiming to provide novel insights into immune regulatory mechanisms and immunotherapeutic targets for this devastating disease.

## 2. Methods

### 2.1. Sample Collection and Organization

Tissue samples included in this study were retrospectively collected from patients diagnosed with PDAC at Nantong First People’s Hospital between January 2019 and December 2024. Two independent local formalin‐fixed paraffin‐embedded (FFPE) cohorts were established for genomic sequencing and immunohistochemical validation, respectively. First, a total of 10 pathologically confirmed PDAC patients were retrospectively enrolled. For each case, paired FFPE tumor and adjacent nontumor pancreatic tissues from the same patient were retrieved from the pathology archive for targeted next‐generation sequencing (NGS) analysis. All specimens were pathologically confirmed, and only samples with a tumor cell proportion ≥ 30% were included for subsequent DNA extraction and genomic analysis. Second, an independent cohort consisting of 36 FFPE PDAC specimens and matched normal pancreatic tissues was collected to characterize the protein expression and subcellular localization patterns of CTNNB1 in tumor and physiological pancreatic tissues. Unified inclusion criteria were as follows: (1) histopathologically confirmed primary PDAC; (2) no preoperative antitumor therapy; and (3) receipt of radical surgical resection and complete clinical follow‐up data. Patients with concurrent malignant tumors, severe autoimmune diseases, or neurological disorders that interfered with clinical follow‐up were excluded from this study. All experimental procedures were approved by the Ethics Committee of Nantong First People’s Hospital (Approval No. 2025‐KT295‐02).

RNA‐sequencing data, genetic variation information, and clinical follow‐up details for the uniformly standardized TCGA‐PAAD cohort and the normal pancreatic tissue cohort from GTEx were downloaded from the UCSC Xena database (https://xenabrowser.net/). The GEO cohorts GSE28735, GSE62452, and GSE71729, were obtained from the NCBI Gene Expression Omnibus (GEO, https://www.ncbi.nlm.nih.gov/geo/). A uniform inclusion and exclusion screening protocol was applied to all public cohorts. Inclusion criteria were: a definitive pathological diagnosis of pancreatic ductal adenocarcinoma, complete transcriptome expression data, and clear tissue annotation; control pancreatic tissue showed no tumors or organic lesions. Exclusion criteria were unknown pathological type, inflammatory or benign pancreatic lesions, missing clinical annotations, or poor sequencing quality.

### 2.2. DNA Extraction and NGS Detection of Genomic Alterations in Pancreatic Ductal Adenocarcinoma and Paired Normal Pancreatic Tissues

Genomic DNA was extracted from FFPE pancreatic cancer specimens using the AmoyDX FFPE DNA Kit (AmoyDX) according to the manufacturer’s standard protocols. DNA concentration was quantified via the dsDNA HS Assay Kit on a Qubit 3.0 fluorometer (Life Technologies), and DNA purity was further assessed using a NanoDrop 2000 spectrophotometer (Thermo Fisher Scientific). A standardized amount of genomic DNA (2000 ng) was prepared for subsequent library construction. Sequencing libraries were generated using the KAPA Hyper Prep Kit (KAPA Biosystems). Briefly, genomic DNA was mechanically fragmented into 200–350‐bp fragments with a Covaris M220 ultrasonicator (Covaris). Fragmented DNA was subjected to end repair, A‐tailing, and adapter ligation. The products were then PCR‐amplified and purified with Agencourt AMPure XP magnetic beads. For targeted gene enrichment, qualified DNA libraries were hybridized with customized xGen Lockdown Probes (Integrated DNA Technologies) covering 425 cancer‐related genes. The captured libraries were further amplified using the KAPA HiFi HotStart ReadyMix Kit (KAPA Biosystems). Final library quantification was performed using the KAPA Library Quantification Kit (KAPA Biosystems), and fragment size distribution was validated using an Agilent 2100 Bioanalyzer. All qualified libraries were finally sequenced on the Illumina HiSeq 4000 platform with paired‐end sequencing. The average target sequencing depth was maintained at approximately 700x, and raw sequencing data were collected using the official Illumina data acquisition software.

### 2.3. Processing and Analysis of Sequencing Data

Raw sequencing reads were first processed to remove adapter sequences, low‐quality bases, and duplicate reads. Clean reads were aligned to the human reference genome hg19 (GRCh37) using the Burrows–Wheeler Aligner (BWA, v.0.7.10 [25]). Local alignment optimization and variant calling were performed using the Genome Analysis Toolkit (GATK, v.3.2) and VarScan [26, 27]. Strict quality filtering was conducted via the VarScan fpfilter pipeline, and variants with an effective sequencing depth lower than 100× were excluded. The criteria for somatic variant detection were set as follows: at least five supporting reads for insertions and deletions (INDELs) and at least eight supporting reads for single‐nucleotide variants (SNVs). Variants with an allele frequency (AF) < 5% and those with significant strand bias were discarded. To filter out benign polymorphic variants, variants with a population frequency > 0.1% in the ExAC, 1000 Genomes, dbSNP, and ESP6500SI‐V2 databases were defined as single‐nucleotide polymorphisms (SNPs) and excluded [28]. Germline variants were further eliminated using matched adjacent normal tissue data, and only somatic mutations were retained. Variant annotation was performed using ANNOVAR and SnpEff (v.3.6), and the COSMIC database was applied to characterize oncogenic mutations. Structural DNA translocation variants were analyzed using Factera (v.1.4.3) [29]. Finally, somatic mutations in *CTNNB1* were screened and reserved for subsequent statistical analysis.

### 2.4. Differential Expression of *CTNNB1* in Pancreatic Ductal Adenocarcinoma

Gene expression data of pancreatic tissues were obtained from the UCSC Xena database, including the TCGA‐PAAD and GTEx normal pancreatic datasets based on RSEM‐normalized TPM values. A combined expression matrix was integrated for the subsequent analysis. To eliminate batch effects and unify data distribution, intradataset z‐score standardization was performed using the formula ((*x* − *μ*)/*σ*) to ensure uniform data units. Samples with |*z* score| > 3 were removed as outliers, corresponding to the 99.7% confidence interval (CI) of the normal distribution. Three independent GEO datasets (GSE28735, GSE62452, and GSE71729) were utilized for external validation. Raw microarray expression profiles were strictly quality‐controlled to exclude samples with missing expression values, incomplete annotation information, and invalid probes. Standard microarray preprocessing was performed sequentially, including background correction, quantile normalization, and log2 transformation to optimize data distribution. After uniform preprocessing, the expression values of the *CTNNB1* gene and corresponding tissue type annotations were extracted from all datasets. *Z*‐score normalization was applied within each dataset to eliminate dimensional differences and remove residual outliers (|*z* score| > 3). The Mann–Whitney *U* test (Wilcoxon rank‐sum test) was finally used to compare the differential expression levels of *CTNNB1* between pancreatic tumor tissues and normal pancreatic tissues.

### 2.5. Association of *CTNNB1* Expression With Clinical Stage and Grade in Pancreatic Ductal Adenocarcinoma

Gene expression data of *CTNNB1* (ENSG00000168036) for pancreatic ductal adenocarcinoma were extracted from the standardized TCGA Pan‐Cancer dataset using the UCSC Xena browser (https://xenabrowser.net/). Only samples annotated as primary tumors were included. Raw expression values were transformed with log_2_ (*x* + 0.001). One‐way analysis of variance (ANOVA) was used to evaluate overall expression differences across clinical stages and grade, and unpaired Student’s *t* test was applied for pairwise comparisons between groups.

### 2.6. Genomic Alterations of *CTNNB1* in Pancreatic Ductal Adenocarcinoma

Genomic alteration data of pancreatic ductal adenocarcinoma (PAAD) were downloaded from the Genomic Data Commons (GDC, https://portal.gdc.cancer.gov/). Somatic SNVs and small insertions/deletions were detected using the official GDC MuTect2 variant calling pipeline [30]. All raw variant calls were further filtered in accordance with standard GDC quality control criteria, removing low‐quality variants, germline polymorphisms, recurrent technical artifacts, and contaminated loci. Variants with low allelic frequency and insufficient supporting reads were excluded to screen and retain high‐confidence somatic mutations. After the integration of qualified mutation data, protein domain annotation information was extracted using the maftools R package. The cBioPortal database (https://www.cbioportal.org/) was further queried to systematically characterize the genomic alteration landscape of *CTNNB1* in PDAC. The overall alteration spectrum covering somatic mutations, gene amplification, and homozygous deletion events was summarized, and the alteration frequency and distribution patterns of *CTNNB1* within the PDAC cohort were statistically analyzed.

### 2.7. CTNNB1 Protein Expression Analysis

The streptavidin–peroxidase (SP) immunohistochemical method was used to detect the protein expression and subcellular localization of CTNNB1 in PDAC and normal pancreatic FFPE tissues. After deparaffinization, rehydration, and high‐pressure antigen retrieval with 0.01 mmol/L citrate buffer, tissue sections were treated with 3% hydrogen peroxide to block endogenous peroxidase activity. Sections were incubated with validated rabbit polyclonal anti‐CTNNB1 antibody (HPA029160, Atlas Antibodies, Sigma‐Aldrich), an HPA project‐validated antibody specific for FFPE sample detection, at a 1:100 dilution (37°C incubation followed by overnight incubation at 4°C). After repeated PBS washing, sections were incubated with secondary antibody, visualized via DAB chromogenic staining, counterstained with hematoxylin, and finally dehydrated, cleared, and mounted for microscopic observation. CTNNB1 positive expression was defined as cytoplasmic yellow–brown granular staining. Ten random high‐power fields were selected for quantitative scoring, combining staining intensity (0–3 points) and positive cell proportion (0–4 points). The final IHC score was calculated by multiplying the two scores, with total scores ≥ 3 defined as positive and < 3 as negative. For cross‐validation, IHC data of normal and PDAC pancreatic tissues tested with the identical HPA029160 antibody were retrieved from the Human Protein Atlas (HPA, https://www.proteinatlas.org/). The official HPA evaluation criteria were referenced to systematically compare the differences in the CTNNB1 protein expression level and subcellular distribution between normal and tumor pancreatic tissues. For clinical correlation analysis, the IHC expression status of CTNNB1 was further correlated with clinicopathological parameters in our internal cohort consisting of 36 PDAC patients, including patient age, maximum tumor diameter, and tumor differentiation grade. The chi‐square test was applied to evaluate the statistical associations between CTNNB1 positivity and different clinical indicators.

### 2.8. Assessment of *CTNNB1* Localization and Expression Using Single‐Cell Sequencing (scRNA‐Seq) in Pancreatic Ductal Adenocarcinoma Dataset GSE111672

The single‐cell RNA‐seq dataset GSE111672 was used to explore the cellular localization and expression pattern of *CTNNB1* in pancreatic cancer. All single‐cell transcriptomic data were retrieved from the TISCH2 database [31]. All datasets deposited in TISCH2 have undergone systematic preprocessing including strict quality control, expression normalization, PCA dimensionality reduction, uniform manifold approximation and projection (UMAP) embedding, and authoritative cell‐type annotation. Based on the precalculated UMAP coordinates, UMAP was applied to reduce the dimensionality of high‐dimensional single‐cell data and cluster cells according to distinct gene expression profiles. Cells were annotated and colored under three classification standards, including main cell lineage, refined cell subtype, and malignant or nonmalignant cell classification. Each dot in the UMAP plot represented an individual cell, and cell‐type labels were marked in the center of each cell cluster. Subsequently, the expression distribution of *CTNNB1* was visualized. The Kruskal–Wallis rank‐sum test was adopted to statistically assess the expression differences of *CTNNB1* among various cell types in pancreatic ductal adenocarcinoma.

### 2.9. Spatial Transcriptome Assessment of *CTNNB1* Expression and Spatial Distribution Characteristics in Pancreatic Ductal Adenocarcinoma Tissue Sections

The 10x Visium spatial transcriptomic Seurat objects of pancreatic cancer tissue slices were obtained from the Sparkle database. Based on the raw count matrix, LogNormalize normalization was performed with a scale factor of 10,000 to eliminate deviations caused by inconsistent sequencing depth. The SpatialFeaturePlot function in the Seurat package was used to map the expression level of *CTNNB1* to the spatial coordinates of tissue sections. Each Visium spot was approximately 55 μm in diameter and covered 1 to 10 cells, and the gradient color was applied to reflect the relative gene expression abundance. Spatial microenvironment deconvolution was conducted via the integrated Cottrazm analytical workflow embedded in SparkleDB. Firstly, Cottrazm‐BoundaryDefine was used to precisely delineate tumor boundaries by combining spatial transcriptomic profiles and H&E staining pathological images. Secondly, Cottrazm‐SpatialDecon integrated single‐cell transcriptomic reference profiles, spatial expression matrices, and spatial location information to predict the cellular composition proportion of each spot through deconvolution algorithm. Thirdly, Cottrazm‐SpatialRecon reconstructed cell type–specific gene expression profiles at subspot resolution [32, 33]. Two types of spatial visualization maps were constructed according to the deconvolution results. The dominant cell‐type distribution map was colored based on the most abundant cell type within each spot to exhibit the overall spatial distribution pattern of main cell populations. Meanwhile, the independent proportion distribution map of each cell subtype was generated, in which the cellular proportion values were mapped to tissue spatial coordinates. The color gradient ranging from white (proportion = 0) to designated hues clearly revealed the spatial enrichment characteristics of different cell subsets.

### 2.10. Prognostic Analysis

Univariate Cox regression analysis was performed using the survival package in R software (Version 4.3.2) to evaluate the association between *CTNNB1* expression and prognostic outcomes in pancreatic ductal adenocarcinoma, including disease‐free interval (DFI), disease‐specific survival (DSS), overall survival (OS), and progression‐free interval (PFI). Hazard ratios (HRs) and 95% CIs were calculated. For Kaplan–Meier survival analysis, the optimal cutoff value of *CTNNB1* expression was determined using the survminer package. Patients were stratified into high‐ and low‐expression groups, with a group size ratio ≥ 0.3 to ensure balanced distribution. Survival curves were plotted using the survfit function, and differences were assessed using the log‐rank test. A meta‐analysis based on the inverse variance method was conducted to integrate results from univariate Cox regression, with log HR as the effect size. An HR > 1 indicated a potential oncogenic role of *CTNNB1* in pancreatic ductal adenocarcinoma. All meta‐analyses were performed using the meta package in R. Additionally, receiver operating characteristic (ROC) curve analysis was performed using the pROC package to evaluate the diagnostic accuracy of *CTNNB1* expression in distinguishing pancreatic ductal adenocarcinoma tissues from normal tissues. The area under the curve (AUC) and 95% CI were calculated, and smooth ROC curves were generated.

### 2.11. Correlation Between *CTNNB1* Expression and the Immune Microenvironment in PDAC

To characterize the TIME of PDAC, the standardized TCGA‐PAAD dataset was downloaded from the UCSC Xena browser (https://xenabrowser.net/). Expression data of *CTNNB1* were extracted, and all values were log_2_‐transformed (log_2_ (*x* + 0.001). The expression matrix was further annotated and mapped to Gene Symbols. First, patients were divided into high‐ and low‐expression groups based on the median value of *CTNNB1* expression. The proportion of different immune subtypes [34] in each group was calculated, and differences were evaluated using the *χ* ^2^ test. Immune cell infiltration scores for each patient were quantified using five independent deconvolution algorithms implemented in the IOBR R package (Version 4.3.2), including TIMER [35], EPIC [36], MCPcounter [37], xCell [38], and CIBERSORT [39]. Immunomodulatory genes, including immunostimulators, immunoinhibitors, chemokines, and HLA family genes, were retrieved from the TISIDB database (https://cis.hku.hk/TISIDB/download.php). PDAC patients from the TCGA cohort were stratified into high‐ and low‐*CTNNB1* expression subgroups based on the median expression value of *CTNNB1*. The Spearman correlation analysis was performed to calculate the correlation coefficient between *CTNNB1* and each immune‐related gene, and Wilcoxon rank‐sum test was applied to verify the expression difference of these immunomodulators between two subgroups. Finally, correlation coefficients were visualized by heatmap, with asterisks indicating statistical significance (^∗^ *p* < 0.05, ^∗∗^ *p* < 0.01, ^∗∗∗^ *p* < 0.001). To visualize the association between immune responses and genomic states, all patients were stratified into four groups (Q1–Q4) according to quartiles of *CTNNB1* expression. Q1 represented patients with the highest 25% expression, while Q4 indicated those with the lowest 25%. Mean values of each signature score across the four subgroups were calculated, with missing values excluded. All results were visualized via heatmap using the pheatmap R package.

### 2.12. Predicting Immune Therapy Response

The study utilized Spearman correlation analysis to examine the correlation between *CTNNB1* and TIP scores, as well as the autocorrelation of TIP scores [40]. Relevant results were visualized using the linkET package (Version 0.0.5, https://github.com/Hy4m/linkET). The easier package was used to evaluate IFN‐γ levels in the TCGA‐PAAD cohort. Patients were divided into high‐ and low‐*CTNNB1* groups according to the median expression value, and differences in IFN‐γ scores between the two groups were compared using the Wilcoxon rank‐sum test [41]. The Tumor Immune Dysfunction and Exclusion (TIDE) algorithm (https://tide.dfci.harvard.edu/) was used to predict immunotherapy response in the TCGA‐PAAD cohort [42]. Samples were stratified by clinical stage and *Z* score standardized within each stratum. *CTNNB1* expression was compared between immunotherapy responders and nonresponders, with outliers (*Z* score > 3 or < −3) excluded. Group differences were evaluated using the Wilcoxon rank‐sum test. The Biomarker Evaluation module of the TIDE database was further applied to assess the predictive potential of *CTNNB1*, and bar plots were generated to display AUC values across datasets for distinguishing responders from nonresponders. All analyses were performed in R software (Version 4.3.2).

### 2.13. Candidate Drug Evaluation for Patients With High *CTNNB1* Expression

In this study, multicancer public matching cohorts, the TCGA pan‐cancer cohort, and multiple independent PAAD datasets from the GEO database were collected for candidate drug screening and validation. A ridge regression model based on the oncoPredict [43] (v 0.2) algorithm was applied to estimate clinical sample drug responses using baseline tumor cell line expression profiles and in vitro drug sensitivity data. The IC50 values from the GDSC1/2 databases and AUC values from the CTRP and PRISM databases were adopted to evaluate drug sensitivity. Meanwhile, for drug repositioning and transcriptional reversal analysis, Connectivity Map (cMAP) analysis was further conducted to identify small molecules capable of counteracting the oncogenic effect of *CTNNB1* in PDAC. The CMAP_gene_signatures RData file containing 1288 compound‐related signatures was used, and the XSum [44] method was applied to compare transcriptomic signatures between *CTNNB1* high‐ and low‐expression subgroups. All predicted drug sensitivity values were normalized via *Z*‐score standardization to eliminate dimensional differences. Only samples with complete paired gene expression and drug sensitivity data were included, while samples with missing values and outliers were excluded to reduce dataset heterogeneity and analytical bias. Unified interpretation criteria were applied: a negative *CTNNB1* expression and IC50/AUC values indicated higher drug sensitivity, whereas a positive correlation indicated drug resistance. A lower negative connectivity score in cMAP analysis suggested a stronger antagonistic effect against the transcriptional phenotype mediated by *CTNNB1* expression, indicating promising therapeutic potential of the corresponding drugs.

### 2.14. Statistical Analysis

All statistical analyses were performed using R software (v4.3.2), and a two‐tailed *p* < 0.05 was defined as statistically significant. The Mann–Whitney *U* test was used to compare *CTNNB1* expression between PDAC and normal pancreatic tissues, while one‐way ANOVA combined with unpaired Student’s *t* test was applied for comparisons across different clinical stages. For enumeration data, the chi‐square test was used for intergroup comparison. Spearman’s correlation analysis was used to evaluate the associations of *CTNNB1* expression with immune cell infiltration, immunomodulatory genes, TIP scores, and drug sensitivity AUC values.

## 3. Results

### 3.1. Genomic Variation and Expression Landscape of *CTNNB1* in PDAC

NGS was performed on PDAC tumor tissues and paired adjacent normal pancreatic tissues. A missense mutation of *CTNNB1* (c.134 C > T, p.Ser45Phe) located in exon 3 was identified in PDAC samples, with a mutation frequency of 25.71% (Table 1). Accordingly, *CTNNB1* was screened out as a key candidate gene, which was selected for subsequent comprehensive functional exploration in pancreatic cancer.

**TABLE 1:** Genomic alteration characteristics of major mutant genes in PDAC patients.

Gene	cDNA alteration	Protein changes	Variation type	Abundance
ERBB2	—	—	Copy number amplification mutation	CN = 7.45
TP53	c.227_279del	p.Ala76Valfs^∗^55	Frameshift mutation	4.97%
CTNNB1	c.134C > T	p.Ser45Phe	Missense mutation	25.71%
TERT	c.‐124C > T	—	Noncoding region mutation	19.04%
CDK12	—	—	Copy number amplification mutation	CN = 10.46
KMT2B	c.7580T > C	p.Leu2527Pro	Missense mutation	16.27%
PBRM1	c.897G > T	p.Met299Ile	Missense mutation	9.9%

**FIGURE 1:**
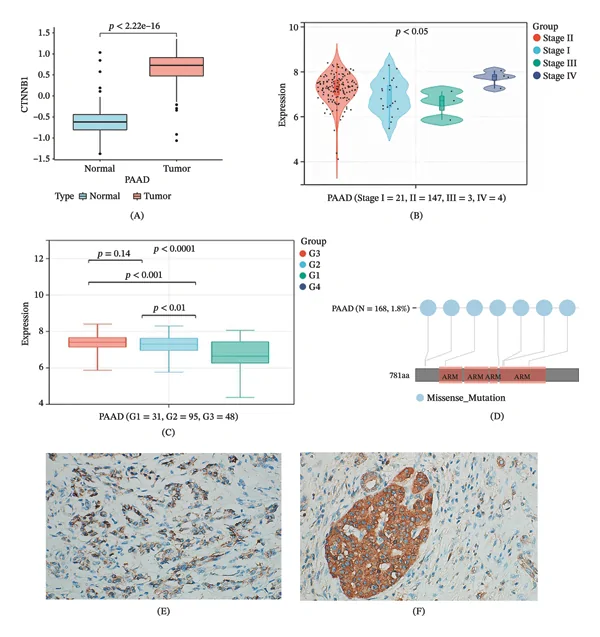
Genomic variation, transcriptomic expression landscape and subcellular localization of *CTNNB1* in PDAC. (A) Comparison of *CTNNB1* mRNA expression between normal pancreatic tissues and PDAC tissues from TCGA–GTEx cohort (PDAC tumor samples, *n* = 178; normal pancreatic samples combining TCGA adjacent nontumor tissues and GTEx specimens, *n* = 169). (B) *CTNNB1* expression across different clinical stages of PDAC. Sample sizes: Stage I = 21, Stage II = 147, Stage III = 3, Stage IV = 4. Note the limited sample size of Stage III and Stage IV subgroups. (C) *CTNNB1* mRNA expression across pathological differentiation grades (G1–G3), showing progressively higher expression in poorly differentiated high‐grade PDAC (G3). Sample sizes: *G*1 = 31, *G*2 = 95, *G*3 = 48. (D) Distribution of *CTNNB1* missense mutations. (E) Normal pancreatic tissue showing strong and continuous membranous expression of CTNNB1 in exocrine glandular cells. Scale bar: 400 μm. (F) PDAC tissue showing prominent cytoplasmic accumulation of CTNNB1 with attenuated membranous staining in tumor cells. Scale bar: 400 μm.

Integrated analysis of TCGA and GTEx datasets revealed significantly elevated *CTNNB1* expression in PDAC tissues compared with normal pancreatic tissues (Figure 1A). Three independent GEO cohorts (GSE28735, GSE71729, and GSE62452) were used for external validation, and consistent results confirmed higher *CTNNB1* expression in PDAC tissues relative to normal controls (Supporting Figure 1A). Clinical stratification analysis demonstrated that Stage IV patients exhibited significantly higher expression compared with Stage III patients (*p* < 0.01) and also showed significantly elevated expression relative to Stage I and II patients (^∗^ *p* < 0.05) (Figure 1B). Clinical stratification analysis based on pathological tumor grade demonstrated a progressive upward trend of *CTNNB1* mRNA expression from well‐differentiated G1 to poorly differentiated G3 lesions. *CTNNB1* expression was markedly higher in G3 tumors relative to G1 (*p* < 0.001) and G2 tumors relative to G1 (*p* < 0.01), while no significant difference was detected between G2 and G3 groups (*p* = 0.14) (Figure 1C). Genomic alteration analysis further verified that missense mutation constitutes the predominant genetic variation type of *CTNNB1* in PDAC (Figure 1D). To further characterize the protein‐level expression and subcellular localization of CTNNB1 in PDAC, we retrospectively collected 36 FFPE PDAC tissue blocks from our institutional pathology archive and performed immunohistochemical staining. For cross‐verification, we also analyzed public immunohistochemical data from the HPA database with the identical antibody (HPA029160) to systematically compare CTNNB1 staining profiles between normal pancreatic tissues and PDAC lesions. Consistent staining patterns were observed in both our in‐house tissue microarray and HPA database specimens. In normal pancreatic exocrine acinar cells, CTNNB1 was predominantly localized at the cell membrane, displaying intense, continuous membranous signals with faint cytoplasmic staining (Figure 1E; Supporting Figure 1B). This physiological membrane‐dominant distribution aligns with CTNNB1’s canonical function as a cell–cell adhesion molecule [45]. In contrast, PDAC tissues presented striking pathological remodeling of CTNNB1 subcellular localization: tumor cells showed attenuated, discontinuous membranous staining alongside markedly elevated, diffuse cytoplasmic accumulation of CTNNB1 (Figure 1F; Supporting Figure 1C). The subcellular redistribution of CTNNB1 from membrane dominance to cytoplasmic enrichment represents a hallmark of aberrant Wnt/β‐catenin pathway activation [46]. We further quantified the correlation between CTNNB1 IHC positivity and clinical pathological parameters in our local cohort, as summarized in Table 2, which demonstrated that CTNNB1 protein positivity was not significantly associated with patient age (*p* = 0.968) or tumor maximum diameter (*p* = 0.1664). However, CTNNB1 IHC positivity exhibited a striking correlation with tumor differentiation status (*χ* ^2^ = 3.841, *p* = 0.0017). This distribution pattern is consistent with the transcriptomic trend shown in Figure 1C. Collectively, the consistent dysregulation of CTNNB1 at both transcriptomic and proteomic levels comprehensively validates its aberrant expression patterns in PDAC, providing multiomics evidence to support its oncogenic function during pancreatic cancer progression.

**TABLE 2:** Correlation between CTNNB1 immunohistochemical status and clinicopathological features [*n* (%)].

Clinicopathological variables	Number of cases	CTNNB1		*χ* ^2^/*p*
		Positive (*n* = 22)	Negative (*n* = 14)	
Age (years)				
< 50	13	8 (61.5%)	5 (38.5%)	0.0016/0.968
≥ 50	23	14 (60.9%)	9 (39.1%)	
Tumor diameter (cm)				
≤ 2	13	6 (46.2%)	7 (53.8%)	1.915/0.1664
> 2	23	16 (69.6%)	7 (30.4%)	
Tumor differentiation				
Moderately/poorly differentiated	24	19 (79.2%)	5 (20.8%)	3.841/0.0017
Well differentiated	12	3 (25.0%)	9 (75.0%)	

### 3.2. Single‐Cell and Spatial Transcriptomic Characterization of *CTNNB1* Localization in PDAC

he UMAP analysis of expression profiles clearly distinguished distinct cell populations within pancreatic cancer samples Based on Single‐Cell Sequencing Dataset (GSE111672) (Figure 2A). Notably, *CTNNB1* exhibited significant differential expression across the identified cell clusters (Figure 2B). Further examination of single‐cell expression patterns revealed that *CTNNB1* was predominantly expressed in malignant cells and proliferating T cells (Figure 2C), with high expression levels observed in both the malignant and immune cell profiles (Figure 2D). These data suggest that *CTNNB1* might work as a pivotal participant in the cancerous process of pancreatic cancer and their immune signal pathway. The spatial transcriptomic profiling demonstrated that *CTNNB1* transcriptional distribution was spatially consistent with tumor cell‐enriched regions in PAAD primary tissue (Figure 2E,F; specimen GSM6505133, GSE211895). To quantify regional expression differences of *CTNNB1*, we segmented the PAAD tissue slice into three histological microregions, namely, malignant tumor area, tumor‐normal mixed transitional area, and normal pancreatic parenchymal area. Quantitative statistics in Figure 2G confirmed significantly elevated *CTNNB1* mRNA abundance within malignant regions relative to mixed and normal compartments (all *p* < 0.001). Subsequent cell‐type deconvolution based on four independent PAAD transcriptomic datasets (PAAD [GSM6177618, GSE203612]; PAAD2/3/4 [GSM6505133/GSM6505134/GSM6505135, GSE211895]) further validated that *CTNNB1* overexpression was restricted to tumor cell‐dominant niches (Figure 2H). Of note, cell infiltration quantification revealed a typical immunosuppressive TME across enrolled PAAD specimens, characterized by abundant tumor and myeloid cell infiltration accompanied by robust depletion of adaptive T/B/NK lymphocytes. Collectively, our spatial multievidence confirmed preferential *CTNNB1* overexpression in malignant lesions of PDAC, implying the critical involvement of *CTNNB1* in pancreatic malignant transformation. These findings provide novel mechanistic clues for *CTNNB1* in PDAC progression and support its further exploration as a potential diagnostic and prognostic biomarker for PDAC clinical management.

FIGURE 2
*CTNNB1* expression landscape in pancreatic ductal adenocarcinoma revealed by single‐cell RNA sequencing and spatial transcriptomics. (A) UMAP plot showing the classification of major cell lineages in PDAC single‐cell samples (GSE111672). (B) UMAP visualization of the distinct expression pattern of *CTNNB1* across various cell lineages. (C) Comparative analysis of *CTNNB1* expression levels among different cell types. (D) Differential expression profiles of *CTNNB1* in immune, stromal, and malignant cell lineages. (E) Spatial localization plots of 11 distinct tumor, stromal, and immune cell subsets derived from a single PAAD primary tumor section (specimen GSM6177618 from dataset GSE203612); gradient red dots represent relative enrichment abundance of each cell population across spatial transcriptomic spots. (F) Spatial expression landscape of *CTNNB1* gene on the identical PAAD tissue slice; color gradient from blue to red corresponds to gradually increased *CTNNB1* transcriptional expression. (G) Histological spatial segmentation of the above PAAD specimen into three microregions: Malignant tumor area, mixed transitional area, and normal pancreatic parenchymal area (left and middle panels). Right bar chart quantifies the average *CTNNB1* expression among three subgroups; one‐way ANOVA was applied for statistical analysis, *p* < 0.001 for all pairwise comparisons. (H) Left heatmap: *Z*‐score normalized infiltration values of 11 cellular subsets across four independent PAAD (GSM6177618, dataset GSE203612), PAAD2/PAAD3/PAAD4 (GSM6505133/GSM6505134/GSM6505135, dataset GSE211895). Warm color indicates elevated cell infiltration (*Z* > 0), cold color represents reduced infiltration (*Z* < 0), and blank NaN means undetected cell population. Middle horizontal bar graph shows the *Z*‐score distribution of all cell subtypes in the PAAD2 sample.

**Figure:**
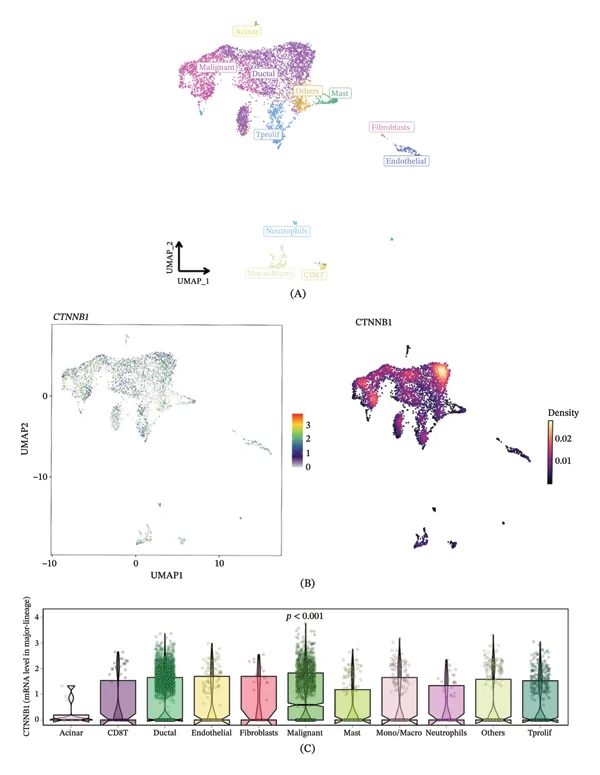

**Figure:**
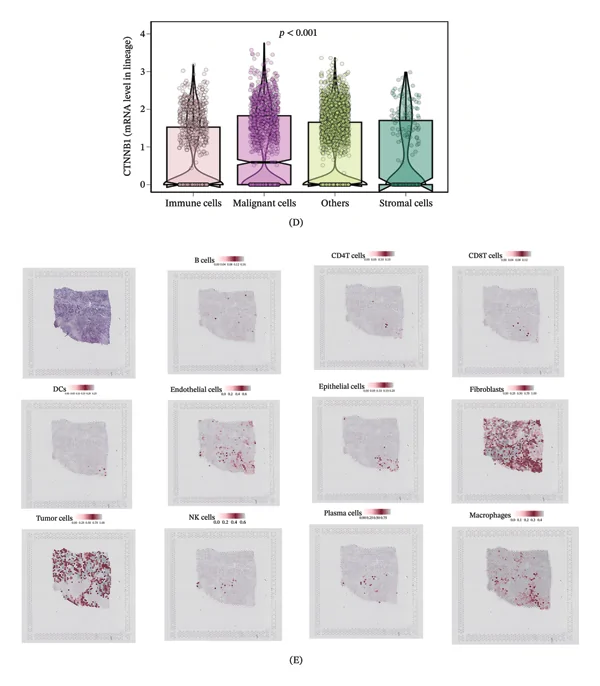

**Figure:**
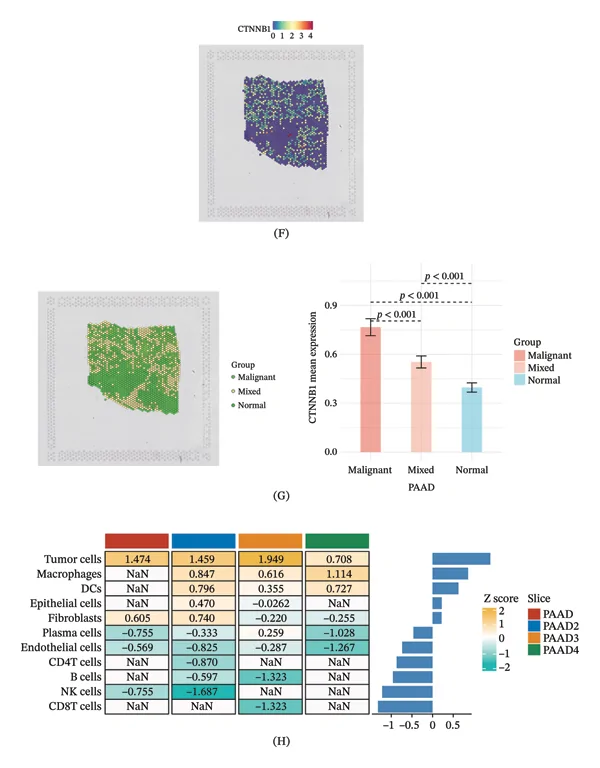

### 3.3. Correlation Between *CTNNB1* Expression and Prognosis in PDAC and Independent Cohort Validation

Univariate cox regression analysis of *CTNNB1* expression revealed its association with poorer prognosis in pancreatic cancer. High *CTNNB1* expression correlated with shorter DFI (*p* = 0.001, HR = 5.57, 95% CI 1.96–15.89), DSS (*p* = 0.005, HR = 2.12, 95% CI 1.25–3.58), OS (*p* = 0.003, HR = 2.00, 95% CI 1.27–3.15), and PFI (*p* < 0.001, HR = 2.47,95% CI 1.60–3.81) (Figure 3A). The above prognostic correlations were further verified by Kaplan–Meier survival curves with log‐rank test (Figure 3B). Meta‐analysis of the one‐way Cox regression models for the four survival endpoints confirmed that high *CTNNB1* expression was an independent risk factor for adverse prognosis in PAAD (combined HR = 2.33, 95% CI 1.80–3.03, *p* < 0.001), with no significant heterogeneity observed among the included endpoints (*I* ^2^ = 9%, *p* = 0.35) (Figure 3C). For diagnostic efficacy evaluation, ROC analysis of the TCGA and GTEx datasets demonstrated that *CTNNB1* expression had excellent ability to distinguish PAAD tumor tissues from normal pancreatic tissues (AUC = 0.976, 95% CI: 0.955–0.992) (Figure 3D). The calibration curve and Hosmer–Lemeshow goodness‐of‐fit test further validated the excellent fitting effect and reliability of the *CTNNB1*‐based diagnostic prediction model (Figure 3E). Finally, independent validation using the GEO dataset GSE57495 further corroborated the significant association between high *CTNNB1* expression and poorer OS in PDAC patients (log‐rank *p* = 0.003) (Figure 3F).

FIGURE 3Prognostic correlation, diagnostic efficacy evaluation, and independent validation of *CTNNB1* expression in pancreatic ductal adenocarcinoma (PDAC; termed PAAD in the TCGA database). (A) Forest plots of univariate Cox regression assessing the correlations between *CTNNB1* expression and four clinical endpoints (DFI, DSS, OS, and PFI) of PDAC, with corresponding HR, 95% CI and *p* values displayed. (B) Kaplan–Meier survival curves with log‐rank tests stratified by *CTNNB1* high/low expression for four PDAC survival endpoints (DFI, *n* = 68; DSS, *n* = 166; OS, *n* = 172; PFI, *n* = 171). (C) Meta‐analysis based on random‐effects model for four survival endpoints; pooled HR = 2.33 (95% CI: 1.80–3.03). (D) ROC curve evaluating the diagnostic performance of *CTNNB1* to discriminate PDAC tumor from normal pancreatic tissues (data from TCGA combined with GTEx). (E) Calibration curve of the *CTNNB1*‐based diagnostic prediction model showing favorable calibration (Hosmer–Lemeshow *p* = 0) (data from TCGA combined with GTEx). (F) External independent validation of the prognostic implication of *CTNNB1* in the GSE57495 PDAC cohort, log‐rank *p* = 0.003 for overall survival (OS).

**Figure:**
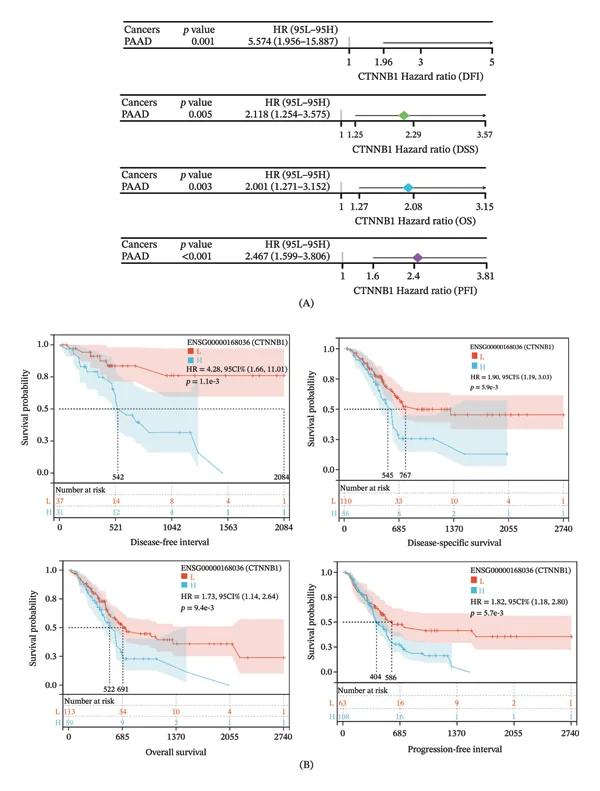

**Figure:**
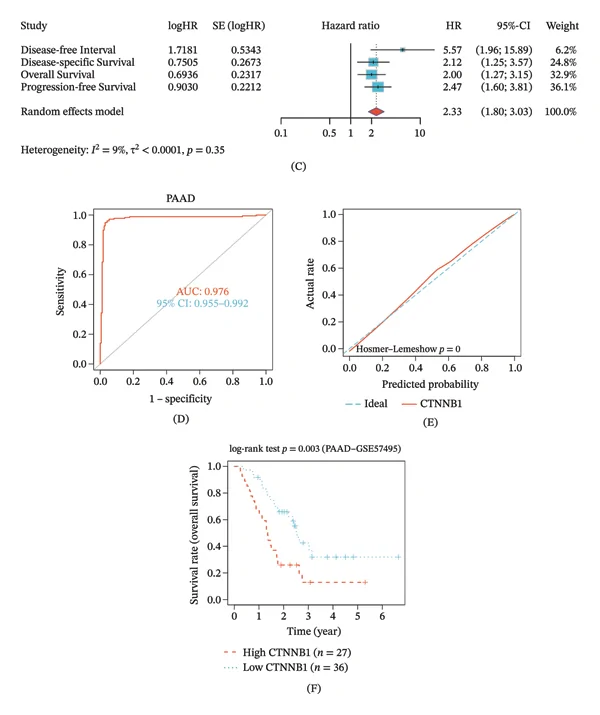

### 3.4. Crosstalk Between *CTNNB1*, Tumor Immune Microenvironment and Immunotherapy Efficacy

Large‐scale immunogenomic analysis of PDAC tumor samples was conducted in the TCGA database. The results of tumor immune subtypes showed that the high‐expression group of *CTNNB1* was mainly enriched with the C1 tissue healing immune subtype, while the low‐expression group was mainly enriched with the C3 inflammatory immune subtype (Figure 4A). The PDAC microenvironment score was estimated based on the expression level of *CTNNB1*. The results showed that the expression of *CTNNB1* was significantly positively correlated with both stromal score (*N* = 177, *R* = 0.32, *p* = 1.6e − 5) and ESTIMATE score (*N* = 177, *R* = 0.23, *p* = 2.0e − 3) (Figure 4B). The TIMER algorithm analysis shows that the expression level of *CTNNB1* is significantly positively correlated with six key immune cells in the tumor microenvironment: It was strongly positively correlated with CD8^+^ T cells (*r* = 0.57, *p* = 1.2e − 16), dendritic cells (DCs) (*r* = 0.54, *p* = 4.9e − 15), and neutrophils (*r* = 0.52, *p* = 8.3e − 14). It was moderately positively correlated with macrophages (*r* = 0.46, *p* = 1.4e − 10) and B cells (*r* = 0.37, *p* = 4.2e − 7), and weakly positively correlated with CD4^+^ T cells (*r* = 0.17, *p* = 0.02) (Supporting (Figure 2A) TIMER). The EPIC algorithm further verified the above trend: The expression of *CTNNB1* was associated with tumor‐associated fibroblasts (CAFs, *r* = 0.53, *p* < 0.001), macrophages (*r* = 0.27, *p* < 0.01), and NK cells (*r* = 0.27, *p* < 0.01); CTNNB1 expression was significantly positively correlated with the abovementioned cell populations, whereas it showed a significant negative correlation with other cell subsets (*r* = −0.45, *p* < 0.001), These results were overall consistent with the positive‐correlation trend revealed by the TIMER algorithm (Supporting (Figure 2A), EPIC). The MCPcounter algorithm confirmed this association in a broader range of cell types: the expression of *CTNNB1* was significantly positively correlated with 10 immune and stromal cell subsets, among which fibroblasts (*r* = 0.54, *p* < 0.001), neutrophils (*r* = 0.45, *p* < 0.001),and monocytic lineage cells (*r* = 0.44, *p* < 0.001) with fibroblasts exhibiting the strongest correlation, and the correlations with CD8^+^ T cells, DCs, etc., also corroborated the TIMER results (Supporting (Figure 2A), MCPcounter). We further conducted verification using CIBERSORT and xCell (Supporting (Figure 2A), CIBERSORT and xCell). The results of the two algorithms were consistent with the above quantitative analysis trends: In the high‐expression group of *CTNNB1*, the infiltration levels of CD8^+^ T cells, DCs, macrophages, neutrophils, and stromal fibroblasts were all significantly increased. Meanwhile, xCell analysis showed that the immune score, stromal score, and comprehensive score of the tumor microenvironment increased simultaneously, suggesting that the overall immune and stromal infiltration degree of the tumor microenvironment was significantly enhanced. We next evaluated the correlation of *CTNNB1* with immunomodulatory genes covering immunostimulators, chemokines, immune inhibitors, and HLA genes (Supporting (Figure 2A). Elevated *CTNNB1* was positively associated with numerous immunostimulatory genes (CD276, TNFRSF14/25) and proinflammatory CXCL chemokines, accompanied by upregulation of canonical HLA subtypes (HLA‐A, HLA‐DRA). Conversely, several CCL chemokines and inhibitory noncanonical HLA‐G were suppressed upon high *CTNNB1*. Most importantly, key immunosuppressive genes TGFB1, TGFBR1, and PVRL2 were significantly positively correlated with *CTNNB1*. Multiomics association analysis demonstrated that *CTNNB1* modulates immune genes via transcriptional regulation, DNA methylation, and copy number variation at genomewide scale (Supporting (Figure 2C). Finally, by comparing the immune responses and genomic instability‐related indicators of the two groups, it was found that there were significant molecular phenotypic differences in the high and low *CTNNB1* groups (Supporting (Figure 2D). Cross‐validation by multiple algorithms and association analysis of immune regulatory molecules jointly suggest that abnormal expression of *CTNNB1* can widely regulate the immune infiltration pattern in the tumor microenvironment of PDAC and may play an important role in mediating immune evasion.

FIGURE 4Correlations of *CTNNB1* with tumor immune microenvironment and immunotherapy response in PDAC. (A) Differences in PDAC immune subtypes between *CTNNB1* high/low expression groups based on the TCGA‐PAAD cohort (*n* = 149). (B) Scatter plots showing Spearman correlation between *CTNNB1* expression and StromalScore/ESTIMATEScore from ESTIMATE algorithm (*n* = 177). (C) Correlation landscape between *CTNNB1* and seven‐step antitumor immune cycle calculated via TIP algorithm. Orange lines represent statistically positive correlation, and green lines denote statistically negative correlation (*p* < 0.05); the middle heatmap exhibits pairwise correlation coefficients among distinct immune steps of cancer immunity cycle, with red for positive correlation and blue for negative correlation. (D) Combined density distribution and box plot of estimated IFN‐γ signature expression between *CTNNB1*‐high (red) and *CTNNB1*‐low (blue) subgroups in PDAC. Statistical difference was calculated by the Wilcoxon rank‐sum test (*p* = 0.017). (E) Wilcoxon rank‐sum test showing significantly elevated *CTNNB1* mRNA expression in nonresponder (NR) versus responder (R) patients receiving immune checkpoint blockade (*p* = 0.006). (F) Bar‐AUC plot summarizing the predictive efficiency of *CTNNB1* and canonical ICB predictive biomarkers across multiple published independent immunotherapy cohorts from diverse cancer types. Each colored horizontal line corresponds to an individual clinical dataset labeled in the right legend; the *X* axis denotes the AUC value, and the vertical black line at AUC = 0.5 represents the random prediction baseline.

**Figure:**
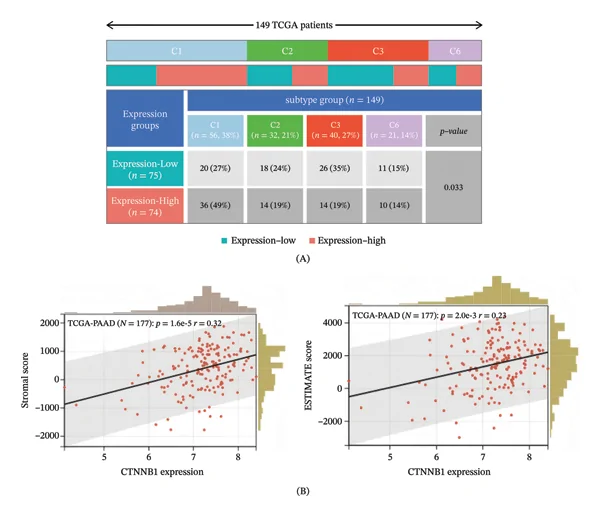

**Figure:**
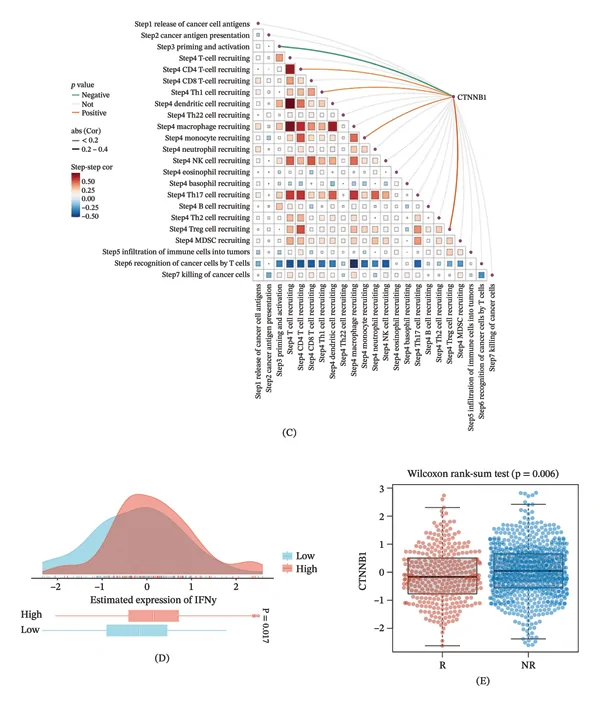

**Figure:**
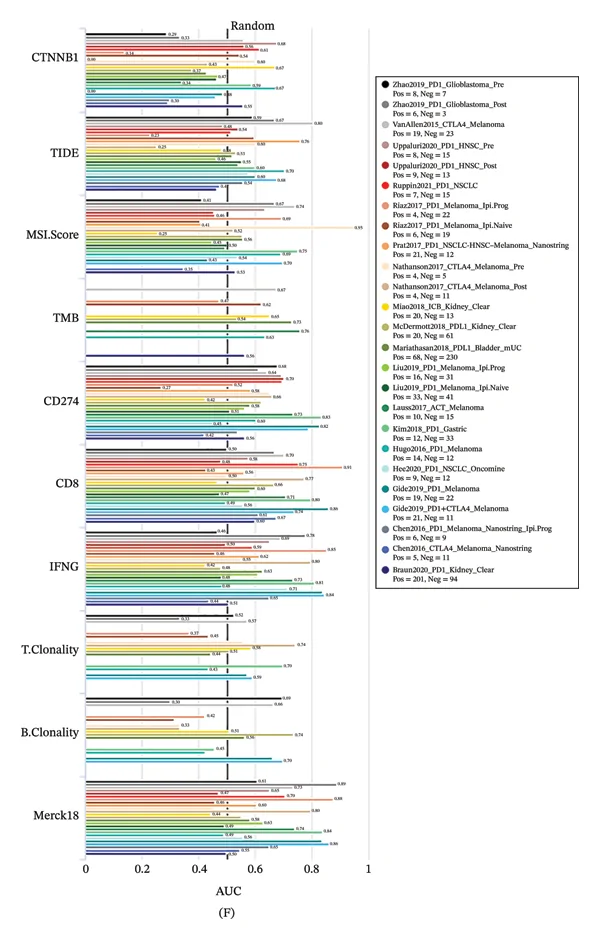

TIP‐based cancer immunity cycle analysis revealed that *CTNNB1* was positively correlated with Step4 immune cell recruitment but negatively associated with priming and activation (Step 3) (Figure 4C). The IFN‐γ signature score was significantly elevated in the *CTNNB1*‐high subgroup compared with the low‐expression counterpart (Figure 4D). Due to the scarcity of independent PDAC immunotherapy datasets, we adopted pan‐cancer clinical cohorts to evaluate the predictive value of *CTNNB1*. The results showed significantly different *CTNNB1* expression between immunotherapy responders and nonresponders, and lower *CTNNB1* levels tended to correlate with favorable immune checkpoint blockade (ICB) response (Figure 4E). We further explored the predictive performance of *CTNNB1* for ICB response across multiple independent published immunotherapy cohorts covering multiple cancer types. The AUC analysis suggested that the predictive efficiency of *CTNNB1* might be comparable to well‐established biomarkers including TIDE, TMB, *PD-L1* (*CD274*), *IFNG*, and *CD8*, with some cohorts presenting AUC values over 0.6 (Figure 4F). These results indicated that *CTNNB1* bears promising potential as a pan‐tumor predictive biomarker for ICB response, which may aid preliminary screening for patients with prospective clinical benefits from ICB. We further conducted computational drug sensitivity screening to identify candidate compounds suitable for *CTNNB1*‐high PDAC patients (all relevant analytical results are summarized in Supporting Figure 3). Pharmacogenomic profiling based on four independent drug response databases uncovered numerous targeted agents whose tumor sensitivity was closely correlated with *CTNNB1* expression, and these expression–drug associations were consistently validated across multiple external PDAC datasets. Additional CMap perturbation analysis indicated that clofibrate could reverse the oncogenic transcriptional signature triggered by abnormal *CTNNB1* activation.

## 4. Discussion

PDAC is a highly aggressive malignancy with a dismal 5‐year survival rate of less than 9% [47]. The high morbidity and mortality associated with PDAC have motivated extensive research into its pathogenic mechanisms and therapeutic strategies. In recent years, the translational application of immune checkpoint inhibitors (ICIs) has opened new avenues in cancer treatment, significantly altering the management of certain malignancies [48, 49]. In this context, the identification of novel immune checkpoint molecules and prognostic biomarkers holds critical clinical and scientific value for optimizing treatment protocols and improving outcomes for PDAC patients.

In the present study, high‐throughput NGS was performed to profile 425 tumor‐associated genes in PDAC and matched adjacent normal tissues. We identified a missense variant located in exon 3 of *CTNNB1*, which encodes the β‐catenin protein. β‐Catenin is a core component of the cytoadhesive complex that bridges cadherin molecules and the actin cytoskeleton. It maintains epithelial integrity and regulates intercellular adhesion, communication, and signal transduction, which are essential for embryogenesis and tissue homeostasis [50–52]. As a key mediator of the Wnt/β‐catenin pathway, exon 3 linker‐region mutations in *CTNNB1* prevent β‐catenin phosphorylation and subsequent ubiquitin‐dependent degradation, thereby prolonging its intracellular half‐life. In the canonical Wnt pathway, Wnt ligand–receptor binding suppresses the GSK3β‐mediated degradation of β‐catenin. Stabilized β‐catenin translocates into the nucleus, binds to LEF/TCF transcription factors, and initiates the transcription of downstream target genes. Functionally, *CTNNB1* modulates cell proliferation, differentiation, and migration, and its dysregulation contributes to tumorigenesis, metastasis, and immune evasion [53, 54]. Previous evidence has demonstrated that MYH9 promotes peritoneal metastasis in gastric cancer by binding to the promoter region of *CTNNB1* and activating its transcription [55]. Moreover, exon 3 mutations of *CTNNB1* drive endometrial carcinogenesis via dysregulating the Wnt/β‐catenin cascade and downstream transcriptional programs, and such mutations correlate closely with poor clinical outcomes including elevated recurrence and shortened OS [56]. Consistent with the above results, multidatabase analyses verified elevated *CTNNB1* expression in PDAC. Our 36‐case in‐house IHC cohort further confirmed that CTNNB1 protein positivity correlated only with tumor differentiation rather than age or tumor diameter, consistent with the mRNA trends across pathological grades. Combined with HPA database cross‐verification, we identified a clear subcellular shift of CTNNB1 from intact membrane staining in normal pancreatic tissue to prominent cytoplasmic accumulation in PDAC, a well‐recognized marker of abnormal Wnt pathway activation that may underlie the dedifferentiation of pancreatic tumor cells. Single‐cell transcriptome data (GSE111672) revealed predominant *CTNNB1* enrichment in malignant and immune cell subsets. Spatial transcriptomics further demonstrated markedly higher *CTNNB1* abundance within malignant microregions of PDAC tissue slices. Collectively, these multiomics findings imply that *CTNNB1* participates in PDAC malignant progression and tumor immune regulation, laying a foundation for its development as a promising diagnostic and prognostic biomarker for PDAC.

*CTNNB1* exerts crucial prognostic implications in a wide spectrum of human malignancies. Aberrant *CTNNB1* activation potentiates the proliferative ability and malignant aggressiveness of colorectal cancer cells, ultimately leading to dismal [57]. Wang et al. conducted a genetic association analysis of five *CTNNB1* tagging single‐nucleotide polymorphisms (tSNPs), including rs4135385, rs1798808, rs1880481, rs11564465, and rs2293303 and validated that *CTNNB1* genetic variants can serve as predictive indicators for cancer susceptibility and clinical [17]. In line with these previous studies, our analyses demonstrated that elevated *CTNNB1* expression is an independent risk factor (HR > 1) for unfavorable survival outcomes in PDAC, which was further supported by our meta‐analysis results. ROC curve analysis confirmed the high diagnostic efficiency of *CTNNB1* expression for discriminating PDAC lesions from normal pancreatic tissues. Calibration curves and goodness‐of‐fit tests demonstrated no significant deviation from optimal fitting, indicating stable and reliable predictive performance. Moreover, external validation using the GSE57495 dataset verified that high *CTNNB1* expression was significantly associated with shortened OS. Taken together, these findings suggest that *CTNNB1* possesses considerable potential as a promising diagnostic and prognostic biomarker for PDAC.

The TIME consists of malignant cells, diverse immune subsets, stromal cells, cytokines, and soluble mediators, and crosstalk among these constituents shapes the overall efficacy of antitumor immunity. Accumulating studies have demonstrated that the tumor microenvironment is a key driver of PDAC progression [58, 59]. Immunotherapeutic strategies are designed to reinstate competent antitumor immunity predominantly mediated by cytotoxic T lymphocytes (CTLs). Nevertheless, multiple immunosuppressive populations, including regulatory T cells (Tregs), myeloid‐derived suppressor cells (MDSCs), tumor‐associated macrophages (TAMs), and ILC2s, potently dampen antitumor effector responses and facilitate the formation of an immune‐exhausted tumor milieu [60]. Tumor cells with aberrant *CTNNB1* activation can secrete multiple chemokines and cytokines, including *CCL2* and *IL-6*. These mediators recruit and activate immunosuppressive populations, such as TAMs and MDSCs, thereby remodeling the TIME and fostering an immunosuppressive tumor milieu [61–63]. In this study, cross‐validation via well‐established deconvolution algorithms including TIMER, EPIC, MCPcounter, CIBERSORT, and xCell, combined with correlation analyses of immune modulatory molecules, revealed that *CTNNB1* expression was markedly associated with diverse immune cell infiltration abundance, immune regulatory genes, and chemokines within the PDAC microenvironment. Immune checkpoint molecules belong to coinhibitory signaling axes that preserve physiological immune tolerance, yet tumor cells frequently exploit these pathways to escape antitumor immune surveillance [64, 65]. ICI aims to revitalize the antitumor immune response by interrupting the coinhibitory signaling pathway and promote the elimination of immune‐mediated malignant cells. The most widely used targets of ICI consist of cytotoxic T lymphocyte–associated antigen‐4 (*CTLA-4*), programmed cell death protein 1 (*PD-1*), and programmed death‐ligand 1 (*PD* *-L1*) [66, 67]. Analyses based on the TIP database revealed that *CTNNB1* correlates with multiple antitumor immune events across distinct steps of the cancer–immunity cycle in PDAC. Restricted by insufficient PDAC‐specific datasets, we further performed pan‐cancer exploratory analysis and observed that reduced *CTNNB1* expression correlates with favorable immunotherapy responsiveness. Subsequent TIDE‐based AUC quantification across pan‐cancer cohorts demonstrated divergent predictive performance of *CTNNB1* among various malignancies. Collectively, patients with low *CTNNB1* expression tend to derive greater therapeutic benefits from ICIs, highlighting *CTNNB1* as a promising predictive biomarker for immunotherapy.

Notably, our data showed that patients with elevated *CTNNB1* exhibited higher IFN‐γ scores, whereas those with low *CTNNB1* expression displayed superior sensitivity to ICB. This discrepancy can be largely attributed to the distinctive immunosuppressive milieu and heterogeneous immune subtypes inherent to PDAC. IFN‐γ, a core cytokine secreted by activated T cells, NK cells, and NKT cells within the TME, orchestrates innate and adaptive antitumor immune responses [68]. Nevertheless, sustained IFN‐γ pathway activation triggers negative‐feedback loops that ultimately dampen antitumor immune potency [69]. As a critical component of such feedback, IFN‐γ directly upregulates surface *PD-L1* and *PD-L2* expression on neoplastic cells, infiltrating immune cells and stromal cells to initiate downstream *PD-1* inhibitory signaling. The engagement of these ligands with *PD-1* on tumor‐infiltrating T lymphocytes markedly suppresses T‐cell cytotoxic function [70–72]. Moreover, IFN‐γ drives the expression of multiple immunosuppressive mediators including *IDO1* across the tumor microenvironment [73]. Malignant cells exploit the dynamic equilibrium between proinflammatory and inhibitory cues to facilitate immune evasion and disease progression [74]. Accordingly, an isolated high IFN‐γ score fails to predict favorable ICB responsiveness. In terms of immune subtypes, patients with high *CTNNB1* preferentially fall into the C1 wound‐healing subtype, defined by abundant stromal fibrosis and chronic inflammatory infiltration. In this cohort, enhanced IFN‐γ signaling primarily stems from stroma‐derived chronic inflammation instead of functional tumor‐specific antitumor immunity [75]. Our data demonstrated that *CTNNB1* expression was significantly positively correlated with Treg recruitment and infiltration, indicating that elevated *CTNNB1* facilitates robust Treg accumulation within the tumor microenvironment. Tregs are key immunosuppressive lymphocytes responsible for driving ICB resistance across multiple solid malignancies. These cells directly suppress the proliferation and cytotoxic capacity of effector T cells and progressively abrogate tumor‐specific antitumor immunity [76, 77]. Collectively, patients with high *CTNNB1* display dual features of concurrent inflammatory activation and profound immune suppression: Elevated IFN‐γ signaling, accompanied by abundant Treg infiltration and upregulated immunosuppressive mediators including *CTLA4*, *PDCD1*, *TGFB1*, and *IL10*, synergistically shapes a highly inflamed yet potently immunosuppressive TME, which ultimately confers inherent resistance to ICB therapy. By contrast, low‐*CTNNB1* cases predominantly belong to the C3 inflammatory immune subtype, featuring reduced overall immunosuppressive load, limited Treg infiltration, and alleviated T‐cell exhaustion. Such immune landscape renders these patients more susceptible to ICB‐triggered antitumor immune activation and yields superior therapeutic responsiveness. As a central effector of the Wnt/β‐catenin cascade, aberrant nuclear translocation and persistent activation of *CTNNB1* enable its binding to *TCF/LEF* transcription factors, which drives robust transcription of numerous downstream target genes and remodels the PDAC TIME at multiple levels [78]. Upon persistent pathway activation, the *CTNNB1–TCF/LEF* complex serves as a pivotal transcriptional regulatory module. This complex restricts the infiltration of functional effector T cells into the TME, representing a key molecular mechanism underlying primary resistance to T cell–based immunotherapy in PDAC [79–83]. DCs are essential antigen‐presenting cells responsible for priming antitumor CD8^+^ T‐cell responses. Aberrant *CTNNB1* activation in DCs markedly impairs antigen cross‐presentation and cross‐priming, thereby dampening CD8^+^ cytotoxic T cell–dependent antitumor immunity [84, 85]. Combining our experimental findings with published evidence, *CTNNB1* facilitates robust recruitment and retention of Tregs in the TME via downstream *TCF7*‐driven transcriptional programming. These Tregs further upregulate an array of immunosuppressive effectors including *PD-L1*, *IDO1*, *CTLA4*, *TGF-β*, and *IL-10*, cooperating with the IFN‐γ negative‐feedback cascade to assemble a robust immunosuppressive network. Collectively, *CTNNB1* acts as a master upstream signaling switch; through the sequential axis “Wnt/β‐catenin ⟶ *TCF7* ⟶ accumulation of immunosuppressive cells ⟶ elevated inhibitory mediators”, it orchestrates global immunosuppressive remodeling in PDAC and confers inherent ICB resistance [86]. In summary, targeting the Wnt/*CTNNB1* cascade holds promising therapeutic potential to reverse PDAC‐associated immunosuppression and improve immunotherapy efficacy.

Given the critical role of aberrant *CTNNB1* overexpression in establishing an immunosuppressive TME and driving inherent ICB resistance in PDAC, screening small‐molecule compounds capable of counteracting *CTNNB1*‐driven oncogenic signatures represents a feasible translational strategy to overcome immunotherapeutic refractoriness. Pan‐cohort correlation screening identified multiple candidate targeted agents whose antitumor activity was inversely associated with *CTNNB1* expression; specifically, elevated *CTNNB1* conferred reduced sensitivity to distinct small‐molecule inhibitors from each database, including Golgicide‐A, PD‐313088 (PRISM), GDC‐0879, selumetinib (CTRP), as well as trametinib (GDSC1/GDSC2). To strengthen the clinical relevance of these preliminary pan‐cancer findings, we further validated the above drug–gene correlations in multiple independent PDAC transcriptomic cohorts including TCGA‐PDAC, GSE‐series, and E‐MTAB‐6134 datasets. Spearman correlation results consistently verified robust negative associations between *CTNNB1* expression and the efficacy of obidoxime, MK‐0752, AZD1332_1463, and Dasatinib_1079 across respective database‐matched PDAC subgroups. Obidoxime, a well‐known bis‐pyridinium reactivator, is a first‐line antidote against organophosphate pesticide and tabun poisoning. It also acts as an allosteric modulator of muscarinic receptors, preferentially targeting the M2 subtype via selective binding to regulate cholinergic signaling cascades [87]. Accumulating evidence has demonstrated that M2‐linked cholinergic signaling facilitates malignant proliferation across diverse human malignancies. Unfortunately, substantial antitumor effects of conventional M2 receptor antagonists are only achievable at extremely high concentrations far exceeding clinically feasible therapeutic dosages [88]. As a potent novel γ‐secretase inhibitor, MK‐0752 suppresses cell proliferation and triggers G2/M cell‐cycle arrest and apoptosis in ovarian cancer in a dose‐ and time‐dependent manner via downregulating *Notch1* and its downstream targets including *Hes1*, *XIAP*, *c-Myc*, and *MDM2*. Sequential administration of MK‐0752 following cisplatin pretreatment markedly augments ovarian cancer cell apoptosis and impedes subcutaneous xenograft growth in nude mice [89]. Of note, a multicenter Bayesian adaptive clinical trial verified that full recommended Phase II doses of the *Notch* γ‐secretase inhibitor MK‐0752 can be safely combined with gemcitabine for advanced PDAC; this combination yielded modest antitumor efficacy, while weekly MK‐0752 dosing exceeding 1800 mg failed to produce additional elevation in systemic drug exposure [90]. Previous studies have also demonstrated enhanced susceptibility to AZD1332_1463 among patients with high risk scores defined by 7‐methylguanosine‐associated prognostic signatures [91]. Dasatinib is a multitarget kinase inhibitor targeting the Src family kinases (SFKs), including Src, Fyn, Lyn, and Yes, which are frequently overexpressed or hyperactivated in KRAS‐mutant cancers, including the A549 cell line [92]. Furthermore, subsequent CMap validation based on the TCGA‐PAAD cohort further demonstrated that clofibrate possesses promising potential to antagonize the oncogenic effects driven by aberrant *CTNNB1* activation in PDAC. Taken together, the present study lays an informative foundation for subsequent targeted drug exploitation and personalized immunotherapy optimization against PDAC.

Nevertheless, several limitations of this study should be acknowledged. First, the NGS analysis was only performed on 10 pairs of FFPE‐treated tumor and normal tissue samples, with an insufficient sample size. We plan to expand the scale of the FFPE sample cohort in subsequent studies to verify the mutation spectrum and frequency distribution of the CTNNB1 gene in a larger PDAC population, and conduct multidimensional functional experiments to further clarify the role and mechanism of *CTNNB1* in pancreatic ductal adenocarcinoma. Second, all transcriptome analyses were conducted based on public datasets. Although we used our own clinical FFPE samples to validate the research results through IHC experiments, multicenter prospective studies are still required to confirm our conclusions. Third, patients with Stage III and IV disease were insufficient in the TCGA‐PAAD cohort; hence, the prognostic results should be validated using expanded clinical samples. Fourth, our single‐cell analysis is conducted using only the publicly available dataset GSE111672, potentially constraining the broader applicability of our results. In addition, immune cell infiltration was computationally predicted via TIMER, EPIC, MCPcounter, CIBERSORT, and xCell algorithms without in situ experimental confirmation. Moreover, the data for immunotherapy response analysis were retrieved from public databases and warrant validation using independent clinical specimens. Finally, the drug susceptibility screening was merely a bioinformatic prediction, and follow‐up pharmacological experiments are essential to confirm the therapeutic efficacy of candidate drugs in PDAC with high *CTNNB1* expression.

## 5. Conclusion

In conclusion, this study integrated multiomics datasets to systematically characterize the prognostic value of *CTNNB1* in PDAC, along with its regulatory functions in remodeling the TIME and modulating the response to ICIs. Using public pharmacogenomic resources, we further screened potential targeted agents associated with *CTNNB1* expression. Collectively, our results offer solid preclinical evidence to support prognostic stratification, personalized immunotherapy, and novel targeted drug development for PDAC.

## Funding

This study was supported by the National Natural Science Foundation of China (Grant No. 82172991); Nantong University Graduate Innovation Project (Grant No. YKC16070); and the Nantong Social Livelihood Science and Technology Program of Jiangsu Province (Grant No. MSZ2025025).

## Conflicts of Interest

The authors declare no conflicts of interest.

## Supporting Information

Additional supporting information can be found online in the Supporting Information section.

## Supporting information


**Supporting Information** The supporting figures in this study provide comprehensive external validation, TIME analysis, and potential therapeutic drug screening results to further support the core findings of the main manuscript. Supporting Figure 1 validates the differential expression and subcellular localization of CTNNB1 in pancreatic ductal adenocarcinoma (PDAC) and normal pancreatic tissues through multiple independent GEO cohorts and HPA‐based immunohistochemical staining. Supporting Figure 2 systematically explores the correlation between *CTNNB1* expression and tumor immune infiltration, and immune‐related gene signatures, as well as multiomics and genomic immune phenotypes in the TCGA‐PAAD cohort using multiple bioinformatic algorithms. Supporting Figure 3 conducts pan‐cancer drug susceptibility screening and multicohort validation, identifying potential *CTNNB1*‐targeted therapeutic compounds for PDAC based on multiple pharmacogenomic and CMap databases. Supporting Code S1 contains all annotated original R scripts together with example datasets, enabling full execution and reproducibility of all bioinformatic analyses. All Supporting data supplement and reinforce the reliability and scientific rigor of the conclusions regarding the prognostic value, immune regulatory role, and clinical therapeutic implication of *CTNNB1* in PDAC.

## Data Availability

The data that support the findings of this study are available from GDC (https://portal.gdc.cancer.gov/) and cBioPortal (https://www.cbioportal.org/) for TCGA‐PAAD. Additional public transcriptome datasets reanalyzed in this work were downloaded from the NCBI Gene Expression Omnibus (GEO) and ArrayExpress repositories. All corresponding accession numbers (GSE and E‐MTAB identifiers) are provided within the main text and figure legends. The core analysis code is provided as Supporting Information.

## References

[1] Lyu G. and Li D. , ZP3 Expression in Pancreatic Adenocarcinoma: Its Implications for the Prognosis and Therapy, Protein and Peptide Letters. (2025) 32, no. 2, 124–138, 10.2174/0109298665350171241204153202. 39791146

[2] Dwivedi M. , Sanyal S. , Singh S. , Dwivedi M. , and Sanyal S. , Target and Gene-Based Therapeutic Strategies Against Pancreatic Cancer: Current and Future Prospects, Current Gene Therapy. (2025) 25, no. 4, 417–432, 10.2174/0115665232320846240910055032. 39318213

[3] Zhou X. and Zhang T. , Mechanisms Underlying the Therapeutic Effects of Banzhilian-Baihuasheshecao for Treating Pancreatic Ductal Adenocarcinoma Based on Bioinformatics Strategy, Letters in Drug Design & Discovery. (2024) 21, no. 13, 2618–2643, 10.2174/0115701808258777230926092527.

[4] Zhao Z. and Liu W. , Pancreatic Cancer: A Review of Risk Factors, Diagnosis, and Treatment, Technology in Cancer Research and Treatment. (2020) 19, 10.1177/1533033820962117. PMC7768873 33357065

[5] Tian G. , Liu C. , Che C. et al., Identifying ARRB2 as a Prognostic Biomarker and Key Player in the Tumor Microenvironment of Pancreatic Cancer Through scPagwas Methodology, Current Gene Therapy. (2026) 26, no. 1, 203–222, 10.2174/0115665232427614250904061700. 40926605

[6] Fang J. , Huang J. , Zhang J. , Chen L. , and Deng J. , Comprehensive Analysis of Tertiary Lymphoid Structures in Pancreatic Cancer: Molecular Characteristics and Prognostic Implications, Current Proteomics. (2024) 21, no. 4, 230–250, 10.2174/0115701646317271240821071544.

[7] Zhuang W. , Ye T. , Wang W. , Song W. , and Tan T. , CTNNB1 in Neurodevelopmental Disorders, Frontiers in Psychiatry. (2023) 14, 10.3389/fpsyt.2023.1143328. PMC10061110 37009120

[8] Gao C. , Wang Y. , Broaddus R. , Sun L. , Xue F. , and Zhang W. , Exon 3 Mutations of CTNNB1 Drive Tumorigenesis: A Review, Oncotarget. (2017) 9, no. 4, 5492–5508, 10.18632/oncotarget.23695. 29435196 PMC5797067

[9] Saegusa M. , Hashimura M. , Yoshida T. , and Okayasu I. , Beta-Catenin Mutations and Aberrant Nuclear Expression During Endometrial Tumorigenesis, British Journal of Cancer. (2001) 84, no. 2, 209–217, 10.1054/bjoc.2000.1581. 11161379 PMC2363713

[10] Wen J. , Min X. , Shen M. et al., ACLY Facilitates Colon Cancer Cell Metastasis by CTNNB1, Journal of Experimental & Clinical Cancer Research. (2019) 38, no. 1, 10.1186/s13046-019-1391-9. PMC6740040 31511060

[11] Cai N. , Cheng K. , Ma Y. et al., Targeting MMP9 in CTNNB1 Mutant Hepatocellular Carcinoma Restores CD8+ T Cell-Mediated Antitumour Immunity and Improves anti-PD-1 Efficacy, Gut. (2024) 73, no. 6, 985–999, 10.1136/gutjnl-2023-331342. 38123979 PMC11103337

[12] Guichard C. , Amaddeo G. , Imbeaud S. et al., Integrated Analysis of Somatic Mutations and Focal Copy-Number Changes Identifies Key Genes and Pathways in Hepatocellular Carcinoma, Nature Genetics. (2012) 44, no. 6, 694–698, 10.1038/ng.2256. 22561517 PMC3819251

[13] Rebouissou S. , Franconi A. , Calderaro J. et al., Genotype-Phenotype Correlation of CTNNB1 Mutations Reveals Different ß-Catenin Activity Associated With Liver Tumor Progression, Hepatology. (2016) 64, no. 6, 2047–2061, 10.1002/hep.28638. 27177928

[14] Schwarz-Romond T. , Fiedler M. , Shibata N. et al., The DIX Domain of Dishevelled Confers Wnt Signaling by Dynamic Polymerization, Nature Structural & Molecular Biology. (2007) 14, no. 6, 484–492, 10.1038/nsmb1247. 17529994

[15] Vlad A. , Röhrs S. , Klein-Hitpass L. , and Müller O. , The First Five Years of the Wnt Targetome, Cellular Signalling. (2008) 20, no. 5, 795–802, 10.1016/j.cellsig.2007.10.031. 18160255

[16] Nie Q. , Peng W. W. , Wang Y. , Zhong L. , Zhang X. , and Zeng L. , β-Catenin Correlates With the Progression of Colon Cancers and Berberine Inhibits the Proliferation of Colon Cancer Cells by Regulating the β-Catenin Signaling Pathway, Gene. (2022) 818, 10.1016/j.gene.2022.146207. 35063579

[17] Wang S. , Tian Y. , Wu D. et al., Genetic Variation of CTNNB1 Gene is Associated With Susceptibility and Prognosis of Gastric Cancer in a Chinese Population, Mutagenesis. (2012) 27, no. 6, 623–630, 10.1093/mutage/ges027. 22848100

[18] Cui J. , Li P. , Liu X. , Hu H. , and Wei W. , Abnormal Expression of the Notch and Wnt/β-Catenin Signaling Pathways in Stem-Like ALDHhiCD44+ Cells Correlates Highly With Ki-67 Expression in Breast Cancer, Oncology Letters. (2015) 9, no. 4, 1600–1606, 10.3892/ol.2015.2942. 25789008 PMC4356390

[19] Francis J. C. , Thomsen M. K. , Taketo M. M. , and Swain A. , β-Catenin is Required for Prostate Development and Cooperates With Pten Loss to Drive Invasive Carcinoma, PLoS Genetics. (2013) 9, no. 1, 10.1371/journal.pgen.1003180. PMC3536663 23300485

[20] Kypta R. M. and Waxman J. , Wnt/β-Catenin Signalling in Prostate Cancer, Nature Reviews Urology. (2012) 9, no. 8, 418–428, 10.1038/nrurol.2012.116. 22710668

[21] Song P. , Gao Z. , Bao Y. et al., Wnt/β-catenin Signaling Pathway in Carcinogenesis and Cancer Therapy, Journal of Hematology & Oncology. (2024) 17, no. 1, 10.1186/s13045-024-01563-4. PMC11184729 38886806

[22] Barzegar Behrooz A. , Talaie Z. , Jusheghani F. , Łos M. J. , Klonisch T. , and Ghavami S. , Wnt and PI3K/Akt/mTOR Survival Pathways as Therapeutic Targets in Glioblastoma, International Journal of Molecular Sciences. (2022) 23, no. 3, 10.3390/ijms23031353. PMC8836096 35163279

[23] Nager M. , Bhardwaj D. , Cantí C. , Medina L. , Nogués P. , and Herreros J. , β-Catenin Signalling in Glioblastoma Multiforme and Glioma-Initiating Cells, Chemother Res Pract. (2012) 2012, 192362–192367, 10.1155/2012/192362. 22400111 PMC3286890

[24] Iwata S. , Kitazawa R. , Kitazawa S. , and Hato N. , Glomangiopericytoma With CTNNB1 Mutation, BMJ Case Reports. (2023) 16, no. 9, 10.1136/bcr-2023-256787. PMC10510870 37723085

[25] Li H. and Durbin R. , Fast and Accurate Long-Read Alignment With Burrows-Wheeler Transform, Bioinformatics. (2010) 26, no. 5, 589–595, 10.1093/bioinformatics/btp698. 20080505 PMC2828108

[26] McKenna A. , Hanna M. , Banks E. et al., The Genome Analysis Toolkit: A MapReduce Framework for Analyzing Next-Generation DNA Sequencing Data, Genome Research. (2010) 20, no. 9, 1297–1303, 10.1101/gr.107524.110. 20644199 PMC2928508

[27] Koboldt D. C. , Zhang Q. , Larson D. E. et al., Varscan 2: Somatic Mutation and Copy Number Alteration Discovery in Cancer by Exome Sequencing, Genome Research. (2012) 22, no. 3, 568–576, 10.1101/gr.129684.111. 22300766 PMC3290792

[28] Wang K. , Li M. , and Hakonarson H. , ANNOVAR: Functional Annotation of Genetic Variants From High-Throughput Sequencing Data, Nucleic Acids Research. (2010) 38, no. 16, 10.1093/nar/gkq603. PMC2938201 20601685

[29] Newman A. M. , Bratman S. V. , Stehr H. et al., FACTERA: A Practical Method for the Discovery of Genomic Rearrangements at Breakpoint Resolution, Bioinformatics. (2014) 30, no. 23, 3390–3393, 10.1093/bioinformatics/btu549. 25143292 PMC4296148

[30] Beroukhim R. , Mermel C. H. , Porter D. et al., The Landscape of Somatic Copy-Number Alteration Across Human Cancers, Nature. (2010) 463, no. 7283, 899–905, 10.1038/nature08822. 20164920 PMC2826709

[31] Han Y. , Wang Y. , Dong X. et al., TISCH2: Expanded Datasets and New Tools for Single-Cell Transcriptome Analyses of the Tumor Microenvironment, Nucleic Acids Research. (2023) 51, no. D1, D1425–D1431, 10.1093/nar/gkac959. 36321662 PMC9825603

[32] Shi J. , Wei X. , Xun Z. et al., The Web-Based Portal SpatialTME Integrates Histological Images With Single-Cell and Spatial Transcriptomics to Explore the Tumor Microenvironment, Cancer Research. (2024) 84, no. 8, 1210–1220, 10.1158/0008-5472.CAN-23-2650. 38315776

[33] Xun Z. , Ding X. , Zhang Y. et al., Reconstruction of the Tumor Spatial Microenvironment Along the Malignant-Boundary-Nonmalignant Axis, Nature Communications. (2023) 14, no. 1, 10.1038/s41467-023-36560-7. PMC9941488 36806082

[34] Thorsson V. , Gibbs D. L. , Brown S. D. et al., The Immune Landscape of Cancer, Immunity. (2018) 48, no. 4, 812–830.e14, 10.1016/j.immuni.2018.03.023. 29628290 PMC5982584

[35] Li T. , Fan J. , Wang B. et al., TIMER: A Web Server for Comprehensive Analysis of Tumor-Infiltrating Immune Cells, Cancer Research. (2017) 77, no. 21, e108–e110, 10.1158/0008-5472.CAN-17-0307. 29092952 PMC6042652

[36] Racle J. , de Jonge K. , Baumgaertner P. , Speiser D. E. , and Gfeller D. , Simultaneous Enumeration of Cancer and Immune Cell Types From Bulk Tumor Gene Expression Data, eLife. (2017) 6, 10.7554/eLife.26476. PMC5718706 29130882

[37] Becht E. , Giraldo N. A. , Lacroix L. et al., Estimating the Population Abundance of Tissue-Infiltrating Immune and Stromal Cell Populations Using Gene Expression, Genome Biology. (2016) 17, no. 1, 10.1186/s13059-016-1070-5. PMC5134277 27908289

[38] Aran D. , Hu Z. , and Butte A. J. , xCell: Digitally Portraying the Tissue Cellular Heterogeneity Landscape, Genome Biology. (2017) 18, no. 1, 10.1186/s13059-017-1349-1. PMC5688663 29141660

[39] Newman A. M. , Liu C. L. , Green M. R. et al., Robust Enumeration of Cell Subsets From Tissue Expression Profiles, Nature Methods. (2015) 12, no. 5, 453–457, 10.1038/nmeth.3337. 25822800 PMC4739640

[40] Xu L. , Deng C. , Pang B. et al., TIP: A Web Server for Resolving Tumor Immunophenotype Profiling, Cancer Research. (2018) 78, no. 23, 6575–6580, 10.1158/0008-5472.CAN-18-0689. 30154154

[41] Lapuente-Santana Ó. , van Genderen M. , Hilbers P. A. J. , Finotello F. , and Eduati F. , Interpretable Systems Biomarkers Predict Response to Immune-Checkpoint Inhibitors, Patterns (N Y). (2021) 2, no. 8, 10.1016/j.patter.2021.100293. PMC8369166 34430923

[42] Jiang P. , Gu S. , Pan D. et al., Signatures of T Cell Dysfunction and Exclusion Predict Cancer Immunotherapy Response, Nature Medicine. (2018) 24, no. 10, 1550–1558, 10.1038/s41591-018-0136-1. PMC6487502 30127393

[43] Maeser D. , Gruener R. F. , and Huang R. S. , oncoPredict: An R Package for Predicting In Vivo or Cancer Patient Drug Response and Biomarkers From Cell Line Screening Data, Briefings in Bioinformatics. (2021) 22, no. 6, 10.1093/bib/bbab260. PMC8574972 34260682

[44] Yang C. , Zhang H. , Chen M. et al., A Survey of Optimal Strategy for Signature-Based Drug Repositioning and an Application to Liver Cancer, eLife. (2022) 11, 10.7554/eLife.71880. PMC8893721 35191375

[45] van der Wal T. and van Amerongen R. , Walking the Tight Wire Between Cell Adhesion and WNT Signalling: A Balancing Act for β-Catenin, Open Biology. (2020) 10, no. 12, 10.1098/rsob.200267. PMC7776579 33292105

[46] Xue C. , Chu Q. , Shi Q. , Zeng Y. , Lu J. , and Li L. , Wnt Signaling Pathways in Biology and Disease: Mechanisms and Therapeutic Advances, Signal Transduction and Targeted Therapy. (2025) 10, no. 1, 10.1038/s41392-025-02142-w. PMC11968978 40180907

[47] Yang Y. , Ding Y. , Gong Y. et al., The Genetic Landscape of Pancreatic Head Ductal Adenocarcinoma in China and Prognosis Stratification, BMC Cancer. (2022) 22, no. 1, 10.1186/s12885-022-09279-9. PMC8855595 35180847

[48] Ribas A. and Wolchok J. D. , Cancer Immunotherapy Using Checkpoint Blockade, Science. (2018) 359, no. 6382, 1350–1355, 10.1126/science.aar4060. 29567705 PMC7391259

[49] Greten T. F. , Lai C. W. , Li G. , and Staveley-O’Carroll K. F. , Targeted and Immune-Based Therapies for Hepatocellular Carcinoma, Gastroenterology. (2019) 156, no. 2, 510–524, 10.1053/j.gastro.2018.09.051. 30287171 PMC6340758

[50] Zuluaga Gómez L. M. , Caballero Mojica S. C. , Vélez Rengifo G. J. , Bravo Acosta J. D. , and Montoya Villada J. H. , CTNNB1 Gene Mutation Associated With Neurodevelopmental Disorder, Microcephaly, and Persistence of Bilateral Hyperplastic Primary Vitreous: A Case Report and Literature Review, Archivos de la Sociedad Espanola de Oftalmologia. (2022) 97, no. 1, 44–47, 10.1016/j.oftale.2020.11.018. 35027145

[51] Kharbanda M. , Pilz D. T. , Tomkins S. et al., Clinical Features Associated with CTNNB1 de Novo Loss of Function Mutations in Ten Individuals, European Journal of Medical Genetics. (2017) 60, no. 2, 130–135, 10.1016/j.ejmg.2016.11.008. 27915094 PMC6070129

[52] Drenser K. A. , Wnt Signaling Pathway in Retinal Vascularization, Eye and Brain. (2016) 8, 141–146, 10.2147/EB.S94452. 28539809 PMC5398754

[53] Sekine S. , Shibata T. , Kokubu A. et al., Craniopharyngiomas of Adamantinomatous Type Harbor Beta-Catenin Gene Mutations, American Journal of Pathology. (2002) 161, no. 6, 1997–2001, 10.1016/s0002-9440(10)64477-x. 12466115 PMC1850925

[54] Reyes M. , Taghvaei M. , Yu S. et al., Targeted Therapy in the Management of Modern Craniopharyngiomas, Frontiers in Bioscience (Landmark Edition). (2022) 27, no. 4, 10.31083/j.fbl2704136. 35468695

[55] Ye G. , Yang Q. , Lei X. et al., Nuclear MYH9-Induced CTNNB1 Transcription, Targeted by Staurosporin, Promotes Gastric Cancer Cell Anoikis Resistance and Metastasis, Theranostics. (2020) 10, no. 17, 7545–7560, 10.7150/thno.46001. 32685004 PMC7359096

[56] Moroney M. R. , Woodruff E. , Qamar L. et al., Inhibiting Wnt/Beta-Catenin in CTNNB1-Mutated Endometrial Cancer, Molecular Carcinogenesis. (2021) 60, no. 8, 511–523, 10.1002/mc.23308. 34038589

[57] Wu H. , Lu X. X. , Wang J. R. et al., TRAF6 Inhibits Colorectal Cancer Metastasis Through Regulating Selective Autophagic CTNNB1/β-Catenin Degradation and Is Targeted for GSK3B/GSK3β-Mediated Phosphorylation and Degradation, Autophagy. (2019) 15, no. 9, 1506–1522, 10.1080/15548627.2019.1586250. 30806153 PMC6693460

[58] Chronopoulos A. , Robinson B. , Sarper M. et al., ATRA Mechanically Reprograms Pancreatic Stellate Cells to Suppress Matrix Remodelling and Inhibit Cancer Cell Invasion, Nature Communications. (2016) 7, no. 1, 10.1038/ncomms12630. PMC5023948 27600527

[59] Li Z. , Cao H. , and Xu W. , The Role of the Tumor Microenvironment (TME) and Relevant Novel Biomarkers in Oncogenesis, Frontiers in Genetics, Cancer Genetics and Oncogenomics Research Topic, https://www.frontiersin.org/research-topics/37587/the-role-of-the-tumor-microenvironment-tme-and-relevant-novel-biomarkers-in-oncogenesis.

[60] Zhang Y. and Zhang Z. , The History and Advances in Cancer Immunotherapy: Understanding the Characteristics of Tumor-Infiltrating Immune Cells and Their Therapeutic Implications, Cellular and Molecular Immunology. (2020) 17, no. 8, 807–821, 10.1038/s41423-020-0488-6. 32612154 PMC7395159

[61] Almatroodi S. A. , McDonald C. F. , Darby I. A. , and Pouniotis D. S. , Characterization of M1/M2 Tumour-Associated Macrophages (TAMs) and Th1/Th2 Cytokine Profiles in Patients With NSCLC, Cancer Microenviron. (2016) 9, no. 1, 1–11, 10.1007/s12307-015-0174-x. 26319408 PMC4842181

[62] Vázquez-Bellón N. , Martínez-Bosch N. , García de Frutos P. , and Navarro P. , Hallmarks of Pancreatic Cancer: Spotlight on TAM Receptors, EBioMedicine. (2024) 107, 10.1016/j.ebiom.2024.105278. PMC11367522 39137571

[63] Lv B. , Wang Y. , Ma D. et al., Immunotherapy: Reshape the Tumor Immune Microenvironment, Frontiers in Immunology. (2022) 13, 10.3389/fimmu.2022.844142. PMC9299092 35874717

[64] Gajewski T. F. , Schreiber H. , and Fu Y. X. , Innate and Adaptive Immune Cells in the Tumor Microenvironment, Nature Immunology. (2013) 14, no. 10, 1014–1022, 10.1038/ni.2703. 24048123 PMC4118725

[65] Chen J. , Mei Z. , Huang B. et al., IL-6/YAP1/β-Catenin Signaling is Involved in Intervertebral Disc Degeneration, Journal of Cellular Physiology. (2019) 234, no. 5, 5964–5971, 10.1002/jcp.27065. 30511395 PMC6686169

[66] Pardoll D. M. , The Blockade of Immune Checkpoints in Cancer Immunotherapy, Nature Reviews Cancer. (2012) 12, no. 4, 252–264, 10.1038/nrc3239. 22437870 PMC4856023

[67] Chen L. and Flies D. B. , Molecular Mechanisms of T Cell Co-Stimulation and Co-Inhibition, Nature Reviews Immunology. (2013) 13, no. 4, 227–242, 10.1038/nri3405. PMC3786574 23470321

[68] Ikeda H. , Old L. J. , and Schreiber R. D. , The Roles of IFN Gamma in Protection Against Tumor Development and Cancer Immunoediting, Cytokine & Growth Factor Reviews. (2002) 13, no. 2, 95–109, 10.1016/s1359-6101(01)00038-7. 11900986

[69] Bald T. , Landsberg J. , Lopez-Ramos D. et al., Immune Cell-Poor Melanomas Benefit From PD-1 Blockade After Targeted Type I IFN Activation, Cancer Discovery. (2014) 4, no. 6, 674–687, 10.1158/2159-8290.CD-13-0458. 24589924

[70] Liang S. C. , Latchman Y. E. , Buhlmann J. E. et al., Regulation of PD-1, PD-L1, and PD-L2 Expression During Normal and Autoimmune Responses, European Journal of Immunology. (2003) 33, no. 10, 2706–2716, 10.1002/eji.200324228. 14515254

[71] Abiko K. , Matsumura N. , Hamanishi J. et al., IFN-γ From Lymphocytes Induces PD-L1 Expression and Promotes Progression of Ovarian Cancer, British Journal of Cancer. (2015) 112, no. 9, 1501–1509, 10.1038/bjc.2015.101. 25867264 PMC4453666

[72] Bellucci R. , Martin A. , Bommarito D. et al., Interferon-γ-Induced Activation of JAK1 and JAK2 Suppresses Tumor Cell Susceptibility to NK Cells Through Upregulation of PD-L1 Expression, Oncoimmunology. (2015) 4, no. 6, 10.1080/2162402X.2015.1008824. PMC4485824 26155422

[73] Spranger S. , Spaapen R. M. , Zha Y. et al., Up-Regulation of PD-L1, IDO, and T(Regs) in the Melanoma Tumor Microenvironment is Driven by CD8(+) T Cells, Science Translational Medicine. (2013) 5, no. 200, 10.1126/scitranslmed.3006504. PMC4136707 23986400

[74] Ayers M. , Lunceford J. , Nebozhyn M. et al., IFN-γ-Related mRNA Profile Predicts Clinical Response to PD-1 Blockade, Journal of Clinical Investigation. (2017) 127, no. 8, 2930–2940, 10.1172/JCI91190. 28650338 PMC5531419

[75] Galon J. and Bruni D. , Tumor Immunology and Tumor Evolution: Intertwined Histories, Immunity. (2020) 52, no. 1, 55–81, 10.1016/j.immuni.2019.12.018. 31940273

[76] Zhang A. , Fan T. , Liu Y. , Yu G. , Li C. , and Jiang Z. , Regulatory T Cells in Immune Checkpoint Blockade Antitumor Therapy, Molecular Cancer. (2024) 23, no. 1, 10.1186/s12943-024-02156-y. PMC11545879 39516941

[77] Zhang J. , Li R. , and Huang S. , The Immunoregulation Effect of Tumor Microenvironment in Pancreatic Ductal Adenocarcinoma, Frontiers in Oncology. (2022) 12, 10.3389/fonc.2022.951019. PMC9365986 35965504

[78] Clevers H. and Nusse R. , Wnt/β-Catenin Signaling and Disease, Cell. (2012) 149, no. 6, 1192–1205, 10.1016/j.cell.2012.05.012. 22682243

[79] Behrens J. , von Kries J. P. , Kühl M. et al., Functional Interaction of Beta-Catenin With the Transcription Factor LEF-1, Nature. (1996) 382, no. 6592, 638–642, 10.1038/382638a0. 8757136

[80] Peifer M. , Pai L. M. , and Casey M. , Phosphorylation of the Drosophila Adherens Junction Protein Armadillo: Roles for Wingless Signal and Zeste-White 3 Kinase, Developmental Biology. (1994) 166, no. 2, 543–556, 10.1006/dbio.1994.1336. 7529201

[81] Rubinfeld B. , Albert I. , Porfiri E. , Fiol C. , Munemitsu S. , and Polakis P. , Binding of GSK3beta to the APC-Beta-Catenin Complex and Regulation of Complex Assembly, Science. (1996) 272, no. 5264, 1023–1026, 10.1126/science.272.5264.1023. 8638126

[82] Yost C. , Torres M. , Miller J. R. , Huang E. , Kimelman D. , and Moon R. T. , The Axis-Inducing Activity, Stability, and Subcellular Distribution of Beta-Catenin is Regulated in Xenopus Embryos by Glycogen Synthase Kinase 3, Genes & Development. (1996) 10, no. 12, 1443–1454, 10.1101/gad.10.12.1443. 8666229

[83] Spranger S. , Bao R. , and Gajewski T. F. , Melanoma-Intrinsic β-Catenin Signalling Prevents Anti-Tumour Immunity, Nature. (2015) 523, no. 7559, 231–235, 10.1038/nature14404. 25970248

[84] Fu C. and Jiang A. , β-Catenin-Mediated Inhibition of Cross-Priming: A New Mechanism for Tumors to Evade Immunosurveillance, Oncoimmunology. (2013) 2, no. 12, 10.4161/onci.26920. PMC3925153 24567866

[85] Zhu Y. H. , Zheng J. H. , Jia Q. Y. et al., Immunosuppression, Immune Escape, and Immunotherapy in Pancreatic Cancer: Focused on the Tumor Microenvironment, Cellular Oncology. (2023) 46, no. 1, 17–48, 10.1007/s13402-022-00741-1. 36367669 PMC12974690

[86] Du W. , Menjivar R. E. , Donahue K. L. et al., WNT Signaling in the Tumor Microenvironment Promotes Immunosuppression in Murine Pancreatic Cancer, Journal of Experimental Medicine. (2023) 220, no. 1, 10.1084/jem.20220503. PMC9577101 36239683

[87] Soukup O. , Tobin G. , Kumar U. K. , Jun D. , Fusek J. , and Kuca K. , Characterization of the Anticholinergic Properties of Obidoxime; Functional Examinations of the Rat Atria and the Urinary Bladder, Toxicology Mechanisms and Methods. (2010) 20, no. 7, 428–433, 10.3109/15376516.2010.497974. 20602545

[88] Lucianò A. M. , Perciballi E. , Fiore M. , Del Bufalo D. , and Tata A. M. , The Combination of the M2 Muscarinic Receptor Agonist and Chemotherapy Affects Drug Resistance in Neuroblastoma Cells, International Journal of Molecular Sciences. (2020) 21, no. 22, 10.3390/ijms21228433. PMC7697391 33182656

[89] Chen X. , Gong L. , Ou R. et al., Sequential Combination Therapy of Ovarian Cancer With Cisplatin and γ-Secretase Inhibitor MK-0752, Gynecologic Oncology. (2016) 140, no. 3, 537–544, 10.1016/j.ygyno.2015.12.011. 26704638

[90] Cook N. , Basu B. , Smith D. M. et al., A Phase I Trial of the γ-Secretase Inhibitor MK-0752 in Combination With Gemcitabine in Patients With Pancreatic Ductal Adenocarcinoma, British Journal of Cancer. (2018) 118, no. 6, 793–801, 10.1038/bjc.2017.495. 29438372 PMC5877439

[91] Huang T. and He W. Y. , Construction and Validation of a Novel Prognostic Signature of Idiopathic Pulmonary Fibrosis by Identifying Subtypes Based on Genes Related to 7-Methylguanosine Modification, Frontiers in Genetics. (2022) 13, 10.3389/fgene.2022.890530. PMC9218869 35754799

[92] Rupniewska E. , Roy R. , Mauri F. A. et al., Targeting Autophagy Sensitises Lung Cancer Cells to Src Family Kinase Inhibitors, Oncotarget. (2018) 9, no. 44, 27346–27362, 10.18632/oncotarget.25213. 29937990 PMC6007948

