# Supplementary material for: Prognostic and Immunological Value of CTNNB1 in Pancreatic Adenocarcinoma: A Comprehensive NGS and Multicomponent Analysis
Source: Genet Res (Camb). 2026 Sep 9;2026:8700258. doi: 10.1155/genr/8700258 (PMC13555821; doi:10.1155/genr/8700258)
Supplement: Supplementary file 1 [file GENR-2026-8700258-s001.zip › Supplementary_Code_S1.pdf]

```r
# ==============================================================================
# merge_by_rownames() — Inner-join two tables on shared row names
#
# Method : Align two matrices or data.frames by their row names, keep only the
#          rows present in both (the intersection), reorder both to that common
#          set, then column-bind them into one table. Column names shared by the
#          two inputs are disambiguated with suffixes so nothing is silently
#          overwritten. This is the matrix analogue of merge(by = "row.names")
#          but it preserves matrix type and controls row order.
# Input  : x, y   matrices or data.frames sharing some row names (e.g. two omics
#                 blocks measured on the same features, or features x samples).
# Output : a combined matrix (when both inputs are matrices) or data.frame, with
#          the shared row names and the columns of x followed by those of y.
# Depends: base R only.
# ==============================================================================

#' Inner-join two tables on shared row names
#'
#' @param x,y matrices or data.frames with (some) shared row names.
#' @param suffixes length-2 character vector appended to otherwise-identical
#'   column names from \code{x} and \code{y} (default c(".x", ".y")).
#' @return the row-aligned column-bind of \code{x} and \code{y} over their common
#'   row names; a matrix when both inputs are matrices, otherwise a data.frame.
merge_by_rownames <- function(x, y, suffixes = c(".x", ".y")) {
  if (is.null(rownames(x)) || is.null(rownames(y)))
    stop("both `x` and `y` must have row names.")
  common <- intersect(rownames(x), rownames(y))
  if (length(common) == 0L) stop("no shared row names between `x` and `y`.")

  x2 <- x[common, , drop = FALSE]
  y2 <- y[common, , drop = FALSE]

  dup <- intersect(colnames(x2), colnames(y2))
  if (length(dup)) {
    ix <- colnames(x2) %in% dup; iy <- colnames(y2) %in% dup
    colnames(x2)[ix] <- paste0(colnames(x2)[ix], suffixes[1L])
    colnames(y2)[iy] <- paste0(colnames(y2)[iy], suffixes[2L])
  }

  both_mat <- is.matrix(x) && is.matrix(y)
  out <- if (both_mat) cbind(x2, y2) else data.frame(x2, y2, check.names = FALSE)
  rownames(out) <- common
  out
}

# ---- Example (simulated data; select this block and run) --------------------
if (FALSE) {
  set.seed(1)
  x <- matrix(rnorm(20), nrow = 5,
              dimnames = list(paste0("Gene", 1:5), paste0("mRNA", 1:4)))
  y <- matrix(rnorm(18), nrow = 6,
              dimnames = list(paste0("Gene", 2:7), paste0("Prot", 1:3)))
  merge_by_rownames(x, y)     # keeps Gene2..Gene5, present in both
}
# ==============================================================================
# parallel_apply() — lapply that uses forked parallelism when it is safe
#
```

```r
# Method : A thin wrapper over lapply that dispatches to parallel::mclapply
#          (fork-based) when more than one core is requested AND the platform
#          supports forking (Unix / macOS). On Windows, or when n_cores == 1, it
#          falls back to a plain serial lapply, so calling code stays identical
#          across platforms. The requested core count is capped at the number the
#          machine reports as available.
# Input  : X     a vector or list to iterate over.
#          FUN   function applied to each element of X.
# Output : a list of length(X), identical in structure to lapply(X, FUN, ...).
# Depends: base R only (the parallel package ships with base R).
# ============================================================================

#' lapply with optional forked parallelism
#'
#' @param X a vector or list to iterate over.
#' @param FUN function applied to each element of \code{X}.
#' @param ... further arguments passed to \code{FUN}.
#' @param n_cores number of worker cores (default 2). Values > 1 use
#'   \code{parallel::mclapply} on Unix/macOS; Windows and \code{n_cores == 1}
#'   run serially.
#' @return a list of results, one per element of \code{X}.
parallel_apply <- function(X, FUN, ..., n_cores = 2L) {
  n_cores <- max(1L, as.integer(n_cores))
  avail   <- parallel::detectCores()
  if (is.na(avail)) avail <- 1L
  n_cores <- min(n_cores, max(1L, avail))

  can_fork <- .Platform$OS.type != "windows"
  if (n_cores > 1L && can_fork)
    parallel::mclapply(X, FUN, ..., mc.cores = n_cores)
  else
    lapply(X, FUN, ...)
}

# ---- Example (simulated data; select this block and run) --------------------
if (FALSE) {
  res <- parallel_apply(1:6, function(i) i^2, n_cores = 2)
  unlist(res)                       # 1 4 9 16 25 36
}
# ============================================================================
# collapse_duplicate_genes()  –  Collapse duplicate feature rows to one per gene
#
# Method : When several rows map to the same gene symbol (e.g. multiple probes),
#          collapse them to a single row per gene. FUN selects the rule:
#            "mean" / "median"  –  summarize each sample across the duplicate rows;
#            "max"              –  keep, per gene, the row with the highest mean;
#            "maxvar"           –  keep, per gene, the row with the highest variance
#                                  (the most informative probe, a common array rule).
#          Rows whose gene id is missing or blank are dropped. limma::avereps is a
#          well-known alternative for the mean rule; this implementation is pure
#          base R and needs no extra package.
# Input  : x       numeric matrix (rows = probes/features, columns = samples).
#          genes   character vector of length nrow(x) giving each row's gene id
#                  (defaults to rownames(x)).
# Output : a numeric matrix with unique gene rownames, one row per gene.
# Depends: base R only.
# ============================================================================
```

```r
#' Collapse duplicate gene rows to one row per gene
#'
#' @param x numeric matrix (features x samples).
#' @param genes character vector of gene ids, length \code{nrow(x)}
#'      (default \code{rownames(x)}).
#' @param FUN collapse rule: one of "mean", "median", "max", "maxvar".
#' @return a numeric matrix with one row per unique gene.
collapse_duplicate_genes <- function(x, genes = rownames(x),
                                     FUN = c("mean", "median", "max", "maxvar")) {
  FUN <- match.arg(FUN)
  x <- as.matrix(x)
  if (is.null(genes) || length(genes) != nrow(x))
    stop("`genes` must be a vector of length nrow(x).")

  genes <- as.character(genes)
  keep   <- !is.na(genes) & nzchar(trimws(genes))
  x <- x[keep, , drop = FALSE]; genes <- genes[keep]

  uniq <- unique(genes)
  out   <- matrix(NA_real_, nrow = length(uniq), ncol = ncol(x),
                  dimnames = list(uniq, colnames(x)))

  for (g in uniq) {
    rows <- which(genes == g)
    if (length(rows) == 1L) { out[g, ] <- x[rows, ]; next }
    block <- x[rows, , drop = FALSE]
    out[g, ] <- switch(FUN,
      mean     = colMeans(block, na.rm = TRUE),
      median = apply(block, 2L, stats::median, na.rm = TRUE),
      max       = block[which.max(rowMeans(block, na.rm = TRUE)), ],
      maxvar = block[which.max(apply(block, 1L, stats::var, na.rm = TRUE)), ])
  }
  out
}

# ---- Example (simulated data; select this block and run) --------------------
if (FALSE) {
  set.seed(1)
  x <- matrix(rnorm(24), nrow = 6, dimnames = list(NULL, paste0("S", 1:4)))
  genes <- c("TP53", "TP53", "EGFR", "EGFR", "EGFR", "MYC")
  collapse_duplicate_genes(x, genes, FUN = "maxvar")      # 3 rows: TP53/EGFR/MYC
}

# ============================================================================
# filter_high_na()  —  Drop rows and/or columns with too many missing values
#
# Method : Compute the fraction of NA per row and/or per column and drop those
#                exceeding a cutoff. When `margin` is "both", columns are filtered
#                first, then rows are re-evaluated on the surviving columns, so the row
#                decision reflects the data that actually remain. Useful before
#                imputation or model fitting to remove features/samples too sparse to
#                estimate reliably.
# Input    : x    a matrix or data.frame that may contain NA.
# Output : the row/column subset of x meeting the NA-fraction cutoff.
# Depends: base R only.
# ============================================================================
```

```r
#' Drop rows/columns whose NA fraction exceeds a cutoff
#'
#' @param x matrix or data.frame.
#' @param cutoff maximum allowed fraction of NA (default 0.2); a row/column with
#'     a higher NA fraction is dropped.
#' @param margin "both" (default), "row", or "column".
#' @return the filtered object.
filter_high_na <- function(x, cutoff = 0.2, margin = c("both", "row", "column")) {
  margin   <- match.arg(margin)
  na_frac <- function(v) mean(is.na(v))

  if (margin %in% c("both", "column")) {
    keep_col <- apply(x, 2L, na_frac) <= cutoff
    x <- x[, keep_col, drop = FALSE]
  }
  if (margin %in% c("both", "row")) {
    keep_row <- apply(x, 1L, na_frac) <= cutoff
    x <- x[keep_row, , drop = FALSE]
  }
  x
}

# ---- Example (simulated data; select this block and run) -------------------
if (FALSE) {
  set.seed(1)
  x <- matrix(rnorm(50), nrow = 10,
              dimnames = list(paste0("G", 1:10), paste0("S", 1:5)))
  x[1, 1:4] <- NA                         # row that is mostly NA
  x[, 5]    <- NA; x[6, 5] <- 1           # column that is mostly NA
  dim(filter_high_na(x, cutoff = 0.5, margin = "both"))     # 9 x 4
}
# ============================================================================
# filter_low_expression()  —  Drop weakly expressed features
#
# Method : Keep a feature (row) only if it exceeds an expression threshold in at
#          least a minimum number of samples. `min_samples` is read as an absolute
#          sample count when >= 1, or as a fraction of samples when in (0, 1).
#          This removes features that are essentially unexpressed across the
#          cohort before differential or correlation analysis, reducing multiple-
#          testing burden and unstable low-signal estimates. NAs never count as
#          passing.
# Input   : x   numeric matrix (features x samples).
# Output : the row-subset of x whose features pass the expression filter.
# Depends: base R only.
# ============================================================================

#' Drop features expressed below a threshold in too few samples
#'
#' @param x numeric matrix (features x samples).
#' @param threshold expression value a sample must exceed to "count"
#'     (default 1, i.e. > 1 on a log2(TPM+1)-like scale).
#' @param min_samples minimum number of samples (>= 1) or fraction (in (0,1))
#'     that must exceed \code{threshold} (default 0.2 = 20% of samples).
#' @return the filtered matrix (same columns, fewer or equal rows).
filter_low_expression <- function(x, threshold = 1, min_samples = 0.2) {
  x <- as.matrix(x)
```

```
  n <- ncol(x)
  need <- if (min_samples < 1) ceiling(min_samples * n) else as.numeric(min_samples)
  need <- max(1, need)

  n_pass <- rowSums(x > threshold, na.rm = TRUE)
  x[n_pass >= need, , drop = FALSE]
}

# ---- Example (simulated data; select this block and run) --------------------
if (FALSE) {
  set.seed(1)
  x <- rbind(matrix(rpois(40, 5), nrow = 8),          # expressed features
             matrix(0,    nrow = 2, ncol = 5))         # silent features
  rownames(x) <- paste0("Gene", 1:10)
  dim(filter_low_expression(x, threshold = 0, min_samples = 0.5))    # 8 rows
}
# ==============================================================================
# normalize_log2()  —  log2(x + pseudocount) transform
#
# Method : Apply a log2 transform after adding a pseudocount, the standard
#          variance-stabilizing step for non-negative expression measures. The
#          canonical use is log2(TPM + 1) (pseudocount = 1); a smaller pseudocount
#          (e.g. 0.5) shifts the trade-off at low values. Negative inputs are
#          rejected because log2 of a negative number is undefined  —  data that
#          are already on a log scale should not be passed here.
# Input    : x    numeric matrix or vector of non-negative expression values
#               (e.g. TPM, FPKM, or normalized counts).
# Output : a numeric object the same shape as x, on the log2 scale.
# Depends: base R only.
# ==============================================================================

#' log2(x + pseudocount) transform
#'
#' @param x numeric matrix or vector of non-negative values.
#' @param pseudocount value added before the log (default 1); must be > 0.
#' @return \code{log2(x + pseudocount)}, same shape as \code{x}.
normalize_log2 <- function(x, pseudocount = 1) {
  if (pseudocount <= 0) stop("`pseudocount` must be > 0.")
  if (any(as.numeric(x) < 0, na.rm = TRUE))
    stop("`x` has negative values; input should be non-negative (not log-scale).")
  log2(x + pseudocount)
}

# ---- Example (simulated data; select this block and run) --------------------
if (FALSE) {
  set.seed(1)
  tpm <- matrix(rgamma(50, shape = 2, scale = 10), nrow = 10,
                dimnames = list(paste0("Gene", 1:10), paste0("S", 1:5)))
  logmat <- normalize_log2(tpm)        # log2(TPM + 1)
  range(logmat)
}
# ==============================================================================
# quantile_stratify()  —  Split a numeric vector into ordered quantile groups
#
# Method : Cut a continuous variable (e.g. gene expression, a risk score) at its
#          own sample quantiles into 2 groups ("median"), 3 ("tertile") or 4
#          ("quartile"), returning an ordered factor low -> high for direct use
```

```r
#                   in survival or group-comparison models. Breaks come from
#                   quantile(type = 7); the lowest bin is left-closed so the minimum is
#                   included. When too many tied values make the quantile breaks
#                   non-unique the function stops rather than silently merging levels.
# Input    : x    numeric vector.
# Output : an ordered factor the same length as x (NA stays NA).
# Depends: base R only.
# ============================================================================

#' Split a numeric vector into ordered quantile groups
#'
#' @param x numeric vector.
#' @param groups "median" (2), "tertile" (3) or "quartile" (4).
#' @param labels optional character labels (length 2/3/4). Defaults are
#'     c("Low","High"); c("Low","Mid","High"); c("Q1","Q2","Q3","Q4").
#' @return an ordered factor, low -> high, of length \code{length(x)}.
quantile_stratify <- function(x, groups = c("median", "tertile", "quartile"),
                              labels = NULL) {
  groups <- match.arg(groups)
  k <- c(median = 2L, tertile = 3L, quartile = 4L)[[groups]]
  if (is.null(labels))
    labels <- switch(groups,
      median     = c("Low", "High"),
      tertile    = c("Low", "Mid", "High"),
      quartile = c("Q1", "Q2", "Q3", "Q4"))
  if (length(labels) != k) stop("`labels` must have length ", k, ".")

  x    <- as.numeric(x)
  br <- stats::quantile(x, probs = seq(0, 1, length.out = k + 1), na.rm = TRUE)
  if (anyDuplicated(br))
    stop("quantile breaks are not unique (too many tied values) for '", groups, "'.")

  cut(x, breaks = br, labels = labels, include.lowest = TRUE, ordered_result = TRUE)
}

# ---- Example (simulated data; select this block and run) --------------------
if (FALSE) {
  set.seed(1)
  score <- rnorm(100)
  grp     <- quantile_stratify(score, "tertile")
  table(grp)                                   # ~ 33 / 33 / 34, ordered Low < Mid < High
}
# ============================================================================
# zscore_by_group()  —  Row z-score computed separately within sample groups
#
# Method : For every feature (row), standardize its values to zero mean and unit
#                   SD *within each sample group independently*, using that group's own
#                   mean and SD. Because each cohort/batch is centred and scaled on its
#                   own location and spread, additive and multiplicative batch shifts are
#                   removed while within-group variation is retained. Groups with a zero
#                   or non-finite SD are mean-centred only (SD treated as 1) to avoid
#                   dividing by zero. This is a light-touch alternative to model-based
#                   batch correction when only relative, within-cohort variation matters.
# Input    : x          numeric matrix, features x samples.
#                   group    vector of length ncol(x) giving each sample's group/batch.
# Output : a numeric matrix the same shape/dimnames as x, row-z-scored per group.
# Depends: base R only.
```

```r
# ==============================================================================

#' Row z-score within sample groups
#'
#' @param x numeric matrix (features x samples).
#' @param group vector of length \code{ncol(x)} labelling each sample's group.
#' @return a numeric matrix, same dim/dimnames as \code{x}.
zscore_by_group <- function(x, group) {
  x <- as.matrix(x)
  if (length(group) != ncol(x))
    stop("length(group) must equal ncol(x).")
  group <- as.factor(group)

  out <- x
  for (g in levels(group)) {
    j <- which(group == g)
    block <- x[, j, drop = FALSE]
    mu    <- rowMeans(block, na.rm = TRUE)
    sdv <- apply(block, 1L, stats::sd, na.rm = TRUE)
    sdv[!is.finite(sdv) | sdv == 0] <- 1
    out[, j] <- (block - mu) / sdv
  }
  dimnames(out) <- dimnames(x)
  out
}

# ---- Example (simulated data; select this block and run) --------------------
if (FALSE) {
  set.seed(1)
  x <- matrix(rnorm(60), nrow = 10,
              dimnames = list(paste0("Gene", 1:10), paste0("S", 1:6)))
  x[, 4:6] <- x[, 4:6] + 5              # additive shift in cohort B
  batch <- rep(c("A", "B"), each = 3)
  z <- zscore_by_group(x, batch)
  round(tapply(z[1, ], batch, mean), 6)     # ~ 0 within every group
}
# ==============================================================================
# zscore_standardize()  —  Z-score a matrix by row or column, with optional cap
#
# Method : Standardize a numeric matrix to zero mean and unit standard deviation
#          along one margin: "row" scales each feature (gene) across samples,
#          "column" scales each sample across features. Means and SDs use
#          na.rm = TRUE; a margin with zero or non-finite SD is returned centred
#          (all zeros) to avoid dividing by zero. Optionally the result is
#          winsorized to +/- k SD to limit outlier influence, a common step
#          before heatmap display.
# Input   : x   numeric matrix (typically genes x samples).
# Output : a numeric matrix the same shape/dimnames as x, z-scored along margin.
# Depends: base R only.
# ==============================================================================

#' Z-score a matrix by row or column
#'
#' @param x numeric matrix.
#' @param margin "row" (default) standardizes each row; "column" each column.
#' @param cap optional positive number; clamp z-scores to \code{[-cap, cap]}.
#' @return a numeric matrix of z-scores, same dim/dimnames as \code{x}.
```

```r
zscore_standardize <- function(x, margin = c("row", "column"), cap = NULL) {
  margin <- match.arg(margin)
  x <- as.matrix(x)

  z <- function(v) {
    m <- mean(v, na.rm = TRUE); s <- stats::sd(v, na.rm = TRUE)
    if (!is.finite(s) || s == 0) return((v - m) * 0)
    (v - m) / s
  }

  out <- if (margin == "row") t(apply(x, 1L, z)) else apply(x, 2L, z)
  dimnames(out) <- dimnames(x)

  if (!is.null(cap)) {
    cap <- abs(cap)
    out[!is.na(out) & out >  cap] <-  cap
    out[!is.na(out) & out < -cap] <- -cap
  }
  out
}

# ---- Example (simulated data; select this block and run) --------------------
if (FALSE) {
  set.seed(1)
  x <- matrix(rnorm(50, mean = 5, sd = 2), nrow = 10,
              dimnames = list(paste0("Gene", 1:10), paste0("S", 1:5)))
  z <- zscore_standardize(x, margin = "row", cap = 2)
  round(rowMeans(z), 6)             # ~ 0 for every gene
}
# =============================================================================
# diff_expr_limma() — Differential expression by limma (case vs control)
#
# Method : Linear models for microarray / log-scale RNA-seq data (limma). A
#          per-group design is fit with lmFit, the case-minus-control contrast
#          is formed with makeContrasts, moderated statistics are computed by
#          empirical-Bayes shrinkage (eBayes), and the full result table is
#          returned by topTable with Benjamini-Hochberg FDR.
# Input  : expr  numeric matrix, genes in rows (rownames = gene symbols),
#                samples in columns; values on a log scale, e.g. log2(TPM + 1).
#          group vector of length ncol(expr) with exactly two levels.
# Output : topTable data.frame (gene, logFC, AveExpr, t, P.Value, adj.P.Val, B),
#          ordered by P.Value. Positive logFC means higher in the case group.
# Depends: limma (Bioconductor).
# =============================================================================

#' Differential expression by limma (case vs control)
#'
#' @param expr numeric matrix (genes x samples); rownames are gene symbols,
#'   values on a log scale.
#' @param group vector of length \code{ncol(expr)} giving each sample's group;
#'   must have exactly two distinct levels.
#' @param case optional; the level of \code{group} used as the "case" (contrast
#'   minuend). Defaults to the second level of \code{factor(group)}.
#' @return \code{\link[limma]{topTable}} data.frame with a leading \code{gene}
#'   column, ordered by \code{P.Value}.
diff_expr_limma <- function(expr, group, case = NULL) {
  expr <- as.matrix(expr)
```

```r
  if (length(group) != ncol(expr))
    stop("length(group) must equal ncol(expr).")
  group <- as.factor(group)
  if (nlevels(group) != 2L)
    stop("`group` must have exactly two levels; got ", nlevels(group), ".")

  levs <- levels(group)
  if (is.null(case)) case <- levs[2L]
  if (!case %in% levs)
    stop("`case` must be one of: ", paste(levs, collapse = ", "))
  control <- setdiff(levs, case)

  # Relabel to syntactically clean design columns so the contrast is well-formed.
  lab     <- factor(ifelse(group == case, "case", "control"),
                    levels = c("control", "case"))
  design <- stats::model.matrix(~ 0 + lab)
  colnames(design) <- levels(lab)

  fit      <- limma::lmFit(expr, design)
  contrast <- limma::makeContrasts(contrasts = "case - control", levels = design)
  fit2     <- limma::eBayes(limma::contrasts.fit(fit, contrast))
  tt <- limma::topTable(fit2, adjust.method = "BH", number = Inf, sort.by = "P")
  data.frame(gene = rownames(tt), tt, row.names = NULL, check.names = FALSE)
}

# ---- Example (simulated data; select this block and run) --------------------
if (FALSE) {
  set.seed(1)
  expr <- matrix(rnorm(200 * 20), nrow = 200,
                 dimnames = list(paste0("Gene", 1:200), paste0("S", 1:20)))
  group <- rep(c("Tumor", "Normal"), each = 10)
  expr[1:5, group == "Tumor"] <- expr[1:5, group == "Tumor"] + 2   # spike DE genes
  res <- diff_expr_limma(expr, group, case = "Tumor")
  head(res)
}
# ============================================================================
# diff_expr_limma_highlow()  —  limma DE between High and Low expressors of a
#                               driver gene
#
# Method : Samples are split into "High" and "Low" groups by the expression of
#          one driver gene, then differential expression is computed with limma
#          exactly as for a two-group case/control comparison (lmFit ->
#          makeContrasts(High - Low) -> eBayes -> topTable, Benjamini-Hochberg
#          FDR). The cut point is the median or mean of the driver, or  —  with
#          "quantile"  —  the lower vs upper tertile with the middle third dropped.
# Input  : expr   numeric matrix, genes in rows (rownames = gene symbols),
#                 samples in columns; values on a log scale, e.g. log2(TPM + 1).
#          gene   a single driver gene symbol present in rownames(expr).
# Output : topTable data.frame (gene, logFC, AveExpr, t, P.Value, adj.P.Val, B),
#          ordered by P.Value. Positive logFC means higher in the High group.
# Depends: limma (Bioconductor).
# ============================================================================

#' limma DE between High and Low expressors of a driver gene
#'
#' @param expr numeric matrix (genes x samples); rownames are gene symbols,
#'   values on a log scale.
```

```
#' @param gene single driver gene symbol; must be present in \code{rownames(expr)}.
#' @param cut how to dichotomize the driver: \code{"median"} or \code{"mean"}
#'     (High > threshold, Low <= threshold), or \code{"quantile"} (lower tertile =
#'     Low, upper tertile = High, middle third dropped).
#' @return \code{\link[limma]{topTable}} data.frame with a leading \code{gene}
#'     column, ordered by \code{P.Value}.
diff_expr_limma_highlow <- function(expr, gene,
                                    cut = c("median", "mean", "quantile")) {
  expr <- as.matrix(expr)
  cut   <- match.arg(cut)
  if (!gene %in% rownames(expr))
    stop("`gene` not found in rownames(expr): ", gene)

  driver <- expr[gene, ]
  keep    <- is.finite(driver)
  grp     <- rep(NA_character_, length(driver))
  if (cut == "quantile") {
    qs <- stats::quantile(driver[keep], probs = c(1/3, 2/3))
    grp[keep & driver <= qs[1L]] <- "Low"
    grp[keep & driver >= qs[2L]] <- "High"     # middle tertile stays NA (dropped)
  } else {
    thr <- if (cut == "median") stats::median(driver[keep]) else mean(driver[keep])
    grp[keep & driver <   thr] <- "Low"
    grp[keep & driver >= thr] <- "High"
  }

  sel    <- !is.na(grp)
  group <- factor(grp[sel], levels = c("Low", "High"))
  if (nlevels(droplevels(group)) != 2L || min(table(group)) < 2L)
    stop("Both High and Low groups need at least 2 samples after cutting.")
  mat <- expr[, sel, drop = FALSE]

  design <- stats::model.matrix(~ 0 + group)
  colnames(design) <- levels(group)
  fit          <- limma::lmFit(mat, design)
  contrast <- limma::makeContrasts(contrasts = "High - Low", levels = design)
  fit2         <- limma::eBayes(limma::contrasts.fit(fit, contrast))
  tt <- limma::topTable(fit2, adjust.method = "BH", number = Inf, sort.by = "P")
  data.frame(gene = rownames(tt), tt, row.names = NULL, check.names = FALSE)
}

# ---- Example (simulated data; select this block and run) --------------------
if (FALSE) {
  set.seed(1)
  expr <- matrix(rnorm(200 * 30), nrow = 200,
                 dimnames = list(paste0("Gene", 1:200), paste0("S", 1:30)))
  # make a few genes track the driver so there is real High-vs-Low signal
  expr["Gene1", ] <- rnorm(30)
  expr[2:6, ]         <- sweep(expr[2:6, ], 2, expr["Gene1", ], `+`)
  res <- diff_expr_limma_highlow(expr, gene = "Gene1", cut = "median")
  head(res)
}
# ============================================================================
# diff_expr_wilcoxon()  —  Differential expression by Wilcoxon rank-sum test
#
# Method : Per-gene two-group Wilcoxon rank-sum (Mann-Whitney U) test on an
#                 expression matrix, with Benjamini-Hochberg FDR control. The log2
```

```
#                   fold change is the difference of group means (the input is assumed to
#                   be on a log scale, so a mean difference equals a log2 fold change).
# Input    : expr    numeric matrix, genes in rows (rownames = gene symbols),
#                              samples in columns; values on a log scale, e.g. log2(TPM + 1).
#                   group vector of length ncol(expr) with exactly two levels.
# Output : data.frame(gene, mean_<case>, mean_<control>, log2FC, W, p.value,
#                   p.adj), ordered by p.value. Compute only  —  no plotting.
# Depends: base R only.
# ============================================================================

#' Differential expression by Wilcoxon rank-sum test
#'
#' @param expr numeric matrix (genes x samples); rownames are gene symbols,
#'      values on a log scale.
#' @param group vector of length \code{ncol(expr)} giving each sample's group;
#'      must have exactly two distinct levels.
#' @param case optional; the level of \code{group} used as the "case" (fold-change
#'      numerator). Defaults to the second level of \code{factor(group)}.
#' @param p_adjust multiple-testing method for \code{\link[stats]{p.adjust}}
#'      (default "BH").
#' @param min_samples minimum finite samples required per group to test a gene
#'      (default 3).
#' @return data.frame, one row per testable gene, ordered by \code{p.value}.
diff_expr_wilcoxon <- function(expr, group, case = NULL,
                                                          p_adjust = "BH", min_samples = 3) {
   expr <- as.matrix(expr)
   if (length(group) != ncol(expr))
      stop("length(group) must equal ncol(expr).")
   group <- as.factor(group)
   if (nlevels(group) != 2L)
      stop("`group` must have exactly two levels; got ", nlevels(group), ".")

   levs <- levels(group)
   if (is.null(case)) case <- levs[2L]
   if (!case %in% levs)
      stop("`case` must be one of: ", paste(levs, collapse = ", "))
   control <- setdiff(levs, case)
   i_case    <- which(group == case)
   i_ctrl    <- which(group == control)

   stat <- t(vapply(seq_len(nrow(expr)), function(g) {
      x <- expr[g, i_case]; y <- expr[g, i_ctrl]
      x <- x[is.finite(x)]; y <- y[is.finite(y)]
      if (length(x) < min_samples || length(y) < min_samples)
         return(c(mc = NA_real_, mk = NA_real_, lfc = NA_real_, W = NA_real_, p = NA_real_))
      ht <- suppressWarnings(stats::wilcox.test(x, y))
      c(mc = mean(x), mk = mean(y), lfc = mean(x) - mean(y),
         W = unname(ht$statistic), p = ht$p.value)
   }, numeric(5)))

   out <- data.frame(gene = rownames(expr), stat,
                                    p.adj = stats::p.adjust(stat[, "p"], method = p_adjust),
                                    row.names = NULL, check.names = FALSE)
   colnames(out) <- c("gene", paste0("mean_", case), paste0("mean_", control),
                                     "log2FC", "W", "p.value", "p.adj")
   out <- out[order(out$p.value), ]
   rownames(out) <- NULL
```

```
  out
}

# ---- Example (simulated data; select this block and run) --------------------
if (FALSE) {
  set.seed(1)
  expr <- matrix(rnorm(200 * 20), nrow = 200,
                 dimnames = list(paste0("Gene", 1:200), paste0("S", 1:20)))
  expr["Gene1", 1:10] <- expr["Gene1", 1:10] + 3      # spike one true DE gene
  group <- rep(c("Tumor", "Normal"), each = 10)
  res <- diff_expr_wilcoxon(expr, group, case = "Tumor")
  head(res)
}
# =============================================================================
# diff_expr_wilcoxon_paired()  —  Paired differential expression by Wilcoxon
#                                  signed-rank test
#
# Method : Per-gene paired Wilcoxon signed-rank test on matched samples (e.g.
#          tumor vs adjacent-normal from the same subject), with Benjamini-
#          Hochberg FDR control. Samples are aligned into pairs by a subject id;
#          only subjects present in both conditions are used. The log2 fold
#          change is the mean of the within-pair differences (the input is
#          assumed to be on a log scale).
# Input  : expr      numeric matrix, genes in rows (rownames = gene symbols),
#                    samples in columns; values on a log scale, e.g. log2(TPM+1).
#          subject   vector of length ncol(expr): the pairing id per sample.
#          condition vector of length ncol(expr) with exactly two levels.
# Output : data.frame(gene, log2FC, V, p.value, p.adj), ordered by p.value.
#          Compute only  —  no plotting.
# Depends: base R only.
# =============================================================================

#' Paired differential expression by Wilcoxon signed-rank test
#'
#' @param expr numeric matrix (genes x samples); rownames are gene symbols,
#'   values on a log scale.
#' @param subject vector of length \code{ncol(expr)} giving each sample's
#'   pairing id (e.g. subject/patient identifier).
#' @param condition vector of length \code{ncol(expr)} giving each sample's
#'   condition; must have exactly two distinct levels.
#' @param case optional; the level of \code{condition} used as the "case"
#'   (difference minuend). Defaults to the second level of \code{factor}.
#' @param p_adjust multiple-testing method for \code{\link[stats]{p.adjust}}
#'   (default "BH").
#' @param min_pairs minimum complete pairs required to test a gene (default 3).
#' @return data.frame, one row per testable gene, ordered by \code{p.value}.
diff_expr_wilcoxon_paired <- function(expr, subject, condition, case = NULL,
                                      p_adjust = "BH", min_pairs = 3) {
  expr <- as.matrix(expr)
  if (length(subject) != ncol(expr))
    stop("length(subject) must equal ncol(expr).")
  if (length(condition) != ncol(expr))
    stop("length(condition) must equal ncol(expr).")
  subject   <- as.character(subject)
  condition <- as.factor(condition)
  if (nlevels(condition) != 2L)
    stop("`condition` must have exactly two levels; got ", nlevels(condition), ".")
```

```r
    levs <- levels(condition)
    if (is.null(case)) case <- levs[2L]
    if (!case %in% levs)
        stop("`case` must be one of: ", paste(levs, collapse = ", "))
    control <- setdiff(levs, case)

    # Keep only subjects with a sample in BOTH conditions; align the two arms so
    # column j of each arm is the same subject.
    paired <- intersect(subject[condition == case], subject[condition == control])
    if (length(paired) < min_pairs)
        stop("Fewer than ", min_pairs, " subjects have both conditions.")
    col_case <- vapply(paired, function(s)
        which(subject == s & condition == case)[1L], integer(1))
    col_ctrl <- vapply(paired, function(s)
        which(subject == s & condition == control)[1L], integer(1))

    stat <- t(vapply(seq_len(nrow(expr)), function(g) {
        x <- expr[g, col_case]; y <- expr[g, col_ctrl]
        ok <- is.finite(x) & is.finite(y)
        x <- x[ok]; y <- y[ok]
        if (length(x) < min_pairs)
            return(c(lfc = NA_real_, V = NA_real_, p = NA_real_))
        ht <- suppressWarnings(stats::wilcox.test(x, y, paired = TRUE))
        c(lfc = mean(x - y), V = unname(ht$statistic), p = ht$p.value)
    }, numeric(3)))

    out <- data.frame(gene = rownames(expr), stat,
                                    p.adj = stats::p.adjust(stat[, "p"], method = p_adjust),
                                    row.names = NULL, check.names = FALSE)
    colnames(out) <- c("gene", "log2FC", "V", "p.value", "p.adj")
    out <- out[order(out$p.value), ]
    rownames(out) <- NULL
    out
}

# ---- Example (simulated data; select this block and run) --------------------
if (FALSE) {
    set.seed(1)
    n_subj <- 12
    expr <- matrix(rnorm(200 * 2 * n_subj), nrow = 200,
                              dimnames = list(paste0("Gene", 1:200),
                                                          paste0("S", 1:(2 * n_subj))))
    subject      <- rep(paste0("subj", 1:n_subj), times = 2)
    condition <- rep(c("Tumor", "Normal"), each = n_subj)
    # spike a consistent within-subject shift for one gene
    expr["Gene1", condition == "Tumor"] <- expr["Gene1", condition == "Tumor"] + 2
    res <- diff_expr_wilcoxon_paired(expr, subject, condition, case = "Tumor")
    head(res)
}
# ==============================================================================
# correlation_batch_target_vs_all()  —  One column vs all others in a table
#
# Method : Correlate a target column against every other column of a numeric
#                  table (observations in rows, variables in columns), one
#                  stats::cor.test per variable (Pearson or Spearman) on rows where both
#                  values are finite. Benjamini-Hochberg adjustment across variables.
```

```r
# Input    : data       numeric data.frame or matrix; observations in rows, variables
#                         in columns (colnames = variable names).
#              target name of the target column; must be a column of `data`.
#              method "pearson" (default) or "spearman".
# Output : data.frame(variable, cor, p.value, p.adj, n) ordered by p.value.
#              Compute only  —  no plotting.
# Depends: base R only.
# ============================================================================

#' Correlate one column against all other columns of a table
#'
#' @param data numeric data.frame or matrix; observations in rows, variables in
#'     columns.
#' @param target name of the target column to correlate against all others.
#' @param method correlation method, "pearson" (default) or "spearman".
#' @param p_adjust multiple-testing method for \code{\link[stats]{p.adjust}}
#'     (default "BH").
#' @param min_pairs minimum complete (both-finite) row pairs per variable
#'     (default 3).
#' @return data.frame, one row per other variable, ordered by \code{p.value}.
correlation_batch_target_vs_all <- function(data, target,
                                             method = c("pearson", "spearman"),
                                             p_adjust = "BH", min_pairs = 3) {
  method <- match.arg(method)
  data     <- as.data.frame(data)
  if (!target %in% colnames(data))
    stop("`target` (", target, ") is not a column of `data`.")
  t_vec    <- as.numeric(data[[target]])
  others <- setdiff(colnames(data), target)
  if (!length(others))
    stop("`data` has no columns other than `target`.")

  stat <- t(vapply(others, function(v) {
    y    <- as.numeric(data[[v]])
    ok <- is.finite(t_vec) & is.finite(y)
    n    <- sum(ok)
    if (n < min_pairs) return(c(cor = NA_real_, p = NA_real_, n = n))
    ht <- suppressWarnings(stats::cor.test(t_vec[ok], y[ok], method = method))
    c(cor = unname(ht$estimate), p = ht$p.value, n = n)
  }, numeric(3)))

  out <- data.frame(variable = others, cor = stat[, "cor"],
                    p.value = stat[, "p"],
                    p.adj = stats::p.adjust(stat[, "p"], method = p_adjust),
                    n = stat[, "n"], row.names = NULL)
  out <- out[order(out$p.value), ]
  rownames(out) <- NULL
  out
}

# ---- Example (simulated data; select this block and run) --------------------
if (FALSE) {
  set.seed(1)
  data <- as.data.frame(matrix(rnorm(40 * 6), nrow = 40,
                               dimnames = list(NULL, paste0("V", 1:6))))
  data$V2 <- 0.7 * data$V1 + rnorm(40, sd = 0.5)    # V2 tracks V1
  res <- correlation_batch_target_vs_all(data, target = "V1")
```

```r
  res
}
# ==========================================================================
# correlation_cross_omics()  —  Per-feature correlation between two omics layers
#
# Method : Given two matrices measuring the same features on the same samples in
#          different omics layers (for example mRNA vs DNA-methylation, copy
#          number, or miRNA of the same genes), align them to their shared
#          features (rownames) and shared samples (colnames), then run one
#          stats::cor.test per shared feature across samples (Pearson or
#          Spearman). Benjamini-Hochberg adjustment across features.
# Input  : x, y    numeric matrices, features in rows (rownames = feature IDs),
#                  samples in columns (colnames = sample IDs); the two layers to
#                  correlate feature-by-feature.
#          method "pearson" (default) or "spearman".
# Output : data.frame(feature, cor, p.value, p.adj, n) ordered by p.value.
#          Compute only  —  no plotting.
# Depends: base R only.
# ==========================================================================

#' Per-feature correlation between two matched omics matrices
#'
#' @param x,y numeric matrices (features x samples); rownames are feature IDs,
#'   colnames are sample IDs. Correlated feature-by-feature across shared
#'   samples.
#' @param method correlation method, "pearson" (default) or "spearman".
#' @param p_adjust multiple-testing method for \code{\link[stats]{p.adjust}}
#'   (default "BH").
#' @param min_pairs minimum complete (both-finite) sample pairs per feature
#'   (default 3).
#' @return data.frame, one row per shared feature, ordered by \code{p.value}.
correlation_cross_omics <- function(x, y, method = c("pearson", "spearman"),
                                    p_adjust = "BH", min_pairs = 3) {
  method <- match.arg(method)
  x <- as.matrix(x); y <- as.matrix(y)
  feats <- intersect(rownames(x), rownames(y))
  samps <- intersect(colnames(x), colnames(y))
  if (length(feats) < 1L)
    stop("no shared features (rownames) between `x` and `y`.")
  if (length(samps) < min_pairs)
    stop("only ", length(samps), " shared samples; need >= ", min_pairs, ".")
  x <- x[feats, samps, drop = FALSE]
  y <- y[feats, samps, drop = FALSE]

  stat <- t(vapply(feats, function(f) {
    a  <- as.numeric(x[f, ]); b <- as.numeric(y[f, ])
    ok <- is.finite(a) & is.finite(b)
    n  <- sum(ok)
    if (n < min_pairs) return(c(cor = NA_real_, p = NA_real_, n = n))
    ht <- suppressWarnings(stats::cor.test(a[ok], b[ok], method = method))
    c(cor = unname(ht$estimate), p = ht$p.value, n = n)
  }, numeric(3)))

  out <- data.frame(feature = feats, cor = stat[, "cor"],
                    p.value = stat[, "p"],
                    p.adj = stats::p.adjust(stat[, "p"], method = p_adjust),
                    n = stat[, "n"], row.names = NULL)
```

```
  out <- out[order(out$p.value), ]
  rownames(out) <- NULL
  out
}

# ---- Example (simulated data; select this block and run) --------------------
if (FALSE) {
  set.seed(1)
  feats <- paste0("Gene", 1:20); samps <- paste0("S", 1:30)
  mrna   <- matrix(rnorm(20 * 30), 20, dimnames = list(feats, samps))
  meth   <- matrix(rnorm(20 * 30), 20, dimnames = list(feats, samps))
  meth["Gene1", ] <- -0.8 * mrna["Gene1", ] + rnorm(30, sd = 0.3)   # anti-corr
  res <- correlation_cross_omics(mrna, meth, method = "pearson")
  head(res)
}
# ============================================================================
# correlation_gene_vs_genome()  —  One gene vs every other gene in a matrix
#
# Method : Correlate a target gene's expression against every other row of an
#              expression matrix, one stats::cor.test per gene (Pearson or
#              Spearman) using only samples where both values are finite. P-values
#              are Benjamini-Hochberg adjusted across all tested genes.
# Input   : expr     numeric matrix, genes in rows (rownames = gene symbols),
#                        samples in columns.
#              target gene symbol; must be a rowname of expr.
#              method "pearson" (default) or "spearman".
# Output : data.frame(gene, cor, p.value, p.adj, n) ordered by p.value.
#              Compute only  —  no plotting.
# Depends: base R only.
# ============================================================================

#' Correlate one gene against every other gene (genome-wide)
#'
#' @param expr numeric matrix (genes x samples); rownames are gene symbols.
#' @param target gene symbol to correlate against all others; a rowname of expr.
#' @param method correlation method, "pearson" (default) or "spearman".
#' @param p_adjust multiple-testing method for \code{\link[stats]{p.adjust}}
#'     (default "BH").
#' @param min_pairs minimum complete (both-finite) sample pairs per gene
#'     (default 3).
#' @return data.frame, one row per other gene, ordered by \code{p.value}.
correlation_gene_vs_genome <- function(expr, target,
                                       method = c("pearson", "spearman"),
                                       p_adjust = "BH", min_pairs = 3) {
  method <- match.arg(method)
  expr <- as.matrix(expr)
  if (!target %in% rownames(expr))
    stop("`target` (", target, ") is not a rowname of `expr`.")
  t_vec   <- as.numeric(expr[target, ])
  others <- setdiff(rownames(expr), target)
  if (!length(others))
    stop("`expr` has no genes other than `target`.")

  stat <- t(vapply(others, function(g) {
    y   <- as.numeric(expr[g, ])
    ok <- is.finite(t_vec) & is.finite(y)
    n   <- sum(ok)
```

```
    if (n < min_pairs) return(c(cor = NA_real_, p = NA_real_, n = n))
    ht <- suppressWarnings(stats::cor.test(t_vec[ok], y[ok], method = method))
    c(cor = unname(ht$estimate), p = ht$p.value, n = n)
  }, numeric(3)))

  out <- data.frame(gene = others, cor = stat[, "cor"],
                    p.value = stat[, "p"],
                    p.adj = stats::p.adjust(stat[, "p"], method = p_adjust),
                    n = stat[, "n"], row.names = NULL)
  out <- out[order(out$p.value), ]
  rownames(out) <- NULL
  out
}

# ---- Example (simulated data; select this block and run) --------------------
if (FALSE) {
  set.seed(1)
  expr <- matrix(rnorm(50 * 30), nrow = 50,
                 dimnames = list(paste0("Gene", 1:50), paste0("S", 1:30)))
  expr["Gene2", ] <- expr["Gene1", ] + rnorm(30, sd = 0.3)   # Gene2 tracks Gene1
  res <- correlation_gene_vs_genome(expr, target = "Gene1", method = "pearson")
  head(res)
}
# ==============================================================================
# correlation_gene_vs_signature()  —  One gene vs each column of a score matrix
#
# Method : Correlate one gene's expression against each feature (column) of a
#          per-sample score matrix  —  for example immune-infiltration fractions,
#          stemness indices, or pathway-activity scores. Samples are aligned by
#          name (names of the gene vector vs rownames of the score matrix); one
#          stats::cor.test per feature (Spearman by default) on the shared
#          samples where both values are finite; Benjamini-Hochberg across
#          features.
# Input  : gene    named numeric vector; names = sample IDs, values = expression.
#          scores samples x features numeric matrix / data.frame; rownames =
#                 sample IDs, one column per feature/signature.
#          method "spearman" (default) or "pearson".
# Output : data.frame(feature, rho, p.value, p.adj, n) ordered by p.value.
#          Compute only  —  no plotting.
# Depends: base R only.
# ==============================================================================

#' Correlate one gene against each column of a per-sample score matrix
#'
#' @param gene named numeric vector of expression; names are sample IDs.
#' @param scores samples x features numeric matrix or data.frame; rownames are
#'   sample IDs, columns are features/signatures.
#' @param method correlation method, "spearman" (default) or "pearson".
#' @param p_adjust multiple-testing method for \code{\link[stats]{p.adjust}}
#'   (default "BH").
#' @param min_pairs minimum complete (both-finite) sample pairs per feature
#'   (default 3).
#' @return data.frame, one row per feature, ordered by \code{p.value}. The
#'   \code{rho} column holds the correlation coefficient for the chosen method.
correlation_gene_vs_signature <- function(gene, scores,
                                          method = c("spearman", "pearson"),
                                          p_adjust = "BH", min_pairs = 3) {
```

```
  method <- match.arg(method)
  scores <- as.matrix(scores)
  if (is.null(names(gene)) || is.null(rownames(scores)))
    stop("`gene` must be named by sample and `scores` must have rownames.")
  common <- intersect(names(gene), rownames(scores))
  if (length(common) < min_pairs)
    stop("only ", length(common), " shared samples; need >= ", min_pairs, ".")
  g      <- as.numeric(gene[common])
  scores <- scores[common, , drop = FALSE]
  feats  <- colnames(scores)
  if (is.null(feats)) feats <- paste0("feature", seq_len(ncol(scores)))

  stat <- t(vapply(seq_len(ncol(scores)), function(j) {
    y  <- as.numeric(scores[, j])
    ok <- is.finite(g) & is.finite(y)
    n  <- sum(ok)
    if (n < min_pairs) return(c(rho = NA_real_, p = NA_real_, n = n))
    ht <- suppressWarnings(stats::cor.test(g[ok], y[ok], method = method))
    c(rho = unname(ht$estimate), p = ht$p.value, n = n)
  }, numeric(3)))

  out <- data.frame(feature = feats, rho = stat[, "rho"],
                    p.value = stat[, "p"],
                    p.adj = stats::p.adjust(stat[, "p"], method = p_adjust),
                    n = stat[, "n"], row.names = NULL)
  out <- out[order(out$p.value), ]
  rownames(out) <- NULL
  out
}

# ---- Example (simulated data; select this block and run) --------------------
if (FALSE) {
  set.seed(1)
  samples <- paste0("S", 1:40)
  gene    <- setNames(rnorm(40), samples)
  scores  <- matrix(rnorm(40 * 5), nrow = 40,
                    dimnames = list(samples, paste0("Sig", 1:5)))
  scores[, "Sig1"] <- 0.6 * gene + rnorm(40, sd = 0.5)   # Sig1 tracks the gene
  res <- correlation_gene_vs_signature(gene, scores, method = "spearman")
  res
}
# ============================================================================
# correlation_matrix()  —  Pairwise correlation matrix over selected genes
#
# Method : Subset an expression matrix to a chosen set of genes and compute the
#          full pairwise correlation matrix (Pearson or Spearman) with base
#          cor() using pairwise-complete observations, plus a matching matrix of
#          cor.test p-values from a symmetric pairwise loop. (Hmisc::rcorr is a
#          faster one-call alternative returning r, n and P; it is not required
#          here.)
# Input  : expr    numeric matrix, genes in rows (rownames = gene symbols),
#                  samples in columns.
#          genes   character vector of gene symbols to include (>= 2); each must
#                  be a rowname of expr.
#          method  "pearson" (default) or "spearman".
# Output : list(r, p)  —  r is the genes x genes correlation matrix, p the
#          matching p-value matrix (self-correlations on the diagonal: r = 1,
```

```r
#                    p = NA). Compute only  –  no plotting.
# Depends: base R only.
# ============================================================================

#' Pairwise correlation matrix (r and p) over selected genes
#'
#' @param expr numeric matrix (genes x samples); rownames are gene symbols.
#' @param genes character vector of gene symbols to include (at least two);
#'      each must be a rowname of expr.
#' @param method correlation method, "pearson" (default) or "spearman".
#' @return list with \code{r} (correlation matrix) and \code{p} (p-value
#'      matrix), both genes x genes with identical dimnames.
correlation_matrix <- function(expr, genes,
                               method = c("pearson", "spearman")) {
  method <- match.arg(method)
  expr     <- as.matrix(expr)
  genes    <- unique(as.character(genes))
  miss     <- setdiff(genes, rownames(expr))
  if (length(miss))
    stop("genes not found in `expr`: ", paste(miss, collapse = ", "))
  if (length(genes) < 2L)
    stop("need at least two distinct genes.")

  # samples in columns -> transpose so genes become variables (columns)
  m <- t(expr[genes, , drop = FALSE])
  r <- stats::cor(m, method = method, use = "pairwise.complete.obs")

  p <- matrix(NA_real_, nrow = length(genes), ncol = length(genes),
              dimnames = list(genes, genes))
  for (i in seq_len(length(genes) - 1L)) {
    for (j in seq.int(i + 1L, length(genes))) {
      ht <- suppressWarnings(
        stats::cor.test(m[, i], m[, j], method = method))
      p[i, j] <- p[j, i] <- ht$p.value
    }
  }
  list(r = r, p = p)
}

# ---- Example (simulated data; select this block and run) --------------------
if (FALSE) {
  set.seed(1)
  expr <- matrix(rnorm(10 * 40), nrow = 10,
                 dimnames = list(paste0("Gene", 1:10), paste0("S", 1:40)))
  expr["Gene2", ] <- expr["Gene1", ] + rnorm(40, sd = 0.4)    # Gene1 ~ Gene2
  res <- correlation_matrix(expr, genes = paste0("Gene", 1:5))
  round(res$r, 2)
  signif(res$p, 2)
}
# ============================================================================
# correlation_two_genes()  –  Correlation between two genes (or two vectors)
#
# Method : Pearson or Spearman correlation between two numeric vectors paired by
#                   sample (e.g. the expression of two genes across the same samples) via
#                   a single stats::cor.test. Only pairs where both values are finite are
#                   used (equivalent to use = "pairwise.complete.obs").
# Input    : x, y      numeric vectors of equal length, paired by sample (typically
```

```r
#                                  two rows pulled from an expression matrix).
#                   method "pearson" (default) or "spearman".
# Output : list(method, estimate, conf.int, statistic, p.value, n). conf.int is
#                   NULL for Spearman (cor.test returns none). Compute only  —  no plotting.
# Depends: base R only.
# ============================================================================

#' Correlation between two genes (or two numeric vectors)
#'
#' @param x,y numeric vectors of equal length, paired by sample.
#' @param method correlation method, "pearson" (default) or "spearman".
#' @param min_pairs minimum number of complete (both-finite) pairs required
#'      (default 3).
#' @return a list with the method, correlation estimate, confidence interval
#'      (Pearson only; NULL otherwise), test statistic, p-value, and number of
#'      complete pairs used.
correlation_two_genes <- function(x, y, method = c("pearson", "spearman"),
                                                      min_pairs = 3) {
   method <- match.arg(method)
   x <- as.numeric(x); y <- as.numeric(y)
   if (length(x) != length(y))
      stop("`x` and `y` must have the same length.")
   ok <- is.finite(x) & is.finite(y)
   n <- sum(ok)
   if (n < min_pairs)
      stop("need at least ", min_pairs, " complete pairs; got ", n, ".")

   ht <- suppressWarnings(stats::cor.test(x[ok], y[ok], method = method))
   list(method        = method,
           estimate    = unname(ht$estimate),
           conf.int    = if (is.null(ht$conf.int)) NULL else unname(ht$conf.int),
           statistic = unname(ht$statistic),
           p.value      = ht$p.value,
           n                 = n)
}

# ---- Example (simulated data; select this block and run) --------------------
if (FALSE) {
   set.seed(1)
   x <- rnorm(30)
   y <- 0.7 * x + rnorm(30, sd = 0.5)      # y correlated with x
   correlation_two_genes(x, y, method = "pearson")
   correlation_two_genes(x, y, method = "spearman")
}
# ============================================================================
# survival_cox_rcs()  —  Restricted cubic spline Cox regression
#
# Method : Model the (possibly non-linear) effect of a continuous variable on
#                   the hazard with a restricted cubic spline (rms::rcs) inside a Cox
#                   model (rms::cph). Test for non-linearity with the spline Wald test
#                   (anova), and return the dose-response curve as hazard ratios relative
#                   to a reference value across the observed range. Returns numbers only
#                   —  plotting is left to the caller.
# Input    : df    data.frame with columns: time (follow-up), status (1 = event,
#                         0 = censored), value (continuous variable).
# Output : list(nonlinear_p, ref, pred = data.frame(value, HR, lower, upper)).
#                   Compute only  —  no plotting.
```

```r
# Depends: rms (which imports survival).
# ==============================================================================

#' Restricted cubic spline Cox regression (compute only)
#'
#' @param df data.frame containing the time, status and value columns.
#' @param time,status,value column names in \code{df} (defaults "time",
#'     "status", "value"). \code{status} must be 1 = event, 0 = censored.
#' @param knots number of spline knots for \code{\link[rms]{rcs}} (default 4).
#' @param ref reference value the hazard ratios are computed against; defaults
#'     to the median of \code{value}.
#' @param n_pred number of points on the dose-response curve (default 100).
#' @return a list with the non-linearity p-value, the reference value used, and
#'     a data.frame of hazard ratios (with 95% CI) versus the variable.
survival_cox_rcs <- function(df, time = "time", status = "status",
                             value = "value", knots = 4, ref = NULL,
                             n_pred = 100) {
  for (col in c(time, status, value))
    if (!col %in% names(df)) stop("column not found: ", col)

  d <- data.frame(time   = as.numeric(df[[time]]),
                  status = as.integer(df[[status]]),
                  value  = as.numeric(df[[value]]))
  d <- d[stats::complete.cases(d) & d$time > 0, , drop = FALSE]
  if (nrow(d) < 20) stop("need >= 20 complete samples with positive time.")

  dd <- rms::datadist(d)
  if (!is.null(ref)) dd$limits["Adjust to", "value"] <- as.numeric(ref)
  old <- options(datadist = dd)
  on.exit(options(old), add = TRUE)
  ref_used <- as.numeric(dd$limits["Adjust to", "value"])

  fit <- rms::cph(survival::Surv(time, status) ~ rms::rcs(value, knots),
                  data = d, x = TRUE, y = TRUE)

  an      <- anova(fit)
  nl_row <- grep("Nonlinear", rownames(an))
  nonlinear_p <- if (length(nl_row)) unname(an[nl_row[1], "P"]) else NA_real_

  vals <- seq(min(d$value), max(d$value), length.out = n_pred)
  pr   <- rms::Predict(fit, value = vals, ref.zero = TRUE, fun = exp)
  pred <- data.frame(value = pr$value, HR = pr$yhat,
                     lower = pr$lower, upper = pr$upper, row.names = NULL)

  list(nonlinear_p = nonlinear_p, ref = ref_used, pred = pred)
}

# ---- Example (simulated data; select this block and run) --------------------
if (FALSE) {
  set.seed(1)
  n      <- 400
  value  <- runif(n, -3, 3)
  risk   <- 0.4 * value^2                    # U-shaped (non-linear) effect
  time   <- rexp(n, rate = 0.05 * exp(risk))
  status <- rbinom(n, 1, 0.7)
  df     <- data.frame(time = time, status = status, value = value)
  res <- survival_cox_rcs(df, knots = 4)
```

```
  res$nonlinear_p
  head(res$pred)
}
# ==============================================================================
# survival_km_median()  —  Kaplan-Meier stratification at the median of a
#                                          continuous marker
#
# Method : Dichotomize a continuous marker at its median (or at a user-supplied
#                  cut value) into High / Low groups, then compute the Kaplan-Meier
#                  fit (survival::survfit), the log-rank test (survival::survdiff) and
#                  the univariate Cox hazard ratio with 95% CI (survival::coxph).
#                  Returns numbers only  —  plotting is left to the caller.
# Input    : df    data.frame with columns: time (follow-up), status (1 = event,
#                        0 = censored), value (continuous marker).
# Output : list(cut, n_high, n_low, logrank_p, HR, HR_low, HR_high, cox_p,
#                  median_high, median_low). Compute only  —  no plotting.
# Depends: survival.
# ==============================================================================

#' Kaplan-Meier stratification at the median (compute only)
#'
#' @param df data.frame containing the time, status and marker columns.
#' @param time,status,value column names in \code{df} (defaults "time",
#'      "status", "value"). \code{status} must be 1 = event, 0 = censored.
#' @param cut either the string "median" (dichotomize at the sample median,
#'      the default) or a single numeric cut value; samples with
#'      \code{value > cut} are "High".
#' @return a list with the cut value used, group sizes, the log-rank p-value,
#'      the Cox HR with 95% CI and p-value, and median survival per group.
survival_km_median <- function(df, time = "time", status = "status",
                                               value = "value", cut = "median") {
  for (col in c(time, status, value))
    if (!col %in% names(df)) stop("column not found: ", col)

  d <- data.frame(time      = as.numeric(df[[time]]),
                          status = as.integer(df[[status]]),
                          value    = as.numeric(df[[value]]))
  d <- d[stats::complete.cases(d) & d$time > 0, , drop = FALSE]
  if (nrow(d) < 10) stop("need >= 10 complete samples with positive time.")

  cp <- if (is.numeric(cut)) cut[1L] else stats::median(d$value)
  d$group <- factor(ifelse(d$value > cp, "High", "Low"), levels = c("Low", "High"))
  if (nlevels(droplevels(d$group)) < 2L)
    stop("the cut value does not split the samples into two groups.")

  sd <- survival::survdiff(survival::Surv(time, status) ~ group, data = d)
  logrank_p <- stats::pchisq(sd$chisq, df = length(sd$n) - 1, lower.tail = FALSE)

  cox <- survival::coxph(survival::Surv(time, status) ~ group, data = d)
  sm    <- summary(cox)
  fit <- survival::survfit(survival::Surv(time, status) ~ group, data = d)
  med <- summary(fit)$table[, "median"]

  list(
    cut                = as.numeric(cp),
    n_high           = sum(d$group == "High"),
    n_low             = sum(d$group == "Low"),
```

```
        logrank_p      = unname(logrank_p),
        HR             = unname(sm$conf.int[1, "exp(coef)"]),
        HR_low         = unname(sm$conf.int[1, "lower .95"]),
        HR_high        = unname(sm$conf.int[1, "upper .95"]),
        cox_p          = unname(sm$coefficients[1, "Pr(>|z|)"]),
        median_high = unname(med[grep("High", names(med))]),
        median_low    = unname(med[grep("Low",    names(med))])
    )
}

# ---- Example (simulated data; select this block and run) --------------------
if (FALSE) {
    set.seed(1)
    n          <- 200
    value    <- rnorm(n)
    time      <- rexp(n, rate = 0.1 * exp(0.7 * as.numeric(scale(value))))
    status <- rbinom(n, 1, 0.7)
    df          <- data.frame(time = time, status = status, value = value)
    fit <- survival_km_median(df)                               # cut at median
    fit[c("cut", "n_high", "n_low", "logrank_p", "HR", "HR_low", "HR_high")]
    survival_km_median(df, cut = 0)$logrank_p          # cut at a fixed value
}
# ==============================================================================
# survival_km_optimal_cutpoint()  —  Kaplan-Meier stratification at the optimal
#                                                         cutpoint of a continuous marker
#
# Method : Choose the optimal dichotomization cutpoint of a continuous marker
#                  (e.g. gene expression) by maximally-selected rank statistics
#                  (survminer::surv_cutpoint, minprop = 0.3), split samples into
#                  High / Low, then compute the log-rank test (survival::survdiff) and
#                  the univariate Cox hazard ratio with 95% CI (survival::coxph).
#                  Returns numbers only  —  plotting is left to the caller.
# Input    : df    data.frame with columns: time (follow-up), status (1 = event,
#                         0 = censored), value (continuous marker).
# Output : list(cutpoint, data, n_high, n_low, logrank_p, HR, HR_low, HR_high,
#                  cox_p, median_high, median_low). Compute only  —  no plotting.
# Depends: survival, survminer (which uses maxstat).
# ==============================================================================

#' Kaplan-Meier stratification at an optimal cutpoint (compute only)
#'
#' @param df data.frame containing the time, status and marker columns.
#' @param time,status,value column names in \code{df} (defaults "time",
#'      "status", "value"). \code{status} must be 1 = event, 0 = censored.
#' @param minprop minimum group proportion for \code{surv_cutpoint} (default 0.3).
#' @return a list of the cutpoint, the annotated data, group sizes, the log-rank
#'      p-value, the Cox HR with 95% CI and p-value, and median survival per group.
survival_km_optimal_cutpoint <- function(df, time = "time", status = "status",
                                                                  value = "value", minprop = 0.3) {
    for (col in c(time, status, value))
        if (!col %in% names(df)) stop("column not found: ", col)

    d <- data.frame(time      = as.numeric(df[[time]]),
                                status = as.integer(df[[status]]),
                                value    = as.numeric(df[[value]]))
    d <- d[stats::complete.cases(d) & d$time > 0, , drop = FALSE]
    if (nrow(d) < 10) stop("need >= 10 complete samples with positive time.")
```

```r
  cut <- survminer::surv_cutpoint(d, time = "time", event = "status",
                                  variables = "value", minprop = minprop)
  cp   <- survminer::surv_categorize(cut)
  d$group <- factor(ifelse(tolower(cp$value) == "high", "High", "Low"),
                    levels = c("Low", "High"))

  sd <- survival::survdiff(survival::Surv(time, status) ~ group, data = d)
  logrank_p <- stats::pchisq(sd$chisq, df = length(sd$n) - 1, lower.tail = FALSE)

  cox <- survival::coxph(survival::Surv(time, status) ~ group, data = d)
  sm   <- summary(cox)
  fit <- survival::survfit(survival::Surv(time, status) ~ group, data = d)
  med <- summary(fit)$table[, "median"]

  list(
    cutpoint    = as.numeric(cut$cutpoint$cutpoint),
    data        = d,
    n_high      = sum(d$group == "High"),
    n_low       = sum(d$group == "Low"),
    logrank_p   = unname(logrank_p),
    HR          = unname(sm$conf.int[1, "exp(coef)"]),
    HR_low      = unname(sm$conf.int[1, "lower .95"]),
    HR_high     = unname(sm$conf.int[1, "upper .95"]),
    cox_p       = unname(sm$coefficients[1, "Pr(>|z|)"]),
    median_high = unname(med[grep("High", names(med))]),
    median_low  = unname(med[grep("Low",  names(med))])
  )
}

# ---- Example (simulated data; select this block and run) --------------------
if (FALSE) {
  set.seed(1)
  n      <- 200
  value  <- rnorm(n)
  time   <- rexp(n, rate = 0.1 * exp(0.8 * as.numeric(scale(value))))  # marker raises hazard
  status <- rbinom(n, 1, 0.7)
  df     <- data.frame(time = time, status = status, value = value)
  fit <- survival_km_optimal_cutpoint(df)
  fit[c("cutpoint", "n_high", "n_low", "logrank_p", "HR", "HR_low", "HR_high")]
}
# ============================================================================
# survival_km_quartile() — Kaplan-Meier stratification of a continuous marker
#                          into quartiles
#
# Method : Split a continuous marker at its 25th / 50th / 75th percentiles into
#          four groups (Q1-Q4), then compute the Kaplan-Meier fit
#          (survival::survfit) and the global log-rank test across the four
#          groups (survival::survdiff). Returns numbers only — plotting is left
#          to the caller.
# Input  : df   data.frame with columns: time (follow-up), status (1 = event,
#               0 = censored), value (continuous marker).
# Output : list(cutpoints, n_per_group, logrank_p, median_surv). Compute only —
#          no plotting.
# Depends: survival.
# ============================================================================
```

```r
#' Kaplan-Meier stratification into quartiles (compute only)
#'
#' @param df data.frame containing the time, status and marker columns.
#' @param time,status,value column names in \code{df} (defaults "time",
#'      "status", "value"). \code{status} must be 1 = event, 0 = censored.
#' @return a list with the three quartile cutpoints, the group sizes, the global
#'      log-rank p-value across the four groups, and median survival per quartile.
survival_km_quartile <- function(df, time = "time", status = "status",
                                 value = "value") {
  for (col in c(time, status, value))
    if (!col %in% names(df)) stop("column not found: ", col)

  d <- data.frame(time      = as.numeric(df[[time]]),
                  status = as.integer(df[[status]]),
                  value    = as.numeric(df[[value]]))
  d <- d[stats::complete.cases(d) & d$time > 0, , drop = FALSE]
  if (nrow(d) < 20) stop("need >= 20 complete samples with positive time.")

  qs <- stats::quantile(d$value, probs = c(0.25, 0.5, 0.75), names = FALSE)
  if (length(unique(qs)) < 3L)
    stop("marker has too many ties to form four distinct quartiles.")
  d$group <- cut(d$value, breaks = c(-Inf, qs, Inf),
                 labels = c("Q1", "Q2", "Q3", "Q4"), include.lowest = TRUE)

  sd <- survival::survdiff(survival::Surv(time, status) ~ group, data = d)
  logrank_p <- stats::pchisq(sd$chisq, df = length(sd$n) - 1, lower.tail = FALSE)

  fit <- survival::survfit(survival::Surv(time, status) ~ group, data = d)
  med <- summary(fit)$table[, "median"]

  list(
    cutpoints      = stats::setNames(qs, c("Q1|Q2", "Q2|Q3", "Q3|Q4")),
    n_per_group = table(d$group),
    logrank_p      = unname(logrank_p),
    median_surv = stats::setNames(unname(med), levels(d$group))
  )
}

# ---- Example (simulated data; select this block and run) --------------------
if (FALSE) {
  set.seed(1)
  n          <- 400
  value    <- rnorm(n)
  time      <- rexp(n, rate = 0.1 * exp(0.6 * as.numeric(scale(value))))
  status <- rbinom(n, 1, 0.7)
  df         <- data.frame(time = time, status = status, value = value)
  res <- survival_km_quartile(df)
  res[c("cutpoints", "n_per_group", "logrank_p")]
  res$median_surv
}
# ============================================================================
# survival_meta_hr()  —  Random-effects meta-analysis of hazard ratios
#
# Method : Pool per-cohort hazard ratios with a random-effects model
#                  (metafor::rma, REML). Effect sizes are log hazard ratios; when only
#                  HR and its 95% CI are given, the standard error is recovered from the
#                  CI width, se = (log(HR_high) - log(HR_low)) / (2 * z). Returns the
```

```
#                   pooled HR, heterogeneity statistics and per-study effects. Returns
#                   numbers only  –  plotting (e.g. a forest plot) is left to the caller.
# Input    : df    data.frame with one row per cohort and columns cohort, HR,
#                         HR_low, HR_high; or cohort plus pre-computed logHR and se columns.
# Output : list(pooled_HR, ci_low, ci_high, I2, tau2, p.value, per_study).
#                   Compute only  –  no plotting.
# Depends: metafor.
# ================================================================================

#' Random-effects meta-analysis of hazard ratios (compute only)
#'
#' @param df data.frame with one row per cohort.
#' @param cohort column name of the cohort labels (default "cohort").
#' @param HR,HR_low,HR_high column names of the hazard ratio and its 95\% CI
#'      (defaults "HR", "HR_low", "HR_high"); used when \code{logHR}/\code{se} are
#'      not supplied.
#' @param logHR,se optional column names of a pre-computed log hazard ratio and
#'      its standard error; when both are given they take precedence over
#'      \code{HR}/\code{HR_low}/\code{HR_high}.
#' @param level confidence level of the supplied CI (default 0.95).
#' @return a list with the pooled HR and its CI, heterogeneity (I2, tau2), the
#'      test p-value, and a per-study data.frame (HR, CI, weight).
survival_meta_hr <- function(df, cohort = "cohort", HR = "HR",
                                               HR_low = "HR_low", HR_high = "HR_high",
                                               logHR = NULL, se = NULL, level = 0.95) {
   if (!cohort %in% names(df)) stop("column not found: ", cohort)
   z <- stats::qnorm(1 - (1 - level) / 2)

   if (!is.null(logHR) && !is.null(se)) {
      for (col in c(logHR, se))
         if (!col %in% names(df)) stop("column not found: ", col)
      yi    <- as.numeric(df[[logHR]])
      sei <- as.numeric(df[[se]])
   } else {
      for (col in c(HR, HR_low, HR_high))
         if (!col %in% names(df)) stop("column not found: ", col)
      yi    <- log(as.numeric(df[[HR]]))
      sei <- (log(as.numeric(df[[HR_high]])) - log(as.numeric(df[[HR_low]]))) / (2 * z)
   }

   keep <- is.finite(yi) & is.finite(sei) & sei > 0
   if (sum(keep) < 2L) stop("need >= 2 cohorts with valid effect sizes.")
   labels <- as.character(df[[cohort]])[keep]
   yi <- yi[keep]; sei <- sei[keep]

   m <- metafor::rma(yi = yi, sei = sei, method = "REML", slab = labels)

   per_study <- data.frame(
      cohort    = labels,
      HR           = exp(yi),
      HR_low    = exp(yi - z * sei),
      HR_high = exp(yi + z * sei),
      weight    = as.numeric(stats::weights(m)),
      row.names = NULL, check.names = FALSE)

   list(
      pooled_HR = unname(exp(as.numeric(m$beta[1]))),
```

```
    ci_low        = unname(exp(m$ci.lb)),
    ci_high      = unname(exp(m$ci.ub)),
    I2               = unname(m$I2),
    tau2           = unname(m$tau2),
    p.value      = unname(m$pval),
    per_study = per_study
  )
}

# ---- Example (simulated data; select this block and run) --------------------
if (FALSE) {
  set.seed(1)
  k           <- 8
  logHR     <- rnorm(k, mean = 0.5, sd = 0.2)          # true pooled logHR ~ 0.5
  se          <- runif(k, 0.1, 0.3)
  obs         <- logHR + rnorm(k, 0, se)
  z             <- qnorm(0.975)
  df <- data.frame(cohort    = paste0("Cohort", 1:k),
                             HR           = exp(obs),
                             HR_low    = exp(obs - z * se),
                             HR_high = exp(obs + z * se))
  res <- survival_meta_hr(df)
  res[c("pooled_HR", "ci_low", "ci_high", "I2", "tau2", "p.value")]
  res$per_study
}
# ============================================================================
# survival_multicox()  —  Multivariate Cox regression
#
# Method : Fit a single multivariate Cox proportional-hazards model
#                (survival::coxph) regressing survival on all supplied covariates
#                jointly, and tabulate each term's hazard ratio with 95% CI, Wald z
#                and p-value in a forest-ready data.frame. Returns a table only  —
#                plotting is left to the caller.
# Input    : df    data.frame with columns time (follow-up), status (1 = event,
#                       0 = censored) and one or more covariate columns (numeric or
#                       factor).
# Output : data.frame(term, HR, HR_low, HR_high, z, p.value), one row per model
#                term. Compute only  —  no plotting.
# Depends: survival.
# ============================================================================

#' Multivariate Cox regression (compute only)
#'
#' @param df data.frame with the time, status and covariate columns.
#' @param time,status column names in \code{df} (defaults "time", "status").
#'     \code{status} must be 1 = event, 0 = censored.
#' @param covariates character vector of covariate column names to include;
#'     defaults to every column of \code{df} other than \code{time} and
#'     \code{status}.
#' @return a forest-ready data.frame, one row per model term.
survival_multicox <- function(df, time = "time", status = "status",
                                              covariates = NULL) {
  for (col in c(time, status))
    if (!col %in% names(df)) stop("column not found: ", col)
  if (is.null(covariates))
    covariates <- setdiff(names(df), c(time, status))
  if (length(covariates) < 1L) stop("no covariate columns found.")
```

```
  miss <- setdiff(covariates, names(df))
  if (length(miss)) stop("covariate not found: ", paste(miss, collapse = ", "))

  d <- df[, c(time, status, covariates), drop = FALSE]
  names(d)[1:2] <- c("time", "status")
  d$time      <- as.numeric(d$time)
  d$status <- as.integer(d$status)
  d <- d[stats::complete.cases(d) & d$time > 0, , drop = FALSE]
  if (nrow(d) < 10) stop("need >= 10 complete samples with positive time.")

  rhs    <- paste(sprintf("`%s`", covariates), collapse = " + ")
  form <- stats::as.formula(paste("survival::Surv(time, status) ~", rhs))
  cox    <- survival::coxph(form, data = d)
  sm      <- summary(cox)

  data.frame(
    term        = rownames(sm$coefficients),
    HR           = unname(sm$conf.int[, "exp(coef)"]),
    HR_low    = unname(sm$conf.int[, "lower .95"]),
    HR_high = unname(sm$conf.int[, "upper .95"]),
    z             = unname(sm$coefficients[, "z"]),
    p.value = unname(sm$coefficients[, "Pr(>|z|)"]),
    row.names = NULL, check.names = FALSE
  )
}

# ---- Example (simulated data; select this block and run) -------------------
if (FALSE) {
  set.seed(1)
  n           <- 300
  age        <- round(rnorm(n, 60, 10))
  sex        <- factor(sample(c("F", "M"), n, replace = TRUE))
  stage    <- factor(sample(c("I", "II", "III"), n, replace = TRUE))
  score    <- rnorm(n)
  risk      <- 0.03 * (age - 60) + 0.8 * score
  time      <- rexp(n, rate = 0.05 * exp(risk))
  status <- rbinom(n, 1, 0.7)
  df <- data.frame(time = time, status = status,
                                age = age, sex = sex, stage = stage, score = score)
  survival_multicox(df)
}
# ============================================================================
# survival_two_gene_combined()  —  Survival by a two-gene combined score
#
# Method : Combine two genes into one score by z-scoring each gene across
#                  samples and summing the two z-scores, match the score to survival
#                  endpoints by sample id, dichotomize at the median (or a user cut
#                  value), and compute the log-rank test (survival::survdiff) and the
#                  univariate Cox hazard ratio with 95% CI (survival::coxph). Returns
#                  numbers only  —  plotting is left to the caller.
# Input    : expr        numeric matrix, genes in rows (rownames = gene symbols),
#                               samples in columns (colnames = sample ids).
#                  gene1, gene2     two gene symbols present in rownames(expr).
#                  surv        data.frame with a sample-id column plus time (follow-up) and
#                               status (1 = event, 0 = censored).
# Output : list(cut, n_high, n_low, logrank_p, HR, HR_low, HR_high, cox_p,
#                  median_high, median_low). Compute only  —  no plotting.
```

```
# Depends: survival.
# ==========================================================================

#' Survival by a two-gene combined z-score (compute only)
#'
#' @param expr numeric matrix (genes x samples); rownames are gene symbols,
#'     colnames are sample ids.
#' @param gene1,gene2 the two gene symbols to combine.
#' @param surv data.frame of survival endpoints keyed by sample id.
#' @param sample,time,status column names in \code{surv} (defaults "sample",
#'     "time", "status"). \code{status} must be 1 = event, 0 = censored.
#' @param cut either "median" (dichotomize the combined score at its median,
#'     the default) or a single numeric cut value; scores above it are "High".
#'     Pass a numeric to use an externally determined (e.g. optimal) cutpoint.
#' @return a list with the cut value used, group sizes, the log-rank p-value,
#'     the Cox HR with 95% CI and p-value, and median survival per group.
survival_two_gene_combined <- function(expr, gene1, gene2, surv,
                                       sample = "sample", time = "time",
                                       status = "status", cut = "median") {
  expr <- as.matrix(expr)
  for (g in c(gene1, gene2))
    if (!g %in% rownames(expr)) stop("gene not found in expr: ", g)
  for (col in c(sample, time, status))
    if (!col %in% names(surv)) stop("column not found: ", col)

  z1 <- as.numeric(scale(as.numeric(expr[gene1, ])))     # per-gene z-score
  z2 <- as.numeric(scale(as.numeric(expr[gene2, ])))
  sc <- data.frame(sample = colnames(expr), score = z1 + z2,
                   stringsAsFactors = FALSE)
  sv <- data.frame(sample = as.character(surv[[sample]]),
                   time   = as.numeric(surv[[time]]),
                   status = as.integer(surv[[status]]),
                   stringsAsFactors = FALSE)

  d <- merge(sc, sv, by = "sample")
  d <- d[stats::complete.cases(d) & d$time > 0, , drop = FALSE]
  if (nrow(d) < 10) stop("need >= 10 matched samples with positive time.")

  cp <- if (is.numeric(cut)) cut[1L] else stats::median(d$score)
  d$group <- factor(ifelse(d$score > cp, "High", "Low"), levels = c("Low", "High"))
  if (nlevels(droplevels(d$group)) < 2L)
    stop("the cut value does not split the samples into two groups.")

  sd <- survival::survdiff(survival::Surv(time, status) ~ group, data = d)
  logrank_p <- stats::pchisq(sd$chisq, df = length(sd$n) - 1, lower.tail = FALSE)

  cox <- survival::coxph(survival::Surv(time, status) ~ group, data = d)
  sm  <- summary(cox)
  fit <- survival::survfit(survival::Surv(time, status) ~ group, data = d)
  med <- summary(fit)$table[, "median"]

  list(
    cut       = as.numeric(cp),
    n_high    = sum(d$group == "High"),
    n_low     = sum(d$group == "Low"),
    logrank_p = unname(logrank_p),
    HR        = unname(sm$conf.int[1, "exp(coef)"]),
```

```
      HR_low          = unname(sm$conf.int[1, "lower .95"]),
      HR_high         = unname(sm$conf.int[1, "upper .95"]),
      cox_p           = unname(sm$coefficients[1, "Pr(>|z|)"]),
      median_high = unname(med[grep("High", names(med))]),
      median_low    = unname(med[grep("Low",    names(med))])
  )
}

# ---- Example (simulated data; select this block and run) --------------------
if (FALSE) {
  set.seed(1)
  n        <- 200
  genes <- 100
  expr   <- matrix(rnorm(genes * n), nrow = genes,
                            dimnames = list(paste0("Gene", 1:genes), paste0("S", 1:n)))
  risk     <- as.numeric(scale(expr["Gene1", ] + expr["Gene2", ]))
  time     <- rexp(n, rate = 0.1 * exp(0.8 * risk))
  status <- rbinom(n, 1, 0.7)
  surv     <- data.frame(sample = paste0("S", 1:n), time = time, status = status)
  fit <- survival_two_gene_combined(expr, "Gene1", "Gene2", surv)
  fit[c("cut", "n_high", "n_low", "logrank_p", "HR", "HR_low", "HR_high")]
}
# ============================================================================
# survival_unicox()  —  Univariate Cox regression, one feature at a time
#
# Method : Fit a separate univariate Cox proportional-hazards model
#               (survival::coxph) for each feature (e.g. each gene), regressing
#               survival on that single feature, and tabulate the hazard ratio with
#               95% CI, Wald z, p-value and Benjamini-Hochberg FDR. Returns a table
#               only  —  plotting is left to the caller.
# Input    : features    matrix / data.frame, samples in rows, features in columns
#                              (colnames = feature names).
#               surv          data.frame with columns time (follow-up) and status
#                              (1 = event, 0 = censored); its rows correspond to the rows
#                              of `features`, in the same order.
# Output : data.frame(feature, HR, HR_low, HR_high, z, p.value, p.adj) ordered
#               by p.value. Compute only  —  no plotting.
# Depends: survival.
# ============================================================================

#' Univariate Cox regression per feature (compute only)
#'
#' @param features matrix or data.frame with samples in rows and features in
#'     columns; \code{colnames} are used as feature names.
#' @param surv data.frame of survival endpoints; rows correspond to the rows of
#'     \code{features} (same samples, same order).
#' @param time,status column names in \code{surv} (defaults "time", "status").
#'     \code{status} must be 1 = event, 0 = censored.
#' @param p_adjust multiple-testing method for \code{\link[stats]{p.adjust}}
#'     (default "BH").
#' @param min_samples minimum complete samples required to fit a feature
#'     (default 10).
#' @return data.frame, one row per feature, ordered by \code{p.value}.
survival_unicox <- function(features, surv, time = "time", status = "status",
                                          p_adjust = "BH", min_samples = 10) {
  features <- as.data.frame(features, stringsAsFactors = FALSE)
  if (is.null(colnames(features)))
```

```
    colnames(features) <- paste0("V", seq_len(ncol(features)))
  for (col in c(time, status))
    if (!col %in% names(surv)) stop("column not found: ", col)
  if (nrow(features) != nrow(surv))
    stop("features and surv must have the same number of rows (samples).")

  tt <- as.numeric(surv[[time]])
  ss <- as.integer(surv[[status]])

  stat <- t(vapply(seq_len(ncol(features)), function(j) {
    dd <- data.frame(time = tt, status = ss, x = as.numeric(features[[j]]))
    dd <- dd[stats::complete.cases(dd) & dd$time > 0, , drop = FALSE]
    if (nrow(dd) < min_samples || stats::sd(dd$x) == 0)
      return(c(HR = NA_real_, lo = NA_real_, hi = NA_real_,
               z = NA_real_, p = NA_real_))
    cox <- tryCatch(
      suppressWarnings(survival::coxph(survival::Surv(time, status) ~ x, data = dd)),
      error = function(e) NULL)
    if (is.null(cox))
      return(c(HR = NA_real_, lo = NA_real_, hi = NA_real_,
               z = NA_real_, p = NA_real_))
    sm <- summary(cox)
    c(HR = unname(sm$conf.int[1, "exp(coef)"]),
      lo = unname(sm$conf.int[1, "lower .95"]),
      hi = unname(sm$conf.int[1, "upper .95"]),
      z  = unname(sm$coefficients[1, "z"]),
      p  = unname(sm$coefficients[1, "Pr(>|z|)"]))
  }, numeric(5)))

  out <- data.frame(feature = colnames(features), stat,
                    p.adj = stats::p.adjust(stat[, "p"], method = p_adjust),
                    row.names = NULL, check.names = FALSE)
  colnames(out) <- c("feature", "HR", "HR_low", "HR_high", "z", "p.value", "p.adj")
  out <- out[order(out$p.value), ]
  rownames(out) <- NULL
  out
}

# ---- Example (simulated data; select this block and run) --------------------
if (FALSE) {
  set.seed(1)
  n        <- 200
  p        <- 50
  features <- matrix(rnorm(n * p), nrow = n,
                     dimnames = list(paste0("S", 1:n), paste0("Gene", 1:p)))
  risk     <- as.numeric(scale(features[, "Gene1"]))          # one true feature
  time     <- rexp(n, rate = 0.1 * exp(0.9 * risk))
  status   <- rbinom(n, 1, 0.7)
  surv     <- data.frame(time = time, status = status)
  res <- survival_unicox(features, surv)
  head(res)
}
# ============================================================================
# sc_deg_volcano()  —  Volcano plot from a marker / DE table
#
# Method : A volcano plot built directly with ggplot2 from a differential-
#          expression table (e.g. the output of Seurat::FindMarkers): each gene is
```

```
#                  placed at (log2 fold change, -log10 adjusted p) and coloured Up / Down /
#                  n.s. by fold-change and significance thresholds; the strongest genes are
#                  text-labelled. Zero adjusted p-values are floored to keep -log10 finite.
# Input    : markers    a data.frame with a log2FC column and an adjusted-p column;
#                  gene labels come from `gene_col`, a `gene` column, or the row names.
# Output : a ggplot object (the caller prints it or saves with ggplot2::ggsave).
# Depends: ggplot2 (utils, base R).
# ============================================================================

#' Volcano plot from a marker / DE table
#'
#' @param markers a data.frame of differential-expression results.
#' @param lfc_col name of the log2 fold-change column (default "avg_log2FC").
#' @param padj_col name of the adjusted-p column (default "p_val_adj").
#' @param gene_col optional column holding gene labels; falls back to a "gene"
#'      column, then to \code{rownames(markers)}.
#' @param lfc_threshold absolute log2FC cutoff for Up / Down (default 1).
#' @param padj_threshold adjusted-p cutoff for significance (default 0.05).
#' @param top_n number of most-significant genes to label (default 10).
#' @param palette named colours for "Down", "ns", "Up".
#' @return a \code{ggplot} object.
sc_deg_volcano <- function(markers, lfc_col = "avg_log2FC",
                           padj_col = "p_val_adj", gene_col = NULL,
                           lfc_threshold = 1, padj_threshold = 0.05,
                           top_n = 10,
                           palette = c(Down = "#3B7FB6", ns = "grey75",
                                       Up = "#C0392B")) {
  for (col in c(lfc_col, padj_col))
    if (!col %in% colnames(markers))
      stop("column '", col, "' not found in `markers`.")
  genes <- if (!is.null(gene_col)) markers[[gene_col]]
           else if ("gene" %in% colnames(markers)) markers[["gene"]]
           else rownames(markers)

  lfc    <- markers[[lfc_col]]
  padj <- markers[[padj_col]]
  floor_p <- suppressWarnings(min(padj[padj > 0], na.rm = TRUE))    # keep finite
  if (!is.finite(floor_p)) floor_p <- 1e-300
  padj[is.na(padj) | padj == 0] <- floor_p * 0.1
  cls <- ifelse(padj < padj_threshold & lfc >=    lfc_threshold, "Up",
         ifelse(padj < padj_threshold & lfc <= -lfc_threshold, "Down", "ns"))

  d <- data.frame(gene = genes, lfc = lfc, neglogp = -log10(padj),
                  cls = factor(cls, levels = c("Down", "ns", "Up")))
  sig <- d[d$cls != "ns", ]
  lab <- utils::head(sig[order(-sig$neglogp), ], top_n)

  ggplot2::ggplot(d, ggplot2::aes(x = lfc, y = neglogp, colour = cls)) +
    ggplot2::geom_point(size = 1, alpha = 0.8) +
    ggplot2::geom_vline(xintercept = c(-lfc_threshold, lfc_threshold),
                        linetype = "dashed", colour = "grey60") +
    ggplot2::geom_hline(yintercept = -log10(padj_threshold),
                        linetype = "dashed", colour = "grey60") +
    ggplot2::geom_text(data = lab, ggplot2::aes(label = gene), size = 3,
                       vjust = -0.6, show.legend = FALSE) +
    ggplot2::scale_colour_manual(values = palette, drop = FALSE) +
    ggplot2::labs(x = "log2 fold change", y = "-log10(adjusted p)",
```

```
                                    colour = NULL) +
        ggplot2::theme_classic(base_size = 12)
}

# ---- Example (simulated marker table; select this block and run) ------------
if (FALSE) {
    set.seed(1)
    markers <- data.frame(
        gene            = paste0("Gene", 1:200),
        avg_log2FC = rnorm(200, 0, 1.2),
        p_val_adj    = 10^(-rexp(200, 0.5)))
    markers$avg_log2FC[1:6] <- c(3, -3, 2.6, -2.8, 3.2, -2.4)      # strong hits
    markers$p_val_adj[1:6]    <- 1e-30
    p <- sc_deg_volcano(markers)
    inherits(p, "ggplot")
}
# ==============================================================================
# sc_density_umap()  —  Expression-weighted density on a UMAP embedding
#
# Method : Nebulosa::plot_density draws a kernel-density estimate of a feature's
#                 expression over the embedding, weighting each cell by its expression.
#                 The smoothed field is easier to read than a per-cell FeaturePlot when
#                 signal is sparse or dropout-heavy. A thin wrapper that returns the plot
#                 object. (For SeuratObject >= 5.3, Nebulosa's Seurat method calls the
#                 now-defunct FetchData(slot=); this wrapper transparently routes through
#                 Nebulosa's SingleCellExperiment method, which is unaffected.)
# Input    : seurat_obj    a Seurat object with the reduction computed; features    a
#                 character vector of genes.
# Output : a ggplot object (single feature) or a patchwork of panels (several).
# Depends: Nebulosa (Seurat for the compatibility fallback).
# ==============================================================================

#' Expression-weighted density on a UMAP embedding
#'
#' @param seurat_obj a Seurat object.
#' @param features character vector of genes to draw density for.
#' @param reduction name of the reduction to plot (default "umap").
#' @param joint if TRUE (with several features) add a joint-density panel.
#' @param ... further arguments passed to \code{\link[Nebulosa]{plot_density}}.
#' @return a \code{ggplot} / \code{patchwork} object.
sc_density_umap <- function(seurat_obj, features, reduction = "umap",
                                             joint = FALSE, ...) {
    tryCatch(
        Nebulosa::plot_density(seurat_obj, features = features,
                                           reduction = reduction, joint = joint, ...),
        defunctError = function(e)
            ## Nebulosa's Seurat method reads expression via FetchData(slot=), which is
            ## defunct in SeuratObject >= 5.3; its SingleCellExperiment method is not,
            ## so route through a lightweight SCE conversion when the defunct is hit.
            Nebulosa::plot_density(Seurat::as.SingleCellExperiment(seurat_obj),
                                               features = features, joint = joint, ...)
    )
}

# ---- Example (simulated: build a tiny Seurat object) -----------------------
if (FALSE) {
    library(Seurat)
```

```r
  set.seed(1)
  counts <- matrix(rpois(300 * 80, 4), nrow = 300,
                   dimnames = list(paste0("Gene", 1:300), paste0("Cell", 1:80)))
  so   <- CreateSeuratObject(counts)
  so   <- NormalizeData(so, verbose = FALSE)
  emb <- matrix(rnorm(80 * 2), 80, 2,
                dimnames = list(colnames(so), c("UMAP_1", "UMAP_2")))
  so[["umap"]] <- CreateDimReducObject(embeddings = emb, key = "UMAP_",
                                       assay = "RNA")
  p <- sc_density_umap(so, features = "Gene1")
  inherits(p, "ggplot") || inherits(p, "patchwork")
}
# ==============================================================================
# sc_feature_umap()  —  Feature expression on a UMAP embedding
#
# Method : Seurat::FeaturePlot colours cells on a 2-D embedding (default "umap")
#          by the expression of one or more features (genes or meta.data columns),
#          drawing high-expressing cells on top by default. A thin wrapper that
#          returns the plot object for the caller to arrange or save.
# Input  : seurat_obj   a Seurat object with the reduction computed; features   a
#          character vector of genes or meta.data columns.
# Output : a ggplot object (single feature) or a patchwork of panels (several).
# Depends: Seurat.
# ==============================================================================

#' Feature expression on a UMAP embedding
#'
#' @param seurat_obj a Seurat object.
#' @param features character vector of genes or meta.data columns to colour by.
#' @param reduction name of the reduction to plot (default "umap").
#' @param order draw high-expressing cells on top (default TRUE).
#' @param ... further arguments passed to \code{\link[Seurat]{FeaturePlot}}.
#' @return a \code{ggplot} / \code{patchwork} object.
sc_feature_umap <- function(seurat_obj, features, reduction = "umap",
                            order = TRUE, ...) {
  Seurat::FeaturePlot(seurat_obj, features = features, reduction = reduction,
                      order = order, ...)
}

# ---- Example (simulated: build a tiny Seurat object) -----------------------
if (FALSE) {
  library(Seurat)
  set.seed(1)
  counts <- matrix(rpois(300 * 80, 4), nrow = 300,
                   dimnames = list(paste0("Gene", 1:300), paste0("Cell", 1:80)))
  so   <- CreateSeuratObject(counts)
  so   <- NormalizeData(so, verbose = FALSE)
  emb <- matrix(rnorm(80 * 2), 80, 2,
                dimnames = list(colnames(so), c("UMAP_1", "UMAP_2")))
  so[["umap"]] <- CreateDimReducObject(embeddings = emb, key = "UMAP_",
                                       assay = "RNA")
  p <- sc_feature_umap(so, features = "Gene1")
  inherits(p, "ggplot") || inherits(p, "patchwork")
}
# ==============================================================================
# sc_find_markers()  —  Cluster / group marker genes (Seurat)
#
```

```r
# Method : Differential expression between groups of cells. With `ident_1` (and an
#          optional `ident_2`), Seurat::FindMarkers compares those two identity
#          groups; with `ident_1 = NULL`, Seurat::FindAllMarkers tests every
#          cluster against the rest. Defaults use the Wilcoxon rank-sum test on
#          log-normalized data, keeping genes with |log2FC| >= logfc_threshold that
#          are detected in >= min_pct of either group; p-values are BH-adjusted.
# Input  : seurat_obj  a Seurat object with normalized data; group_by  optional
#          meta.data column used to set cell identities before testing.
# Output : data.frame of markers. FindMarkers gains a leading `gene` column;
#          FindAllMarkers additionally carries a `cluster` column.
# Depends: Seurat, SeuratObject.
# ==============================================================================

#' Cluster / group marker genes (Seurat)
#'
#' @param seurat_obj a Seurat object with normalized data.
#' @param ident_1 identity to test; \code{NULL} runs FindAllMarkers over all
#'   identities.
#' @param ident_2 comparison identity for FindMarkers; \code{NULL} = all other
#'   cells.
#' @param group_by meta.data column to use as cell identity; \code{NULL} keeps the
#'   object's active identities.
#' @param test_use test for \code{\link[Seurat]{FindMarkers}} (default "wilcox").
#' @param logfc_threshold minimum absolute log2 fold change (default 0.25).
#' @param min_pct minimum detection fraction in either group (default 0.1).
#' @param only_pos keep only up-regulated markers (default FALSE).
#' @return a data.frame of differential-expression results.
sc_find_markers <- function(seurat_obj, ident_1 = NULL, ident_2 = NULL,
                            group_by = NULL, test_use = "wilcox",
                            logfc_threshold = 0.25, min_pct = 0.1,
                            only_pos = FALSE) {
  if (!is.null(group_by))
    SeuratObject::Idents(seurat_obj) <- seurat_obj[[group_by, drop = TRUE]]

  if (is.null(ident_1)) {
    Seurat::FindAllMarkers(seurat_obj, test.use = test_use,
                           logfc.threshold = logfc_threshold, min.pct = min_pct,
                           only.pos = only_pos, verbose = FALSE)
  } else {
    res <- Seurat::FindMarkers(seurat_obj, ident.1 = ident_1, ident.2 = ident_2,
                               test.use = test_use,
                               logfc.threshold = logfc_threshold,
                               min.pct = min_pct, only.pos = only_pos,
                               verbose = FALSE)
    data.frame(gene = rownames(res), res, row.names = NULL,
               check.names = FALSE)
  }
}

# ---- Example (simulated: build a tiny Seurat object) -----------------------
if (FALSE) {
  library(Seurat)
  set.seed(1)
  counts <- matrix(rpois(300 * 80, 4), nrow = 300,
                   dimnames = list(paste0("Gene", 1:300), paste0("Cell", 1:80)))
  counts[1:10, 1:20] <- counts[1:10, 1:20] + 15        # spike group-T markers
  so <- CreateSeuratObject(counts)
```

```r
  so <- NormalizeData(so, verbose = FALSE)
  so$cell_type <- rep(c("T", "B", "Myeloid", "NK"), each = 20)
  res <- sc_find_markers(so, ident_1 = "T", ident_2 = "B", group_by = "cell_type")
  head(res)
}
# ==============================================================================
# sc_geneset_score_aucell()  —  Gene-set activity per cell by AUCell
#
# Method : AUCell scores each cell for each gene set without choosing a threshold.
#          It builds a per-cell ranking of genes (AUCell_buildRankings) and takes
#          the area under the recovery curve (AUCell_calcAUC) of each set within
#          the top-ranked genes, so a score reflects how concentrated a set is
#          near the top of that cell's transcriptome. Being rank-based, it is
#          robust to cell-to-cell differences in detection depth. One column per
#          set is attached to meta.data.
# Input  : seurat_obj  a Seurat object; gene_sets  a named list() of character
#          vectors of gene symbols.
# Output : the Seurat object with one AUC column per gene set in meta.data
#          (store = TRUE), or the AUC matrix (sets x cells) when store = FALSE.
# Depends: AUCell, SeuratObject.
# ==============================================================================

#' Gene-set activity per cell by AUCell
#'
#' @param seurat_obj a Seurat object.
#' @param gene_sets named \code{list()} of gene-symbol character vectors.
#' @param assay assay to score (default "RNA").
#' @param layer assay layer to rank; "counts" is typical for AUCell (default).
#' @param auc_max_rank size of the top-ranked gene neighbourhood used for the AUC;
#'    \code{NULL} defaults to 5% of the ranked genes.
#' @param store if TRUE, attach AUC columns to meta.data and return the Seurat
#'    object; if FALSE, return the AUC matrix (sets x cells).
#' @return a Seurat object (store = TRUE) or a numeric matrix (store = FALSE).
sc_geneset_score_aucell <- function(seurat_obj, gene_sets, assay = "RNA",
                                    layer = "counts", auc_max_rank = NULL,
                                    store = TRUE) {
  if (is.null(names(gene_sets)) || any(names(gene_sets) == ""))
    stop("`gene_sets` must be a fully named list.")
  mat <- as.matrix(SeuratObject::GetAssayData(seurat_obj, assay = assay,
                                              layer = layer))
  rankings <- AUCell::AUCell_buildRankings(mat, plotStats = FALSE,
                                           verbose = FALSE)
  if (is.null(auc_max_rank))
    auc_max_rank <- ceiling(0.05 * nrow(rankings))
  auc <- AUCell::AUCell_calcAUC(gene_sets, rankings, verbose = FALSE,
                                aucMaxRank = auc_max_rank)
  auc_mat <- AUCell::getAUC(auc)                          # sets x cells
  if (!isTRUE(store)) return(auc_mat)

  SeuratObject::AddMetaData(seurat_obj, metadata = as.data.frame(t(auc_mat)))
}

# ---- Example (simulated: build a tiny Seurat object) -----------------------
if (FALSE) {
  library(Seurat)
  set.seed(1)
  counts <- matrix(rpois(300 * 80, 4), nrow = 300,
```

```
                              dimnames = list(paste0("Gene", 1:300), paste0("Cell", 1:80)))
  so      <- CreateSeuratObject(counts)
  sets <- list(SetA = paste0("Gene", 1:12), SetB = paste0("Gene", 13:24))
  so      <- sc_geneset_score_aucell(so, sets)
  head(so[[]][, c("SetA", "SetB")])
}
# ==============================================================================
# sc_geneset_score_singscore()  —  Gene-set score per cell by singscore
#
# Method : singscore ranks each cell's transcriptome (rankGenes) and scores each
#            gene set with simpleScore, returning a normalized mean-rank score in
#            [-0.5, 0.5]. The score is undirected, so a coherently up-regulated set
#            is passed as the "up" set; the per-cell ranking makes scores comparable
#            across cells of differing depth. One "<set>_singscore" column is added
#            to meta.data per set.
# Input    : seurat_obj    a Seurat object with normalized data; gene_sets    a named
#            list() of character vectors of gene symbols.
# Output : the Seurat object with one "<set>_singscore" column per set.
# Depends: singscore, SeuratObject.
# ==============================================================================

#' Gene-set score per cell by singscore
#'
#' @param seurat_obj a Seurat object.
#' @param gene_sets named \code{list()} of gene-symbol character vectors.
#' @param assay assay to score (default "RNA").
#' @param layer assay layer to rank (default "data", i.e. normalized).
#' @return the Seurat object with singscore columns in meta.data.
sc_geneset_score_singscore <- function(seurat_obj, gene_sets, assay = "RNA",
                                       layer = "data") {
  if (is.null(names(gene_sets)) || any(names(gene_sets) == ""))
    stop("`gene_sets` must be a fully named list.")
  mat <- as.matrix(SeuratObject::GetAssayData(seurat_obj, assay = assay,
                                              layer = layer))
  ranks <- singscore::rankGenes(mat)
  scores <- vapply(gene_sets, function(genes) {
    genes <- intersect(genes, rownames(mat))
    if (!length(genes)) return(rep(NA_real_, ncol(mat)))
    singscore::simpleScore(ranks, upSet = genes)$TotalScore
  }, numeric(ncol(mat)))
  colnames(scores) <- paste0(names(gene_sets), "_singscore")
  rownames(scores) <- colnames(mat)
  SeuratObject::AddMetaData(seurat_obj, metadata = as.data.frame(scores))
}

# ---- Example (simulated: build a tiny Seurat object) -----------------------
if (FALSE) {
  library(Seurat)
  set.seed(1)
  counts <- matrix(rpois(300 * 80, 4), nrow = 300,
                              dimnames = list(paste0("Gene", 1:300), paste0("Cell", 1:80)))
  so      <- CreateSeuratObject(counts)
  so      <- NormalizeData(so, verbose = FALSE)
  sets <- list(SetA = paste0("Gene", 1:12), SetB = paste0("Gene", 13:24))
  so      <- sc_geneset_score_singscore(so, sets)
  head(so[[]][, c("SetA_singscore", "SetB_singscore")])
}
```

```
# ==============================================================================
# sc_geneset_score_ucell()  –  Gene-set activity per cell by UCell
#
# Method : UCell scores each cell independently from a Mann-Whitney U statistic on
#          within-cell gene ranks (AddModuleScore_UCell). Because scoring uses only
#          a cell's own ranks, a score does not change when other cells are added
#          or removed, which makes UCell stable across dataset composition and
#          batch integration. One "<set>_UCell" column is added to meta.data per
#          gene set.
# Input  : seurat_obj   a Seurat object; gene_sets   a named list() of character
#          vectors of gene symbols.
# Output : the Seurat object with one "<set>_UCell" column per set in meta.data.
# Depends: UCell.
# ==============================================================================

#' Gene-set activity per cell by UCell
#'
#' @param seurat_obj a Seurat object with normalized data.
#' @param gene_sets named \code{list()} of gene-symbol character vectors.
#' @param assay assay to score; \code{NULL} uses the default assay.
#' @param maxRank maximum rank UCell considers before a gene is treated as not
#'   detected (default 1500).
#' @return the Seurat object with UCell score columns in meta.data.
sc_geneset_score_ucell <- function(seurat_obj, gene_sets, assay = NULL,
                                   maxRank = 1500) {
  if (is.null(names(gene_sets)) || any(names(gene_sets) == ""))
    stop("`gene_sets` must be a fully named list.")
  UCell::AddModuleScore_UCell(seurat_obj, features = gene_sets,
                              assay = assay, maxRank = maxRank)
}

# ---- Example (simulated: build a tiny Seurat object) -----------------------
if (FALSE) {
  library(Seurat)
  set.seed(1)
  counts <- matrix(rpois(300 * 80, 4), nrow = 300,
                   dimnames = list(paste0("Gene", 1:300), paste0("Cell", 1:80)))
  so   <- CreateSeuratObject(counts)
  so   <- NormalizeData(so, verbose = FALSE)
  sets <- list(SetA = paste0("Gene", 1:12), SetB = paste0("Gene", 13:24))
  so   <- sc_geneset_score_ucell(so, sets)
  head(so[[]][, c("SetA_UCell", "SetB_UCell")])
}
# ==============================================================================
# sc_marker_dotplot()  –  Marker dot plot across groups
#
# Method : Seurat::DotPlot summarizes a set of markers across groups with two
#          channels per gene x group: dot size = fraction of cells expressing, dot
#          colour = scaled average expression. It is the standard compact view for
#          annotating clusters from known markers. A thin wrapper that rotates the
#          x-axis labels (RotatedAxis) and returns the plot.
# Input  : seurat_obj   a Seurat object; features   a character vector (or named
#          list() for gene grouping); group_by   optional grouping column.
# Output : a ggplot object (the caller prints it or saves with ggplot2::ggsave).
# Depends: Seurat.
# ==============================================================================
```

```r
#' Marker dot plot across groups
#'
#' @param seurat_obj a Seurat object.
#' @param features character vector of genes, or a named \code{list()} to split
#'     the axis into gene groups.
#' @param group_by meta.data column to group by; \code{NULL} uses active
#'     identities.
#' @param rotate_axis rotate x-axis tick labels for readability (default TRUE).
#' @param ... further arguments passed to \code{\link[Seurat]{DotPlot}}.
#' @return a \code{ggplot} object.
sc_marker_dotplot <- function(seurat_obj, features, group_by = NULL,
                              rotate_axis = TRUE, ...) {
  p <- Seurat::DotPlot(seurat_obj, features = features, group.by = group_by, ...)
  if (isTRUE(rotate_axis)) p <- p + Seurat::RotatedAxis()
  p
}

# ---- Example (simulated: build a tiny Seurat object) -----------------------
if (FALSE) {
  library(Seurat)
  set.seed(1)
  counts <- matrix(rpois(300 * 80, 4), nrow = 300,
                   dimnames = list(paste0("Gene", 1:300), paste0("Cell", 1:80)))
  so <- CreateSeuratObject(counts)
  so <- NormalizeData(so, verbose = FALSE)
  so$cell_type <- rep(c("T", "B", "Myeloid", "NK"), each = 20)
  p <- sc_marker_dotplot(so, features = c("Gene1", "Gene2", "Gene3"),
                         group_by = "cell_type")
  inherits(p, "ggplot")
}
# =============================================================================
# sc_module_score()  —  Gene-set module score per cell (Seurat)
#
# Method : Seurat::AddModuleScore averages the expression of a gene set per cell
#          and subtracts the average of a control set matched for expression level
#          (genes are binned into `nbin` expression bins and `ctrl` controls are
#          drawn per bin). A positive score means the set is expressed above its
#          matched background. Output columns are renamed to the gene-set names.
# Input  : seurat_obj   a Seurat object with normalized data; gene_sets   a named
#          list() of character vectors of gene symbols.
# Output : the Seurat object with one score column per set (named by set) in
#          meta.data.
# Depends: Seurat.
# =============================================================================

#' Gene-set module score per cell (Seurat)
#'
#' @param seurat_obj a Seurat object with normalized data.
#' @param gene_sets named \code{list()} of gene-symbol character vectors.
#' @param nbin number of expression bins for control matching (default 24).
#' @param ctrl number of control genes sampled per bin (default 100); reduce for
#'     very small feature spaces.
#' @param seed random seed for control sampling (default 1).
#' @return the Seurat object with module-score columns named by gene set.
sc_module_score <- function(seurat_obj, gene_sets, nbin = 24, ctrl = 100,
                            seed = 1) {
  if (is.null(names(gene_sets)) || any(names(gene_sets) == ""))
```

```r
    stop("`gene_sets` must be a fully named list.")
  seurat_obj <- Seurat::AddModuleScore(seurat_obj, features = gene_sets,
                                       name = "modulescore_", nbin = nbin,
                                       ctrl = ctrl, seed = seed)
  added <- grep("^modulescore_[0-9]+$", colnames(seurat_obj[[]]), value = TRUE)
  added <- added[order(as.integer(sub("modulescore_", "", added)))]
  colnames(seurat_obj@meta.data)[
    match(added, colnames(seurat_obj@meta.data))] <- names(gene_sets)
  seurat_obj
}

# ---- Example (simulated: build a tiny Seurat object) -----------------------
if (FALSE) {
  library(Seurat)
  set.seed(1)
  counts <- matrix(rpois(300 * 80, 4), nrow = 300,
                   dimnames = list(paste0("Gene", 1:300), paste0("Cell", 1:80)))
  so    <- CreateSeuratObject(counts)
  so    <- NormalizeData(so, verbose = FALSE)
  sets <- list(SetA = paste0("Gene", 1:12), SetB = paste0("Gene", 13:24))
  so    <- sc_module_score(so, sets, nbin = 8, ctrl = 5)   # small ctrl for few genes
  head(so[[]][, c("SetA", "SetB")])
}
# ============================================================================
# sc_normalize()  —  Log-normalize a single-cell counts assay
#
# Method : Thin wrapper over Seurat::NormalizeData. The default "LogNormalize"
#          divides each cell's counts by its total, multiplies by `scale_factor`
#          and log1p-transforms  —  the standard input for scoring, differential
#          expression and visualization. For UMI data with strong technical
#          variation, Seurat::SCTransform (variance-stabilizing regularized
#          negative-binomial residuals) is an alternative that replaces this step;
#          it is not wrapped here.
# Input   : seurat_obj   a Seurat object with a counts layer.
# Output : the same Seurat object with a normalized "data" layer populated.
# Depends: Seurat.
# ============================================================================

#' Log-normalize a single-cell counts assay
#'
#' @param seurat_obj a Seurat object.
#' @param method normalization method for \code{\link[Seurat]{NormalizeData}}
#'    (default "LogNormalize").
#' @param scale_factor per-cell scaling factor applied before log1p
#'    (default 1e4).
#' @param assay assay to normalize; \code{NULL} uses the default assay.
#' @return the Seurat object with normalized data.
sc_normalize <- function(seurat_obj, method = "LogNormalize",
                         scale_factor = 1e4, assay = NULL) {
  Seurat::NormalizeData(seurat_obj, normalization.method = method,
                        scale.factor = scale_factor, assay = assay,
                        verbose = FALSE)
}

# ---- Example (simulated: build a tiny Seurat object) -----------------------
if (FALSE) {
  library(Seurat)
```

```r
  set.seed(1)
  counts <- matrix(rpois(300 * 80, 4), nrow = 300,
                   dimnames = list(paste0("Gene", 1:300), paste0("Cell", 1:80)))
  so <- CreateSeuratObject(counts)
  so <- sc_normalize(so)
  range(GetAssayData(so, assay = "RNA", layer = "data"))    # normalized values
}
# ==============================================================================
# sc_proportion_bar()  —  Cell-type composition bar plot per sample
#
# Method : Cells are cross-tabulated by sample and cell type and drawn as a stacked
#          bar per sample with ggplot2. The default position "fill" shows each
#          sample's composition as proportions (bars sum to 1), the standard view
#          for comparing cell-type makeup across conditions; "stack" shows raw
#          counts instead.
# Input  : x  a Seurat object or a data.frame carrying the sample and cell-type
#          columns.
# Output : a ggplot object (the caller prints it or saves with ggplot2::ggsave).
# Depends: ggplot2 (a Seurat object is read through its meta.data slot).
# ==============================================================================

#' Cell-type composition bar plot per sample
#'
#' @param x a Seurat object, or a data.frame of per-cell metadata.
#' @param sample_col metadata column identifying the sample / group (x axis).
#' @param celltype_col metadata column identifying the cell type (fill).
#' @param position "fill" for proportions (default) or "stack" for counts.
#' @param palette optional colours passed to \code{scale_fill_manual}.
#' @return a \code{ggplot} object.
sc_proportion_bar <- function(x, sample_col = "sample",
                              celltype_col = "cell_type",
                              position = c("fill", "stack"), palette = NULL) {
  position <- match.arg(position)
  md <- if (inherits(x, "data.frame")) x else x@meta.data
  for (col in c(sample_col, celltype_col))
    if (!col %in% colnames(md))
      stop("column '", col, "' not found in the input metadata.")

  tab <- as.data.frame(table(sample   = md[[sample_col]],
                             celltype = md[[celltype_col]]))
  ylab <- if (position == "fill") "Proportion of cells" else "Number of cells"

  p <- ggplot2::ggplot(tab, ggplot2::aes(x = sample, y = Freq,
                                         fill = celltype)) +
    ggplot2::geom_col(position = position, width = 0.9) +
    ggplot2::labs(x = NULL, y = ylab, fill = celltype_col) +
    ggplot2::theme_classic(base_size = 12) +
    ggplot2::theme(axis.text.x = ggplot2::element_text(angle = 45, hjust = 1))
  if (!is.null(palette))
    p <- p + ggplot2::scale_fill_manual(values = palette)
  p
}

# ---- Example (simulated: build a tiny Seurat object) ------------------------
if (FALSE) {
  library(Seurat)
  set.seed(1)
```

```
  counts <- matrix(rpois(300 * 80, 4), nrow = 300,
                   dimnames = list(paste0("Gene", 1:300), paste0("Cell", 1:80)))
  so <- CreateSeuratObject(counts)
  so$sample    <- rep(c("S1", "S2"), times = 40)
  so$cell_type <- rep(c("T", "B", "Myeloid", "NK"), each = 20)
  p <- sc_proportion_bar(so, sample_col = "sample", celltype_col = "cell_type")
  inherits(p, "ggplot")
}
# ============================================================================
# sc_pseudobulk_correlation()  —  Two-gene correlation across pseudobulk profiles
#
# Method : Single cells are aggregated into pseudobulk profiles by summing raw
#          counts within each sample (optionally sample x cell type)  —  the standard
#          way to recover sample-level, biologically-replicated measurements from
#          single-cell data and avoid the inflated significance of treating each
#          cell as a replicate. Pseudobulk counts are CPM-normalized and log1p-
#          transformed, then the two genes are correlated across profiles with
#          cor.test (Pearson by default).
# Input  : seurat_obj   a Seurat object; gene1, gene2   gene symbols; sample_col   a
#          meta.data column identifying the biological sample.
# Output : list(correlation = htest, pseudobulk = log-CPM matrix (genes x
#          profiles), data = per-profile values for the two genes).
# Depends: SeuratObject, base R.
# ============================================================================

#' Two-gene correlation across pseudobulk profiles
#'
#' @param seurat_obj a Seurat object.
#' @param gene1,gene2 gene symbols to correlate.
#' @param sample_col meta.data column identifying the biological sample.
#' @param celltype_col optional meta.data column; when given, pseudobulk is formed
#'   per sample x cell type.
#' @param assay assay to aggregate (default "RNA").
#' @param method correlation method for \code{\link[stats]{cor.test}}
#'   (default "pearson").
#' @return a list with the correlation test, the log-CPM pseudobulk matrix and the
#'   two-gene per-profile values.
sc_pseudobulk_correlation <- function(seurat_obj, gene1, gene2, sample_col,
                                      celltype_col = NULL, assay = "RNA",
                                      method = "pearson") {
  md <- seurat_obj[[]]
  if (!sample_col %in% colnames(md))
    stop("`sample_col` '", sample_col, "' not found in meta.data.")
  counts <- as.matrix(SeuratObject::GetAssayData(seurat_obj, assay = assay,
                                                 layer = "counts"))
  absent <- setdiff(c(gene1, gene2), rownames(counts))
  if (length(absent))
    stop("gene(s) not found: ", paste(absent, collapse = ", "))

  grp <- as.character(md[[sample_col]])
  if (!is.null(celltype_col)) {
    if (!celltype_col %in% colnames(md))
      stop("`celltype_col` '", celltype_col, "' not found in meta.data.")
    grp <- paste(grp, as.character(md[[celltype_col]]), sep = "__")
  }
  pb <- t(rowsum(t(counts), group = grp))            # genes x profiles (summed)
  pb <- pb[, colSums(pb) > 0, drop = FALSE]
```

```r
  logcpm <- log1p(sweep(pb, 2, colSums(pb), "/") * 1e6)

  d <- data.frame(profile = colnames(logcpm),
                  g1 = logcpm[gene1, ], g2 = logcpm[gene2, ], row.names = NULL)
  names(d)[2:3] <- c(gene1, gene2)
  ct <- stats::cor.test(logcpm[gene1, ], logcpm[gene2, ], method = method)
  list(correlation = ct, pseudobulk = logcpm, data = d)
}

# ---- Example (simulated: build a tiny Seurat object) -----------------------
if (FALSE) {
  library(Seurat)
  set.seed(1)
  counts <- matrix(rpois(300 * 80, 4), nrow = 300,
                   dimnames = list(paste0("Gene", 1:300), paste0("Cell", 1:80)))
  counts["Gene2", ] <- counts["Gene1", ] + rpois(80, 1)   # couple Gene1 & Gene2
  so <- CreateSeuratObject(counts)
  so$sample     <- rep(c("S1", "S2"), times = 40)
  so$cell_type <- rep(c("T", "B", "Myeloid", "NK"), each = 20)
  res <- sc_pseudobulk_correlation(so, "Gene1", "Gene2", sample_col = "sample",
                                   celltype_col = "cell_type")
  res$correlation
}
# ============================================================================
# sc_ridge()  —  Ridge plots of feature expression by group
#
# Method : Seurat::RidgePlot draws, per feature, overlapping density ridgelines of
#          expression for each group (a meta.data column or the active
#          identities)  —  a compact alternative to violins for comparing where each
#          group's expression mass sits. A thin wrapper returning the plot.
# Input  : seurat_obj   a Seurat object; features   a character vector of genes or
#          meta.data columns; group_by   optional grouping column.
# Output : a ggplot object (single feature) or a patchwork of panels (several).
# Depends: Seurat.
# ============================================================================

#' Ridge plots of feature expression by group
#'
#' @param seurat_obj a Seurat object.
#' @param features character vector of genes or meta.data columns.
#' @param group_by meta.data column to group by; \code{NULL} uses active
#'    identities.
#' @param ... further arguments passed to \code{\link[Seurat]{RidgePlot}}.
#' @return a \code{ggplot} / \code{patchwork} object.
sc_ridge <- function(seurat_obj, features, group_by = NULL, ...) {
  Seurat::RidgePlot(seurat_obj, features = features, group.by = group_by, ...)
}

# ---- Example (simulated: build a tiny Seurat object) -----------------------
if (FALSE) {
  library(Seurat)
  set.seed(1)
  counts <- matrix(rpois(300 * 80, 4), nrow = 300,
                   dimnames = list(paste0("Gene", 1:300), paste0("Cell", 1:80)))
  so <- CreateSeuratObject(counts)
  so <- NormalizeData(so, verbose = FALSE)
  so$cell_type <- rep(c("T", "B", "Myeloid", "NK"), each = 20)
```

```
  p <- sc_ridge(so, features = "Gene1", group_by = "cell_type")
  inherits(p, "ggplot") || inherits(p, "patchwork")
}
# ==============================================================================
# sc_stacked_violin()  —  Stacked violin panel of markers across groups
#
# Method : A compact marker view built directly with ggplot2 (no extra Seurat
#          dependency for the layout): expression is pulled per cell for the
#          requested features and grouping, reshaped to long form, and drawn as
#          width-scaled violins faceted one row per gene with the inter-facet
#          spacing removed, so many markers stack into a single column. Gene order
#          follows the input.
# Input  : seurat_obj  a Seurat object with normalized data; features  a character
#          vector of genes; group_by  optional grouping column.
# Output : a ggplot object (the caller prints it or saves with ggplot2::ggsave).
# Depends: SeuratObject (data extraction), ggplot2.
# ==============================================================================

#' Stacked violin panel of markers across groups
#'
#' @param seurat_obj a Seurat object with normalized data.
#' @param features character vector of genes, one violin row each (input order
#'   preserved).
#' @param group_by meta.data column to group by; \code{NULL} uses active
#'   identities.
#' @param assay assay to read (default "RNA").
#' @param layer assay layer to read (default "data").
#' @param palette optional colours passed to \code{scale_fill_manual}.
#' @return a \code{ggplot} object.
sc_stacked_violin <- function(seurat_obj, features, group_by = NULL,
                              assay = "RNA", layer = "data", palette = NULL) {
  features <- unique(features)
  expr <- SeuratObject::FetchData(seurat_obj, vars = features, layer = layer)
  expr <- as.matrix(expr[, features, drop = FALSE])      # keep requested order
  group <- if (is.null(group_by)) SeuratObject::Idents(seurat_obj)
           else seurat_obj[[group_by, drop = TRUE]]

  long <- data.frame(
    group   = factor(rep(group, times = ncol(expr))),
    feature = factor(rep(features, each = nrow(expr)), levels = features),
    expr    = as.vector(expr))

  p <- ggplot2::ggplot(long, ggplot2::aes(x = group, y = expr, fill = group)) +
    ggplot2::geom_violin(scale = "width", trim = TRUE, linewidth = 0.2) +
    ggplot2::facet_grid(feature ~ ., scales = "free_y", switch = "y") +
    ggplot2::labs(x = NULL, y = NULL) +
    ggplot2::theme_classic(base_size = 11) +
    ggplot2::theme(
      legend.position    = "none",
      panel.spacing      = ggplot2::unit(0, "lines"),
      strip.background   = ggplot2::element_blank(),
      strip.text.y.left = ggplot2::element_text(angle = 0, hjust = 1),
      strip.placement    = "outside",
      axis.text.x        = ggplot2::element_text(angle = 45, hjust = 1),
      axis.text.y        = ggplot2::element_blank(),
      axis.ticks.y       = ggplot2::element_blank())
  if (!is.null(palette))
```

```r
    p <- p + ggplot2::scale_fill_manual(values = palette)
  p
}

# ---- Example (simulated: build a tiny Seurat object) -----------------------
if (FALSE) {
  library(Seurat)
  set.seed(1)
  counts <- matrix(rpois(300 * 80, 4), nrow = 300,
                   dimnames = list(paste0("Gene", 1:300), paste0("Cell", 1:80)))
  so <- CreateSeuratObject(counts)
  so <- NormalizeData(so, verbose = FALSE)
  so$cell_type <- rep(c("T", "B", "Myeloid", "NK"), each = 20)
  p <- sc_stacked_violin(so, features = c("Gene1", "Gene2", "Gene3"),
                         group_by = "cell_type")
  inherits(p, "ggplot")
}
# ============================================================================
# sc_umap_by_cluster()  —  UMAP scatter coloured by a cell annotation
#
# Method : Pull a 2-D embedding (default "umap") and a metadata label column
#          from a Seurat object and draw the scatter directly with ggplot2  —
#          one colour per label, with centred text labels. A custom renderer,
#          it does not call Seurat::DimPlot, so colours / labels / theme are
#          fully under your control.
# Input  : seurat_obj   a Seurat object with the reduction computed and
#          `label_col` present in meta.data.
# Output : a ggplot object (the caller prints it or saves with ggplot2::ggsave).
# Depends: SeuratObject (or Seurat), ggplot2.
# ============================================================================

#' UMAP scatter coloured by a cell annotation
#'
#' @param seurat_obj a Seurat object.
#' @param label_col meta.data column to colour by (default "cell_type").
#' @param reduction name of the dimensionality reduction to plot (default "umap").
#' @param pt_size point size (default 0.4).
#' @param label logical; draw a text label at each group's median position.
#' @param palette optional named/character vector of colours passed to
#'   \code{scale_colour_manual}.
#' @return a \code{ggplot} object.
sc_umap_by_cluster <- function(seurat_obj, label_col = "cell_type",
                               reduction = "umap", pt_size = 0.4,
                               label = TRUE, palette = NULL) {
  emb <- SeuratObject::Embeddings(seurat_obj, reduction = reduction)[, 1:2]
  md  <- seurat_obj[[]]
  if (!label_col %in% colnames(md))
    stop("`label_col` '", label_col, "' not found in meta.data.")

  d <- data.frame(dim1 = emb[, 1], dim2 = emb[, 2],
                  label = as.factor(md[[label_col]]))
  axis <- toupper(reduction)

  p <- ggplot2::ggplot(d, ggplot2::aes(x = dim1, y = dim2, colour = label)) +
    ggplot2::geom_point(size = pt_size, stroke = 0) +
    ggplot2::labs(x = paste0(axis, "_1"), y = paste0(axis, "_2"),
                  colour = label_col) +
```

```
    ggplot2::guides(colour = ggplot2::guide_legend(
      override.aes = list(size = 3))) +
    ggplot2::theme_classic(base_size = 12) +
    ggplot2::theme(aspect.ratio = 1)

  if (!is.null(palette))
    p <- p + ggplot2::scale_colour_manual(values = palette)

  if (isTRUE(label)) {
    cen <- stats::aggregate(cbind(dim1, dim2) ~ label, data = d, FUN = stats::median)
    p <- p + ggplot2::geom_text(
      data = cen, ggplot2::aes(x = dim1, y = dim2, label = label),
      colour = "black", size = 4, fontface = "bold", show.legend = FALSE)
  }
  p
}

# ---- Example (simulated: build a tiny Seurat object) ------------------------
if (FALSE) {
  library(Seurat)
  set.seed(1)
  counts <- matrix(rpois(200 * 60, 5), nrow = 200,
                   dimnames = list(paste0("Gene", 1:200), paste0("Cell", 1:60)))
  so   <- CreateSeuratObject(counts)
  emb <- rbind(matrix(rnorm(40, -5), 20), matrix(rnorm(40, 0), 20),
               matrix(rnorm(40,  5), 20))
  colnames(emb) <- c("UMAP_1", "UMAP_2"); rownames(emb) <- colnames(so)
  so[["umap"]] <- CreateDimReducObject(embeddings = emb, key = "UMAP_", assay = "RNA")
  so$cell_type <- rep(c("T", "B", "Myeloid"), each = 20)
  print(sc_umap_by_cluster(so, "cell_type"))
}
# =============================================================================
# sc_violin()  —  Violin plots of feature expression by group
#
# Method : Seurat::VlnPlot draws, per feature, the distribution of expression
#          across cells split by a grouping (a meta.data column or the active
#          identities). Point overlay is off by default (pt_size = 0) for clean
#          distributions on large objects. A thin wrapper returning the plot.
# Input  : seurat_obj  a Seurat object; features  a character vector of genes or
#          meta.data columns; group_by  optional grouping column.
# Output : a ggplot object (single feature) or a patchwork of panels (several).
# Depends: Seurat.
# =============================================================================

#' Violin plots of feature expression by group
#'
#' @param seurat_obj a Seurat object.
#' @param features character vector of genes or meta.data columns.
#' @param group_by meta.data column to group by; \code{NULL} uses active
#'   identities.
#' @param pt_size jitter point size; 0 hides points (default 0).
#' @param ... further arguments passed to \code{\link[Seurat]{VlnPlot}}.
#' @return a \code{ggplot} / \code{patchwork} object.
sc_violin <- function(seurat_obj, features, group_by = NULL, pt_size = 0, ...) {
  Seurat::VlnPlot(seurat_obj, features = features, group.by = group_by,
                  pt.size = pt_size, ...)
}
```

```r
# ---- Example (simulated: build a tiny Seurat object) ------------------------
if (FALSE) {
  library(Seurat)
  set.seed(1)
  counts <- matrix(rpois(300 * 80, 4), nrow = 300,
                   dimnames = list(paste0("Gene", 1:300), paste0("Cell", 1:80)))
  so <- CreateSeuratObject(counts)
  so <- NormalizeData(so, verbose = FALSE)
  so$cell_type <- rep(c("T", "B", "Myeloid", "NK"), each = 20)
  p <- sc_violin(so, features = c("Gene1", "Gene2"), group_by = "cell_type")
  inherits(p, "ggplot") || inherits(p, "patchwork")
}
# ==============================================================================
# spatial_celltype_proportion_plot() — Spatial map of one cell type's proportion
#
# Method : Draw spots at their tissue coordinates and colour them by one chosen
#          cell type's per-spot proportion from a deconvolution result
#          (spots x cell types), aligning the proportion matrix to the coordinate
#          table by spot ID. Rendered with ggplot2 — geom_point over
#          coord_fixed(ratio = 1), a viridis colour scale, and a stripped-down
#          theme (the same layout as the other spatial maps in this folder).
# Input  : coords data.frame with columns x, y and a spot ID (a `spot` column or
#                 the rownames) — one row per spot.
#          props    spots x cell-types numeric matrix / data.frame; rownames =
#                 spot IDs, one column per cell type.
#          cell_type name of the cell type (a column of `props`) to map.
# Output : a ggplot object (the caller prints it or saves with ggplot2::ggsave).
# Depends: ggplot2.
# ==============================================================================

#' Spatial map of one cell type's deconvolution proportion
#'
#' @param coords data.frame with columns \code{x}, \code{y} and spot IDs, given
#'   either as a \code{spot} column or as the data.frame rownames.
#' @param props spots x cell-types numeric matrix or data.frame; rownames are
#'   spot IDs, columns are cell types.
#' @param cell_type name of the cell type to map; must be a column of
#'   \code{props}.
#' @param pt_size point size (default 1.6).
#' @param flip_y reverse the y axis so the map matches image coordinates, whose
#'   origin is at the top-left (default FALSE).
#' @param option viridis colour-map option passed to
#'   \code{\link[ggplot2]{scale_colour_viridis_c}} (default "viridis").
#' @return a \code{ggplot} object.
spatial_celltype_proportion_plot <- function(coords, props, cell_type,
                                             pt_size = 1.6, flip_y = FALSE,
                                             option = "viridis") {
  coords <- as.data.frame(coords)
  if (!all(c("x", "y") %in% colnames(coords)))
    stop("`coords` must have columns x and y.")
  ids <- if ("spot" %in% colnames(coords)) as.character(coords$spot)
         else rownames(coords)
  if (is.null(ids))
    stop("`coords` needs spot IDs in a `spot` column or as rownames.")

  props <- as.matrix(props)
```

```r
  if (is.null(rownames(props)))
    stop("`props` must have spot IDs as rownames.")
  if (!cell_type %in% colnames(props))
    stop("`cell_type` (", cell_type, ") is not a column of `props`.")

  common <- intersect(ids, rownames(props))
  if (!length(common))
    stop("no shared spot IDs between `coords` and `props`.")
  keep <- ids %in% common
  d <- data.frame(x = as.numeric(coords$x[keep]),
                  y = as.numeric(coords$y[keep]),
                  value = as.numeric(props[ids[keep], cell_type]))

  p <- ggplot2::ggplot(d, ggplot2::aes(x = x, y = y, colour = value)) +
    ggplot2::geom_point(size = pt_size, stroke = 0) +
    ggplot2::scale_colour_viridis_c(option = option) +
    ggplot2::coord_fixed(ratio = 1) +
    ggplot2::labs(colour = cell_type) +
    ggplot2::theme_minimal(base_size = 12) +
    ggplot2::theme(panel.grid   = ggplot2::element_blank(),
                   axis.text    = ggplot2::element_blank(),
                   axis.ticks   = ggplot2::element_blank(),
                   axis.title   = ggplot2::element_blank())
  if (isTRUE(flip_y)) p <- p + ggplot2::scale_y_reverse()
  p
}

# ---- Example (simulated data; select this block and run) --------------------
if (FALSE) {
  library(ggplot2)
  set.seed(1)
  n <- 400
  coords <- data.frame(spot = paste0("Spot", 1:n),
                       x = runif(n, 0, 30), y = runif(n, 0, 30))
  props <- matrix(runif(n * 4), nrow = n,
                  dimnames = list(paste0("Spot", 1:n),
                                  c("Tcell", "Bcell", "Myeloid", "Fibroblast")))
  props <- props / rowSums(props)                              # per-spot proportions
  props[coords$x < 15, "Tcell"] <- props[coords$x < 15, "Tcell"] + 0.5   # enrich left half
  p <- spatial_celltype_proportion_plot(coords, props, cell_type = "Tcell")
  inherits(p, "ggplot")
}
# ==============================================================================
# spatial_cluster_plot()  —  Spatial map coloured by a discrete cluster / region
#
# Method : Draw spots at their tissue coordinates and colour them by a discrete
#          label (a spatial cluster or histological region) with ggplot2  —
#          geom_point over coord_fixed(ratio = 1), a discrete colour scale, and a
#          stripped-down theme. A custom renderer; it does not call
#          Seurat::SpatialDimPlot, so colours / theme are fully under your
#          control.
# Input  : data data.frame with columns x, y, cluster (one row per spot);
#               cluster is coerced to a factor.
# Output : a ggplot object (the caller prints it or saves with ggplot2::ggsave).
# Depends: ggplot2.
# ==============================================================================
```

```r
#' Spatial map coloured by a discrete cluster or region
#'
#' @param data data.frame with columns \code{x}, \code{y}, \code{cluster} (one
#'     row per spot).
#' @param pt_size point size (default 1.6).
#' @param flip_y reverse the y axis so the map matches image coordinates, whose
#'     origin is at the top-left (default FALSE).
#' @param palette optional character vector of colours passed to
#'     \code{\link[ggplot2]{scale_colour_manual}}.
#' @param legend_title legend title (default "cluster").
#' @return a \code{ggplot} object.
spatial_cluster_plot <- function(data, pt_size = 1.6, flip_y = FALSE,
                                 palette = NULL, legend_title = "cluster") {
  d <- as.data.frame(data)
  if (!all(c("x", "y", "cluster") %in% colnames(d)))
    stop("`data` must have columns x, y, cluster.")
  d <- data.frame(x = as.numeric(d$x), y = as.numeric(d$y),
                  cluster = as.factor(d$cluster))

  p <- ggplot2::ggplot(d, ggplot2::aes(x = x, y = y, colour = cluster)) +
    ggplot2::geom_point(size = pt_size, stroke = 0) +
    ggplot2::coord_fixed(ratio = 1) +
    ggplot2::labs(colour = legend_title) +
    ggplot2::guides(colour = ggplot2::guide_legend(
      override.aes = list(size = 3))) +
    ggplot2::theme_minimal(base_size = 12) +
    ggplot2::theme(panel.grid   = ggplot2::element_blank(),
                   axis.text    = ggplot2::element_blank(),
                   axis.ticks   = ggplot2::element_blank(),
                   axis.title   = ggplot2::element_blank())
  if (!is.null(palette))
    p <- p + ggplot2::scale_colour_manual(values = palette)
  if (isTRUE(flip_y)) p <- p + ggplot2::scale_y_reverse()
  p
}

# ---- Example (simulated data; select this block and run) --------------------
if (FALSE) {
  library(ggplot2)
  set.seed(1)
  n <- 500
  d <- data.frame(x = runif(n, 0, 30), y = runif(n, 0, 30))
  d$cluster <- ifelse((d$x - 15)^2 + (d$y - 15)^2 < 60, "core",
                      ifelse(d$x < 15, "left", "right"))      # 3 spatial domains
  p <- spatial_cluster_plot(d)
  inherits(p, "ggplot")
}
# ==============================================================================
# spatial_feature_plot()  —  Spatial map of a continuous value over tissue
#
# Method : Draw spots at their tissue coordinates and colour them by a continuous
#          value (e.g. a gene's expression) with ggplot2  —  geom_point over
#          coord_fixed(ratio = 1) so distances are undistorted, a viridis colour
#          scale, and a stripped-down theme. A custom renderer; it does not call
#          Seurat::SpatialFeaturePlot, so colours / theme are fully under your
#          control. Accepts a plain data.frame of coordinates + value, or a
#          Seurat spatial object plus a gene name (coordinates and expression are
```

```r
#                   pulled from it).
# Input    : data    data.frame with columns x, y, value (one row per spot); OR a
#                             Seurat object, in which case `gene` selects the value.
#                   gene    gene symbol to colour by when `data` is a Seurat object.
# Output : a ggplot object (the caller prints it or saves with ggplot2::ggsave).
# Depends: ggplot2; Seurat only for the optional object input (guarded).
# ==============================================================================

#' Spatial map of a continuous value over tissue coordinates
#'
#' @param data a data.frame with columns \code{x}, \code{y}, \code{value} (one
#'     row per spot), or a Seurat spatial object.
#' @param gene gene symbol used as the value when \code{data} is a Seurat object;
#'     ignored for the data.frame input.
#' @param pt_size point size (default 1.6).
#' @param flip_y reverse the y axis so the map matches image coordinates, whose
#'     origin is at the top-left (default FALSE).
#' @param option viridis colour-map option passed to
#'     \code{\link[ggplot2]{scale_colour_viridis_c}} (default "viridis").
#' @param legend_title legend title (default "value").
#' @return a \code{ggplot} object.
spatial_feature_plot <- function(data, gene = NULL, pt_size = 1.6,
                                 flip_y = FALSE, option = "viridis",
                                 legend_title = "value") {
  if (inherits(data, "Seurat")) {
    if (is.null(gene))
      stop("`gene` is required when `data` is a Seurat object.")
    if (!requireNamespace("Seurat", quietly = TRUE))
      stop("package 'Seurat' is needed for the Seurat-object input.")
    co <- as.data.frame(Seurat::GetTissueCoordinates(data))
    cn <- colnames(co)
    if (all(c("imagecol", "imagerow") %in% cn)) {           # image (pixel) coordinates
      xx <- co$imagecol; yy <- co$imagerow
    } else if (all(c("x", "y") %in% cn)) {
      xx <- co$x; yy <- co$y
    } else {
      num <- co[, vapply(co, is.numeric, logical(1)), drop = FALSE]
      if (ncol(num) < 2) stop("could not find two coordinate columns.")
      xx <- num[[1]]; yy <- num[[2]]
    }
    val <- as.numeric(Seurat::FetchData(data, vars = gene)[, 1])
    d <- data.frame(x = as.numeric(xx), y = as.numeric(yy), value = val)
  } else {
    d <- as.data.frame(data)
    if (!all(c("x", "y", "value") %in% colnames(d)))
      stop("`data` must have columns x, y, value.")
    d <- data.frame(x = as.numeric(d$x), y = as.numeric(d$y),
                    value = as.numeric(d$value))
  }

  p <- ggplot2::ggplot(d, ggplot2::aes(x = x, y = y, colour = value)) +
    ggplot2::geom_point(size = pt_size, stroke = 0) +
    ggplot2::scale_colour_viridis_c(option = option) +
    ggplot2::coord_fixed(ratio = 1) +
    ggplot2::labs(colour = legend_title) +
    ggplot2::theme_minimal(base_size = 12) +
    ggplot2::theme(panel.grid    = ggplot2::element_blank(),
```

```r
                          axis.text      = ggplot2::element_blank(),
                          axis.ticks     = ggplot2::element_blank(),
                          axis.title     = ggplot2::element_blank())
  if (isTRUE(flip_y)) p <- p + ggplot2::scale_y_reverse()
  p
}

# ---- Example (simulated data; select this block and run) --------------------
if (FALSE) {
  library(ggplot2)
  set.seed(1)
  n <- 500
  d <- data.frame(x = runif(n, 0, 30), y = runif(n, 0, 30))
  d$value <- exp(-((d$x - 15)^2 + (d$y - 15)^2) / 50) + rnorm(n, sd = 0.05)    # central hotspot
  p <- spatial_feature_plot(d, flip_y = TRUE)
  inherits(p, "ggplot")
}
# ==============================================================================
# spatial_gene_celltype_correlation()  —  Gene expression vs cell-type proportions
#
# Method : Correlate one gene's per-spot expression against each cell type's
#          per-spot proportion from a deconvolution result (spots x cell types).
#          Spots are aligned by name (names of the gene vector vs rownames of the
#          proportion matrix); one stats::cor.test per cell type (Spearman by
#          default) on shared spots where both values are finite; Benjamini-
#          Hochberg across cell types.
# Input  : gene     named numeric vector; names = spot IDs, values = expression.
#          props    spots x cell-types numeric matrix / data.frame of deconvolution
#                   proportions; rownames = spot IDs, one column per cell type.
#          method "spearman" (default) or "pearson".
# Output : data.frame(cell_type, rho, p.value, p.adj, n) ordered by p.value.
#          Compute only  —  no plotting.
# Depends: base R only.
# ==============================================================================

#' Correlate a gene's spot expression against cell-type deconvolution proportions
#'
#' @param gene named numeric vector of per-spot expression; names are spot IDs.
#' @param props spots x cell-types numeric matrix or data.frame of deconvolution
#'   proportions; rownames are spot IDs, columns are cell types.
#' @param method correlation method, "spearman" (default) or "pearson".
#' @param p_adjust multiple-testing method for \code{\link[stats]{p.adjust}}
#'   (default "BH").
#' @param min_pairs minimum complete (both-finite) shared spots per cell type
#'   (default 3).
#' @return data.frame, one row per cell type, ordered by \code{p.value}. The
#'   \code{rho} column holds the correlation coefficient for the chosen method.
spatial_gene_celltype_correlation <- function(gene, props,
                                              method = c("spearman", "pearson"),
                                              p_adjust = "BH", min_pairs = 3) {
  method <- match.arg(method)
  props    <- as.matrix(props)
  if (is.null(names(gene)) || is.null(rownames(props)))
    stop("`gene` must be named by spot and `props` must have rownames.")
  common <- intersect(names(gene), rownames(props))
  if (length(common) < min_pairs)
    stop("only ", length(common), " shared spots; need >= ", min_pairs, ".")
```

```r
  g        <- as.numeric(gene[common])
  props <- props[common, , drop = FALSE]
  cts     <- colnames(props)
  if (is.null(cts)) cts <- paste0("celltype", seq_len(ncol(props)))

  stat <- t(vapply(seq_len(ncol(props)), function(j) {
    y   <- as.numeric(props[, j])
    ok <- is.finite(g) & is.finite(y)
    n   <- sum(ok)
    if (n < min_pairs) return(c(rho = NA_real_, p = NA_real_, n = n))
    ht <- suppressWarnings(stats::cor.test(g[ok], y[ok], method = method))
    c(rho = unname(ht$estimate), p = ht$p.value, n = n)
  }, numeric(3)))

  out <- data.frame(cell_type = cts, rho = stat[, "rho"],
                    p.value = stat[, "p"],
                    p.adj = stats::p.adjust(stat[, "p"], method = p_adjust),
                    n = stat[, "n"], row.names = NULL)
  out <- out[order(out$p.value), ]
  rownames(out) <- NULL
  out
}

# ---- Example (simulated data; select this block and run) -------------------
if (FALSE) {
  set.seed(1)
  spots <- paste0("Spot", 1:80)
  gene   <- setNames(rnorm(80), spots)
  props <- matrix(runif(80 * 5), nrow = 80,
                  dimnames = list(spots, paste0("CellType", 1:5)))
  props <- props / rowSums(props)                          # proportions sum to 1
  props[, 1] <- (gene - min(gene)) / diff(range(gene))     # CellType1 tracks the gene
  res <- spatial_gene_celltype_correlation(gene, props, method = "spearman")
  res
}
# ============================================================================
# spatial_gene_gene_correlation()  —  Spot-level correlation between two genes
#
# Method : Pearson or Spearman correlation between the per-spot expression of two
#          genes pulled from a spatial expression matrix, via a single
#          stats::cor.test. Only spots where both genes are finite are used
#          (equivalent to use = "pairwise.complete.obs").
# Input  : expr      numeric matrix, genes in rows (rownames = gene symbols), spots
#                    in columns.
#          gene1, gene2    two gene symbols present in rownames(expr).
#          method "pearson" (default) or "spearman".
# Output : list(gene1, gene2, method, estimate, conf.int, statistic, p.value, n).
#          conf.int is NULL for Spearman. Compute only  —  no plotting.
# Depends: base R only.
# ============================================================================

#' Spot-level correlation between two genes
#'
#' @param expr numeric matrix (genes x spots); rownames are gene symbols, spots
#'    in columns.
#' @param gene1,gene2 gene symbols to correlate; both must be in
#'    \code{rownames(expr)}.
```

```r
#' @param method correlation method, "pearson" (default) or "spearman".
#' @param min_pairs minimum number of complete (both-finite) spots required
#'     (default 3).
#' @return a list with the two gene names, method, correlation estimate,
#'     confidence interval (Pearson only; NULL otherwise), test statistic, p-value,
#'     and the number of complete spots used.
spatial_gene_gene_correlation <- function(expr, gene1, gene2,
                                          method = c("pearson", "spearman"),
                                          min_pairs = 3) {
  method <- match.arg(method)
  expr <- as.matrix(expr)
  for (g in c(gene1, gene2))
    if (!g %in% rownames(expr))
      stop("gene '", g, "' is not in rownames(expr).")

  x <- as.numeric(expr[gene1, ]); y <- as.numeric(expr[gene2, ])
  ok <- is.finite(x) & is.finite(y)
  n <- sum(ok)
  if (n < min_pairs)
    stop("need at least ", min_pairs, " complete spots; got ", n, ".")

  ht <- suppressWarnings(stats::cor.test(x[ok], y[ok], method = method))
  list(gene1     = gene1,
       gene2     = gene2,
       method    = method,
       estimate  = unname(ht$estimate),
       conf.int  = if (is.null(ht$conf.int)) NULL else unname(ht$conf.int),
       statistic = unname(ht$statistic),
       p.value   = ht$p.value,
       n         = n)
}

# ---- Example (simulated data; select this block and run) --------------------
if (FALSE) {
  set.seed(1)
  expr <- matrix(rnorm(50 * 120), nrow = 50,
                 dimnames = list(paste0("Gene", 1:50), paste0("Spot", 1:120)))
  expr["Gene2", ] <- 0.7 * expr["Gene1", ] + rnorm(120, sd = 0.5)   # Gene2 tracks Gene1
  spatial_gene_gene_correlation(expr, "Gene1", "Gene2", method = "pearson")
  spatial_gene_gene_correlation(expr, "Gene1", "Gene2", method = "spearman")
}
# ==============================================================================
# spatial_geneset_score()  —  Per-spot gene-set scores by mean z-score
#
# Method : Score each spot for each gene set as the mean, across the set's genes,
#          of the gene's z-score taken across spots (each gene is centred and
#          scaled to mean 0 / sd 1 over all spots, then averaged within the set).
#          This is a dependency-light stand-in for single-sample enrichment; the
#          packaged analyses use GSVA (GSVA::gsvaParam / GSVA::ssgseaParam) for
#          the same per-spot scoring when that package is available.
# Input  : expr      numeric matrix, genes in rows (rownames = gene symbols),
#                    spots in columns; values on a log scale.
#          genesets named list of character vectors of gene symbols.
# Output : numeric matrix, gene sets in rows, spots in columns (the per-spot
#          score for each set). Compute only  —  no plotting.
# Depends: base R only.
# ==============================================================================
```

```r
#' Per-spot gene-set scores by mean z-score
#'
#' @param expr numeric matrix (genes x spots); rownames are gene symbols, values
#'     on a log scale.
#' @param genesets named list; each element a character vector of gene symbols.
#' @param min_genes minimum number of set genes that must be present in
#'     \code{expr} to score a set; sets below this are returned as \code{NA}
#'     (default 1).
#' @return numeric matrix (gene sets x spots) of per-spot scores.
spatial_geneset_score <- function(expr, genesets, min_genes = 1) {
  expr <- as.matrix(expr)
  if (is.null(names(genesets)) || !is.list(genesets))
    stop("`genesets` must be a named list of gene-symbol vectors.")
  if (is.null(rownames(expr)))
    stop("`expr` must have gene symbols as rownames.")

  # z-score each gene across spots; genes with zero / non-finite sd -> 0
  mu    <- rowMeans(expr, na.rm = TRUE)
  sdev <- apply(expr, 1L, stats::sd, na.rm = TRUE)
  sdev[!is.finite(sdev) | sdev == 0] <- NA_real_
  z <- (expr - mu) / sdev
  z[!is.finite(z)] <- 0

  score <- t(vapply(genesets, function(gs) {
    present <- intersect(gs, rownames(z))
    if (length(present) < min_genes) return(rep(NA_real_, ncol(z)))
    colMeans(z[present, , drop = FALSE])
  }, numeric(ncol(z))))
  colnames(score) <- colnames(expr)
  score
}

# ---- Example (simulated data; select this block and run) --------------------
if (FALSE) {
  set.seed(1)
  expr <- matrix(rnorm(300 * 60), nrow = 300,
                 dimnames = list(paste0("Gene", 1:300), paste0("Spot", 1:60)))
  genesets <- list(setA = paste0("Gene", 1:20),
                   setB = paste0("Gene", 100:130))
  expr[genesets$setA, 1:20] <- expr[genesets$setA, 1:20] + 2   # setA high in first 20 spots
  sc <- spatial_geneset_score(expr, genesets)
  sc[, 1:5]
}
# =============================================================================
# spatial_region_de()  —  Differential expression between spatial regions
#
# Method : Per-gene two-group Wilcoxon rank-sum (Mann-Whitney U) test comparing
#          one spatial region against another, or against all remaining spots
#          pooled (e.g. a tumour-core zone vs the rest of a 3-zone boundary),
#          with Benjamini-Hochberg FDR control. The log2 fold change is the
#          difference of region means (expression is assumed on a log scale, so a
#          mean difference equals a log2 fold change).
# Input  : expr     numeric matrix, genes in rows (rownames = gene symbols),
#                   spots in columns; values on a log scale, e.g. log2(CPM + 1).
#          region   vector of length ncol(expr) giving each spot's region.
#          region1 the region tested (fold-change numerator).
```

```
#                   region2 the reference region; NULL pools all other spots as "rest".
# Output : data.frame(gene, mean_<region1>, mean_<region2>, log2FC, W, p.value,
#                   p.adj), ordered by p.value. Compute only  —  no plotting.
# Depends: base R only.
# ============================================================================

#' Differential expression between two spatial regions
#'
#' @param expr numeric matrix (genes x spots); rownames are gene symbols, values
#'      on a log scale.
#' @param region vector of length \code{ncol(expr)} giving each spot's region
#'      (e.g. a histological zone or a clustering-derived spatial domain).
#' @param region1 the region to test; its spots form the fold-change numerator.
#' @param region2 the reference region. If \code{NULL} (default) every spot not
#'      in \code{region1} is pooled as the reference ("rest").
#' @param p_adjust multiple-testing method for \code{\link[stats]{p.adjust}}
#'      (default "BH").
#' @param min_spots minimum spots required on each side to test a gene
#'      (default 3).
#' @return data.frame, one row per testable gene, ordered by \code{p.value}.
spatial_region_de <- function(expr, region, region1, region2 = NULL,
                              p_adjust = "BH", min_spots = 3) {
  expr <- as.matrix(expr)
  if (length(region) != ncol(expr))
    stop("length(region) must equal ncol(expr).")
  region <- as.character(region)
  if (!region1 %in% region)
    stop("`region1` (", region1, ") is not present in `region`.")

  i_case <- which(region == region1)
  if (is.null(region2)) {
    i_ref    <- which(region != region1)
    ref_name <- "rest"
  } else {
    if (!region2 %in% region)
      stop("`region2` (", region2, ") is not present in `region`.")
    i_ref    <- which(region == region2)
    ref_name <- region2
  }
  if (length(i_case) < min_spots || length(i_ref) < min_spots)
    stop("each side needs >= ", min_spots, " spots; got ",
         length(i_case), " and ", length(i_ref), ".")

  stat <- t(vapply(seq_len(nrow(expr)), function(g) {
    x <- expr[g, i_case]; y <- expr[g, i_ref]
    x <- x[is.finite(x)]; y <- y[is.finite(y)]
    if (length(x) < min_spots || length(y) < min_spots)
      return(c(m1 = NA_real_, m2 = NA_real_, lfc = NA_real_,
               W = NA_real_, p = NA_real_))
    ht <- suppressWarnings(stats::wilcox.test(x, y))
    c(m1 = mean(x), m2 = mean(y), lfc = mean(x) - mean(y),
      W = unname(ht$statistic), p = ht$p.value)
  }, numeric(5)))

  out <- data.frame(gene = rownames(expr), stat,
                    p.adj = stats::p.adjust(stat[, "p"], method = p_adjust),
                    row.names = NULL, check.names = FALSE)
```

```r
  colnames(out) <- c("gene", paste0("mean_", region1), paste0("mean_", ref_name),
                     "log2FC", "W", "p.value", "p.adj")
  out <- out[order(out$p.value), ]
  rownames(out) <- NULL
  out
}

# ---- Example (simulated data; select this block and run) --------------------
if (FALSE) {
  set.seed(1)
  expr <- matrix(rnorm(200 * 60), nrow = 200,
                 dimnames = list(paste0("Gene", 1:200), paste0("Spot", 1:60)))
  region <- rep(c("core", "boundary", "stroma"), each = 20)
  expr["Gene1", region == "core"] <- expr["Gene1", region == "core"] + 3   # DE up in core
  res  <- spatial_region_de(expr, region, region1 = "core")              # core vs rest
  res2 <- spatial_region_de(expr, region, region1 = "core", region2 = "stroma")
  head(res)
}
# ==============================================================================
# spatial_score_plot()  —  Spatial map of a per-spot signature score
#
# Method : Draw spots at their tissue coordinates and colour them by a continuous
#          per-spot signature score (e.g. the output of a gene-set scoring step)
#          with ggplot2  —  geom_point over coord_fixed(ratio = 1), a viridis
#          colour scale, and a stripped-down theme. A custom renderer sharing the
#          spatial layout of the other maps in this folder, specialised for
#          signature scores.
# Input  : data data.frame with columns x, y, score (one row per spot).
# Output : a ggplot object (the caller prints it or saves with ggplot2::ggsave).
# Depends: ggplot2.
# ==============================================================================

#' Spatial map of a per-spot signature score
#'
#' @param data data.frame with columns \code{x}, \code{y}, \code{score} (one row
#'   per spot).
#' @param pt_size point size (default 1.6).
#' @param flip_y reverse the y axis so the map matches image coordinates, whose
#'   origin is at the top-left (default FALSE).
#' @param option viridis colour-map option passed to
#'   \code{\link[ggplot2]{scale_colour_viridis_c}} (default "magma").
#' @param legend_title legend title (default "score").
#' @return a \code{ggplot} object.
spatial_score_plot <- function(data, pt_size = 1.6, flip_y = FALSE,
                               option = "magma", legend_title = "score") {
  d <- as.data.frame(data)
  if (!all(c("x", "y", "score") %in% colnames(d)))
    stop("`data` must have columns x, y, score.")
  d <- data.frame(x = as.numeric(d$x), y = as.numeric(d$y),
                  score = as.numeric(d$score))

  p <- ggplot2::ggplot(d, ggplot2::aes(x = x, y = y, colour = score)) +
    ggplot2::geom_point(size = pt_size, stroke = 0) +
    ggplot2::scale_colour_viridis_c(option = option) +
    ggplot2::coord_fixed(ratio = 1) +
    ggplot2::labs(colour = legend_title) +
    ggplot2::theme_minimal(base_size = 12) +
```

```
    ggplot2::theme(panel.grid    = ggplot2::element_blank(),
                   axis.text     = ggplot2::element_blank(),
                   axis.ticks    = ggplot2::element_blank(),
                   axis.title    = ggplot2::element_blank())
  if (isTRUE(flip_y)) p <- p + ggplot2::scale_y_reverse()
  p
}

# ---- Example (simulated data; select this block and run) --------------------
if (FALSE) {
  library(ggplot2)
  set.seed(1)
  n <- 500
  d <- data.frame(x = runif(n, 0, 30), y = runif(n, 0, 30))
  d$score <- (d$x + d$y) / 60 + rnorm(n, sd = 0.05)      # diagonal signature gradient
  p <- spatial_score_plot(d)
  inherits(p, "ggplot")
}
# ==============================================================================
# estimate_score()  —  Stromal / immune / ESTIMATE scores and tumour purity
#
# Method : Run the ESTIMATE algorithm (Yoshihara et al., 2013) on an expression
#          matrix. Genes are first restricted to the ESTIMATE common-gene set
#          (estimate::filterCommonGenes), then single-sample stromal and immune
#          signatures are scored and combined into an ESTIMATE score
#          (estimate::estimateScore); for Affymetrix data the algorithm also
#          derives tumour purity from the ESTIMATE score. The estimate package
#          communicates through GCT files, so the matrix is written to a
#          temporary file, processed, and the scores are read back and returned
#          as a table (temporary files are removed on exit).
# Input  : expr       numeric matrix, genes in rows (rownames = official gene
#                     symbols), samples in columns.
#          platform   microarray platform passed to estimateScore: "affymetrix"
#                     (default), "agilent" or "illumina". Tumour purity is only
#                     defined for "affymetrix".
# Output : data.frame(sample, StromalScore, ImmuneScore, ESTIMATEScore,
#          TumorPurity); the TumorPurity column is present only for
#          "affymetrix". Compute only  —  no plotting.
# Depends: estimate (install from R-Forge:
#          install.packages("estimate", repos = "http://r-forge.r-project.org")).
# ==============================================================================

#' ESTIMATE stromal / immune / ESTIMATE scores and tumour purity
#'
#' @param expr numeric matrix (genes x samples); rownames are official gene
#'   symbols (required so genes intersect the ESTIMATE common-gene set).
#' @param platform microarray platform for \code{estimate::estimateScore}:
#'   "affymetrix" (default), "agilent" or "illumina". Tumour purity is only
#'   computed for "affymetrix".
#' @return data.frame with one row per sample and columns \code{StromalScore},
#'   \code{ImmuneScore}, \code{ESTIMATEScore}, and (Affymetrix only)
#'   \code{TumorPurity}.
estimate_score <- function(expr,
                           platform = c("affymetrix", "agilent", "illumina")) {
  platform <- match.arg(platform)
  if (!requireNamespace("estimate", quietly = TRUE))
    stop("Package 'estimate' is required (install from R-Forge).")
```

```r
  expr <- as.matrix(expr)
  if (is.null(rownames(expr)))
    stop("`expr` must have rownames (gene symbols).")
  if (is.null(colnames(expr)))
    stop("`expr` must have colnames (sample IDs).")

  in_expr     <- tempfile(fileext = ".txt")
  common_gct <- tempfile(fileext = ".gct")
  score_gct   <- tempfile(fileext = ".gct")
  on.exit(unlink(c(in_expr, common_gct, score_gct)), add = TRUE)

  utils::write.table(expr, file = in_expr, sep = "\t",
                     quote = FALSE, col.names = NA)
  estimate::filterCommonGenes(input.f = in_expr, output.f = common_gct,
                              id = "GeneSymbol")
  estimate::estimateScore(input.ds = common_gct, output.ds = score_gct,
                          platform = platform)

  # The output GCT holds score rows x (Description + samples) columns; drop the
  # Description column and transpose to one row per sample.
  sc <- utils::read.delim(score_gct, skip = 2, header = TRUE,
                          row.names = 1, check.names = FALSE)
  sc$Description <- NULL
  sc <- as.data.frame(t(as.matrix(sc)), check.names = FALSE)
  data.frame(sample = rownames(sc), sc, row.names = NULL, check.names = FALSE)
}

# ---- Example (simulated data; requires the 'estimate' package) --------------
if (FALSE) {
  if (requireNamespace("estimate", quietly = TRUE)) {
    set.seed(1)
    # NOTE: a real run needs official gene symbols as rownames so that genes
    # intersect the ESTIMATE common-gene set; the placeholder IDs below only
    # illustrate the call signature.
    genes <- paste0("Gene", 1:2000)
    expr   <- matrix(rnorm(2000 * 20, mean = 8, sd = 2), nrow = 2000,
                     dimnames = list(genes, paste0("S", 1:20)))
    scores <- estimate_score(expr, platform = "affymetrix")
    head(scores)
  }
}
# ============================================================================
# immune_deconvolution()  —  Immune cell-type deconvolution wrapper
#
# Method : Thin wrapper over immunedeconv::deconvolute(), which estimates
#          immune / stromal cell-type abundances from a bulk expression matrix.
#          Only open, freely redistributable methods are exposed here:
#          "mcp_counter", "epic", "quantiseq" and "xcell". Most methods expect
#          non-log, linear-scale expression (TPM is recommended) with official
#          gene symbols as rownames; consult the immunedeconv documentation for
#          the scale, normalization and interpretation each method assumes.
# Input  : expr     numeric matrix, genes in rows (rownames = gene symbols),
#                   samples in columns; linear-scale (e.g. TPM) expression.
#          method   one of "mcp_counter", "epic", "quantiseq", "xcell".
# Output : data.frame with a `cell_type` column followed by one column per
#          sample, holding the estimated scores. Compute only  —  no plotting.
# Depends: immunedeconv (GitHub: omnideconv/immunedeconv). Individual methods
```

```
#                 carry their own licences (e.g. EPIC, quanTIseq and xCell are free for
#                 academic / non-commercial use only)  —  check before commercial use.
# ==============================================================================

#' Immune cell-type deconvolution (open methods)
#'
#' @param expr numeric matrix (genes x samples); rownames are gene symbols,
#'     linear-scale expression (TPM recommended for most methods).
#' @param method deconvolution method, one of "mcp_counter" (default), "epic",
#'     "quantiseq", "xcell". Each estimates cell-type abundances differently and
#'     is interpreted on its own scale.
#' @param ... further arguments forwarded to
#'     \code{\link[immunedeconv]{deconvolute}}.
#' @return data.frame; first column \code{cell_type}, remaining columns one per
#'     sample, holding the method's abundance scores.
immune_deconvolution <- function(expr,
                                 method = c("mcp_counter", "epic",
                                            "quantiseq", "xcell"), ...) {
  method <- match.arg(method)
  if (!requireNamespace("immunedeconv", quietly = TRUE))
    stop("Package 'immunedeconv' is required.")
  expr <- as.matrix(expr)
  if (is.null(rownames(expr)))
    stop("`expr` must have rownames (gene symbols).")
  if (is.null(colnames(expr)))
    stop("`expr` must have colnames (sample IDs).")

  res <- immunedeconv::deconvolute(expr, method = method, ...)
  as.data.frame(res, check.names = FALSE)
}

# ---- Example (simulated data; requires the 'immunedeconv' package) ----------
if (FALSE) {
  if (requireNamespace("immunedeconv", quietly = TRUE)) {
    set.seed(1)
    # NOTE: real runs need official gene symbols as rownames so a method's
    # signature genes can be matched; the placeholder IDs below only illustrate
    # the call signature.
    genes <- paste0("Gene", 1:1000)
    expr    <- matrix(2^runif(1000 * 12, 0, 12), nrow = 1000,      # linear-scale
                      dimnames = list(genes, paste0("S", 1:12)))
    fractions <- immune_deconvolution(expr, method = "quantiseq")
    head(fractions)
  }
}
# ==============================================================================
# ssgsea_immune_signature()  —  Per-sample ssGSEA scoring of immune signatures
#
# Method : Score user-supplied immune-cell / immune-pathway gene signatures in
#                 every sample by single-sample GSEA (Barbie et al., 2009), via
#                 GSVA::gsva() with a GSVA::ssgseaParam() object. Within each sample the
#                 genes are ranked and an enrichment score is accumulated per signature
#                 from the rank-weighted difference between in-set and out-of-set
#                 genes. Because the statistic is rank-based within each sample, pass
#                 the FULL expression matrix (the whole gene background), not a matrix
#                 pre-subset to signature genes. No signature list is bundled  —  you
#                 supply your own (e.g. immune-cell marker sets from a publication or a
```

```r
#                  curated collection).
# Input    : expr               numeric matrix, genes in rows (rownames = gene symbols),
#                                    samples in columns; the complete expression background.
#                  signatures    named list() of character vectors (signature name ->
#                                    gene symbols); supplied by the user.
# Output : numeric matrix, signatures in rows, samples in columns, of ssGSEA
#                  enrichment scores. Compute only — no plotting.
# Depends: GSVA (Bioconductor); uses the modern ssgseaParam() object API.
# ==============================================================================

#' Per-sample ssGSEA scoring of user-supplied immune signatures
#'
#' @param expr numeric matrix (genes x samples); rownames are gene symbols.
#'     Pass the complete expression matrix, not a signature-only subset.
#' @param signatures named \code{list} of character vectors; each element is one
#'     immune signature (names become the output rownames). Supply your own — no
#'     gene sets are embedded in this function.
#' @param alpha exponent weighting the rank statistic in the enrichment score
#'     (default 0.25, the ssGSEA convention).
#' @param normalize if TRUE (default), scale scores as in the original ssGSEA
#'     implementation.
#' @param min_size,max_size drop signatures whose number of genes found in
#'     \code{expr} falls outside \code{[min_size, max_size]} (default 1, Inf).
#' @return numeric matrix (signatures x samples) of enrichment scores.
ssgsea_immune_signature <- function(expr, signatures, alpha = 0.25,
                                    normalize = TRUE,
                                    min_size = 1, max_size = Inf) {
  if (!requireNamespace("GSVA", quietly = TRUE))
    stop("Package 'GSVA' is required.")
  expr <- as.matrix(expr)
  if (is.null(rownames(expr)))
    stop("`expr` must have rownames (gene symbols).")
  if (!is.list(signatures) || is.null(names(signatures)))
    stop("`signatures` must be a named list of character vectors.")

  par <- GSVA::ssgseaParam(exprData = expr, geneSets = signatures,
                           alpha = alpha, normalize = normalize,
                           minSize = min_size, maxSize = max_size)
  GSVA::gsva(par, verbose = FALSE)
}

# ---- Example (simulated data; select this block and run) --------------------
if (FALSE) {
  library(GSVA)
  set.seed(1)
  expr <- matrix(rnorm(800 * 40), nrow = 800,
                 dimnames = list(paste0("Gene", 1:800), paste0("S", 1:40)))
  # User-supplied signatures (placeholder gene IDs; use your own marker sets).
  signatures <- list(signature_A = paste0("Gene", 1:40),
                     signature_B = paste0("Gene", 100:160),
                     signature_C = paste0("Gene", 300:340))
  scores <- ssgsea_immune_signature(expr, signatures)
  dim(scores)            # 3 signatures x 40 samples
  scores[, 1:4]
}
# ==============================================================================
# tme_gene_vs_infiltration_correlation() — Gene expression vs each immune /
```

```r
#                                                         stromal cell-type score
#
# Method : Correlate one gene's expression against each cell-type column of a
#          per-sample infiltration matrix (for example the output of an immune
#          deconvolution tool). Samples are aligned by name — names of the gene
#          vector against rownames of the infiltration matrix — and one
#          stats::cor.test is run per cell type (Spearman by default) on the
#          shared samples where both values are finite. Benjamini-Hochberg
#          adjustment is applied across cell types.
# Input  : gene           named numeric vector; names = sample IDs, values =
#                         expression of the gene of interest.
#          infiltration   samples x cell_types numeric matrix / data.frame;
#                         rownames = sample IDs, one column per cell type.
#          method         "spearman" (default) or "pearson".
# Output : data.frame(cell_type, rho, p.value, p.adj, n) ordered by p.value.
#          Compute only — no plotting.
# Depends: base R only.
# ============================================================================

#' Correlate a gene against each cell-type score of an infiltration matrix
#'
#' @param gene named numeric vector of expression; names are sample IDs.
#' @param infiltration samples x cell_types numeric matrix or data.frame;
#'    rownames are sample IDs, columns are cell types. Supply your own scores
#'    (e.g. from CIBERSORT, MCP-counter, EPIC, quanTIseq, xCell or ssGSEA).
#' @param method correlation method, "spearman" (default) or "pearson".
#' @param p_adjust multiple-testing method for \code{\link[stats]{p.adjust}}
#'    (default "BH").
#' @param min_pairs minimum complete (both-finite) sample pairs per cell type
#'    (default 3).
#' @return data.frame, one row per cell type, ordered by \code{p.value}. The
#'    \code{rho} column holds the correlation coefficient for the chosen method.
tme_gene_vs_infiltration_correlation <- function(gene, infiltration,
                                                 method = c("spearman", "pearson"),
                                                 p_adjust = "BH", min_pairs = 3) {
  method <- match.arg(method)
  infiltration <- as.matrix(infiltration)
  if (is.null(names(gene)) || is.null(rownames(infiltration)))
    stop("`gene` must be named by sample and `infiltration` must have rownames.")
  common <- intersect(names(gene), rownames(infiltration))
  if (length(common) < min_pairs)
    stop("only ", length(common), " shared samples; need >= ", min_pairs, ".")
  g     <- as.numeric(gene[common])
  mat   <- infiltration[common, , drop = FALSE]
  cells <- colnames(mat)
  if (is.null(cells)) cells <- paste0("cell_type", seq_len(ncol(mat)))

  stat <- t(vapply(seq_len(ncol(mat)), function(j) {
    y  <- as.numeric(mat[, j])
    ok <- is.finite(g) & is.finite(y)
    n  <- sum(ok)
    if (n < min_pairs) return(c(rho = NA_real_, p = NA_real_, n = n))
    ht <- suppressWarnings(stats::cor.test(g[ok], y[ok], method = method))
    c(rho = unname(ht$estimate), p = ht$p.value, n = n)
  }, numeric(3)))

  out <- data.frame(cell_type = cells, rho = stat[, "rho"],
```

```
                          p.value = stat[, "p"],
                          p.adj = stats::p.adjust(stat[, "p"], method = p_adjust),
                          n = stat[, "n"], row.names = NULL, stringsAsFactors = FALSE)
  out <- out[order(out$p.value), ]
  rownames(out) <- NULL
  out
}

# ---- Example (simulated data; select this block and run) --------------------
if (FALSE) {
  set.seed(1)
  samples      <- paste0("S", 1:50)
  gene         <- setNames(rnorm(50), samples)
  infiltration <- matrix(rnorm(50 * 6), nrow = 50,
                         dimnames = list(samples, paste0("CellType", 1:6)))
  infiltration[, 1] <- 0.7 * gene + rnorm(50, sd = 0.5)   # CellType1 tracks gene
  res <- tme_gene_vs_infiltration_correlation(gene, infiltration)
  res
}
# =============================================================================
# tme_high_low_infiltration_diff()  —  Cell-type infiltration in gene-High vs
#                                      gene-Low samples
#
# Method : Split samples into High and Low groups at the median expression of
#          one gene, then compare each cell-type score between the two groups
#          with a two-sided Wilcoxon rank-sum test (stats::wilcox.test).
#          Samples are aligned by name (names of the gene vector against
#          rownames of the infiltration matrix); Benjamini-Hochberg adjustment
#          is applied across cell types. Samples whose expression equals the
#          median are assigned to the Low group (value <= median).
# Input  : gene          named numeric vector; names = sample IDs, values =
#                        expression of the gene of interest.
#          infiltration  samples x cell_types numeric matrix / data.frame;
#                        rownames = sample IDs, one column per cell type.
# Output : data.frame(cell_type, median_high, median_low, p.value, p.adj)
#          ordered by p.value. Compute only  —  no plotting.
# Depends: base R only.
# =============================================================================

#' Compare each cell-type score between gene-High and gene-Low samples
#'
#' @param gene named numeric vector of expression; names are sample IDs. The
#'   grouping gene; samples are split High/Low at its median.
#' @param infiltration samples x cell_types numeric matrix or data.frame;
#'   rownames are sample IDs, columns are cell types. Supply your own scores.
#' @param p_adjust multiple-testing method for \code{\link[stats]{p.adjust}}
#'   (default "BH").
#' @param min_per_group minimum finite samples required in each group to test a
#'   cell type (default 3).
#' @return data.frame, one row per cell type, ordered by \code{p.value}, giving
#'   the median score in each group and the Wilcoxon p-value / adjusted p-value.
tme_high_low_infiltration_diff <- function(gene, infiltration,
                                           p_adjust = "BH", min_per_group = 3) {
  infiltration <- as.matrix(infiltration)
  if (is.null(names(gene)) || is.null(rownames(infiltration)))
    stop("`gene` must be named by sample and `infiltration` must have rownames.")
  common <- intersect(names(gene), rownames(infiltration))
```

```r
  if (length(common) < 2L * min_per_group)
    stop("only ", length(common), " shared samples; need >= ",
         2L * min_per_group, ".")
  g     <- as.numeric(gene[common])
  mat <- infiltration[common, , drop = FALSE]
  if (!all(is.finite(g)))
    stop("`gene` has non-finite values on the shared samples.")

  med   <- stats::median(g)
  high <- g >  med
  low   <- g <= med
  if (sum(high) < min_per_group || sum(low) < min_per_group)
    stop("median split leaves fewer than ", min_per_group,
         " samples in a group (ties at the median?).")

  cells <- colnames(mat)
  if (is.null(cells)) cells <- paste0("cell_type", seq_len(ncol(mat)))

  stat <- t(vapply(seq_len(ncol(mat)), function(j) {
    y   <- as.numeric(mat[, j])
    xh <- y[high]; xh <- xh[is.finite(xh)]
    xl <- y[low];  xl <- xl[is.finite(xl)]
    if (length(xh) < min_per_group || length(xl) < min_per_group)
      return(c(mh = NA_real_, ml = NA_real_, p = NA_real_))
    ht <- suppressWarnings(stats::wilcox.test(xh, xl))
    c(mh = stats::median(xh), ml = stats::median(xl), p = ht$p.value)
  }, numeric(3)))

  out <- data.frame(cell_type = cells,
                    median_high = stat[, "mh"], median_low = stat[, "ml"],
                    p.value = stat[, "p"],
                    p.adj = stats::p.adjust(stat[, "p"], method = p_adjust),
                    row.names = NULL, stringsAsFactors = FALSE)
  out <- out[order(out$p.value), ]
  rownames(out) <- NULL
  out
}

# ---- Example (simulated data; select this block and run) --------------------
if (FALSE) {
  set.seed(1)
  samples      <- paste0("S", 1:60)
  gene         <- setNames(rnorm(60), samples)
  infiltration <- matrix(rnorm(60 * 5), nrow = 60,
                         dimnames = list(samples, paste0("CellType", 1:5)))
  # CellType1 is higher in the gene-High samples
  hi <- gene > median(gene)
  infiltration[hi, 1] <- infiltration[hi, 1] + 1.5
  res <- tme_high_low_infiltration_diff(gene, infiltration)
  res
}
# ============================================================================
# ensemble_signature_compare()  –  Compare prognostic-signature builders
#
# Method : Build a prognostic risk score with several Cox signature methods and
#          compare their discrimination. For each requested method a signature is
#          fit on the training data, the linear risk score X %*% coef is formed,
```

```r
#                   and Harrell's C-index is computed on the training set and (optionally)
#                   on an independent validation set. Methods (each composed inline, so
#                   this file stays self-contained):
#                       - "lasso"      : cv.glmnet Cox, alpha = 1, coefs at lambda.min;
#                       - "ridge"      : cv.glmnet Cox, alpha = 0, all coefs at lambda.min;
#                       - "stepcox" : full coxph then MASS::stepAIC(direction = "both").
# Input    : x              training matrix, samples in rows, genes in columns.
#                   y              training survival data.frame (time > 0, status 1/0).
#                   x_valid    optional validation matrix (same genes as columns).
#                   y_valid    optional validation survival data.frame.
# Output : data.frame(method, n_features, cindex_train, cindex_valid)  —  one row
#                   per method. Compute only  —  no plotting.
# Depends: glmnet, survival, MASS.
# ==============================================================================

#' Compare prognostic-signature builders by C-index (compute only)
#'
#' @param x training numeric matrix (samples x genes); colnames are gene symbols.
#' @param y training survival data.frame aligned to \code{x} rows.
#' @param x_valid optional validation matrix; genes matched to \code{x} by
#'      column name. If NULL, \code{cindex_valid} is NA.
#' @param y_valid optional validation survival data.frame aligned to
#'      \code{x_valid}.
#' @param time,status survival column names in \code{y}/\code{y_valid} (defaults
#'      "time", "status"); \code{status} must be 1 = event, 0 = censored.
#' @param methods character vector; any of "lasso", "ridge", "stepcox" (default
#'      all three).
#' @param nfolds cross-validation folds for the glmnet methods (default 10).
#' @param seed integer seed set before each glmnet cross-validation for
#'      reproducibility (default 1).
#' @return data.frame with columns \code{method}, \code{n_features},
#'      \code{cindex_train} and \code{cindex_valid}.
ensemble_signature_compare <- function(x, y, x_valid = NULL, y_valid = NULL,
                                                                time = "time", status = "status",
                                                                methods = c("lasso", "ridge", "stepcox"),
                                                                nfolds = 10, seed = 1) {
  methods <- match.arg(methods, several.ok = TRUE)

  # ---- clean a (matrix, survival df) pair into (x, Surv) -------------------
  prep <- function(xm, yd) {
    xm <- as.matrix(xm)
    if (!all(c(time, status) %in% names(yd)))
      stop("survival data.frame must contain columns: ", time, ", ", status)
    if (nrow(xm) != nrow(yd)) stop("nrow(x) must equal nrow(y).")
    if (is.null(colnames(xm))) colnames(xm) <- paste0("gene", seq_len(ncol(xm)))
    tt <- as.numeric(yd[[time]]); st <- as.integer(yd[[status]])
    ok <- is.finite(tt) & tt > 0 & st %in% c(0L, 1L) & stats::complete.cases(xm)
    list(x = xm[ok, , drop = FALSE],
          surv = survival::Surv(tt[ok], st[ok]))
  }

  tr <- prep(x, y)
  if (nrow(tr$x) < 2L * nfolds)
    stop("too few complete training samples for ", nfolds, "-fold CV.")
  have_valid <- !is.null(x_valid) && !is.null(y_valid)
  va <- if (have_valid) prep(x_valid, y_valid) else NULL
```

```
  # Harrell's C-index of a risk score against a Surv object. reverse = TRUE
  # uses the risk convention (larger score -> shorter survival time).
  cindex <- function(surv, risk) {
    d <- data.frame(risk = risk); d$surv <- surv
    unname(survival::concordance(surv ~ risk, data = d,
                                 reverse = TRUE)$concordance)
  }

  # Fit one method on training data -> named coefficient vector on gene space.
  fit_coef <- function(m) {
    if (m %in% c("lasso", "ridge")) {
      a <- if (m == "lasso") 1 else 0
      set.seed(seed)
      cv <- glmnet::cv.glmnet(tr$x, tr$surv, family = "cox", alpha = a,
                              nfolds = nfolds)
      cf <- as.matrix(stats::coef(cv, s = "lambda.min"))[, 1]
      cf[cf != 0]
    } else {                                          # stepcox
      work <- as.data.frame(tr$x, stringsAsFactors = FALSE)
      work$.surv <- tr$surv
      full <- survival::coxph(.surv ~ ., data = work)
      step <- MASS::stepAIC(full, direction = "both", trace = FALSE)
      stats::coef(step)
    }
  }

  rows <- lapply(methods, function(m) {
    cf <- fit_coef(m)
    cf <- cf[is.finite(cf) & cf != 0]
    if (length(cf) == 0L)
      return(data.frame(method = m, n_features = 0L,
                        cindex_train = NA_real_, cindex_valid = NA_real_))
    g <- names(cf)
    r_tr <- as.numeric(tr$x[, g, drop = FALSE] %*% cf)
    ci_tr <- cindex(tr$surv, r_tr)

    ci_va <- NA_real_
    if (have_valid) {
      g2 <- intersect(g, colnames(va$x))
      if (length(g2) > 0L) {
        r_va <- as.numeric(va$x[, g2, drop = FALSE] %*% cf[g2])
        ci_va <- cindex(va$surv, r_va)
      }
    }
    data.frame(method = m, n_features = length(cf),
               cindex_train = ci_tr, cindex_valid = ci_va)
  })

  out <- do.call(rbind, rows)
  rownames(out) <- NULL
  out
}

# ---- Example (simulated data; select this block and run) --------------------
if (FALSE) {
  set.seed(1)
  sim <- function(n, p, beta) {
```

```
    x <- matrix(rnorm(n * p), n, p, dimnames = list(NULL, paste0("Gene", 1:p)))
    risk <- as.numeric(x %*% beta)
    time      <- rexp(n, rate = 0.1 * exp(risk))
    status <- rbinom(n, 1, 0.7)
    list(x = x, y = data.frame(time = time, status = status))
  }
  p <- 20; beta <- c(rep(0.8, 4), rep(0, p - 4))               # first 4 genes prognostic
  tr <- sim(200, p, beta)
  va <- sim(150, p, beta)
  res <- ensemble_signature_compare(tr$x, tr$y, va$x, va$y)
  res
}
# ==============================================================================
# risk_score()  —  Linear prognostic risk score from a signature
#
# Method : Compute a per-sample linear risk score as the sum over signature
#                 genes of expression times coefficient, score_s = sum_i expr[i, s] *
#                 coef_i. This is the standard way to turn a fitted Cox signature (e.g.
#                 from LASSO / elastic-net / boosted Cox) into a single number per
#                 sample for downstream stratification. Only genes present in both the
#                 coefficient table and the expression matrix are used; missing
#                 signature genes are reported via a warning.
# Input    : expr    numeric matrix, genes in rows (rownames = gene symbols),
#                          samples in columns; same scale used when the coefficients were
#                          estimated.
#                 coef    data.frame with a gene-name column and a coefficient column.
# Output : named numeric vector, one risk score per sample (names = colnames of
#                 expr). Compute only  —  no plotting.
# Depends: base R only.
# ==============================================================================

#' Linear prognostic risk score from a signature (compute only)
#'
#' @param expr numeric matrix (genes x samples); rownames are gene symbols.
#' @param coef data.frame holding the signature coefficients.
#' @param gene name of the gene-symbol column in \code{coef} (default "gene").
#' @param coef_col name of the coefficient column in \code{coef} (default
#'      "coef").
#' @return a named numeric vector of risk scores, one per column of \code{expr}.
risk_score <- function(expr, coef, gene = "gene", coef_col = "coef") {
  expr <- as.matrix(expr)
  if (!all(c(gene, coef_col) %in% names(coef)))
    stop("`coef` must contain columns: ", gene, ", ", coef_col)
  if (is.null(rownames(expr)))
    stop("`expr` must have gene symbols as rownames.")

  g <- as.character(coef[[gene]])
  b <- as.numeric(coef[[coef_col]])
  keep <- !is.na(g) & !is.na(b) & nzchar(g)
  g <- g[keep]; b <- b[keep]

  common <- intersect(g, rownames(expr))
  if (length(common) == 0L)
    stop("none of the signature genes are present in rownames(expr).")
  if (length(common) < length(g))
    warning(length(g) - length(common), " of ", length(g),
                 " signature genes are missing from expr and were dropped.")
```

```r
  b_common <- b[match(common, g)]
  sub <- expr[common, , drop = FALSE]
  score <- as.numeric(crossprod(sub, b_common))      # t(sub) %*% b, samples x 1
  names(score) <- colnames(expr)
  score
}

# ---- Example (simulated data; select this block and run) --------------------
if (FALSE) {
  set.seed(1)
  genes     <- paste0("Gene", 1:50)
  samples <- paste0("S", 1:30)
  expr <- matrix(rnorm(length(genes) * length(samples)),
                 nrow = length(genes), dimnames = list(genes, samples))
  coef <- data.frame(gene = c("Gene1", "Gene2", "Gene3"),
                     coef = c(0.8, -0.5, 1.2))
  scores <- risk_score(expr, coef)
  head(scores)
}
# =============================================================================
# signature_cindex() — Harrell's C-index of a risk score
#
# Method : Harrell's concordance index for a continuous risk score under right
#          censoring, via survival::concordance(Surv(time, status) ~ risk). The
#          C-index is the probability that, for a comparable pair of subjects,
#          the one with the higher risk score has the shorter survival time; 0.5
#          is chance and 1.0 is perfect discrimination. The standard error is
#          returned from the concordance variance so a Wald confidence interval
#          can be formed by the caller.
# Input  : df   data.frame with columns time (follow-up), status (1 = event,
#                 0 = censored) and a continuous risk-score column.
# Output : list(c_index, se, n, n_event). Compute only — no plotting.
# Depends: survival.
# =============================================================================

#' Harrell's C-index of a risk score (compute only)
#'
#' @param df data.frame with the time, status and risk columns.
#' @param time,status,risk column names in \code{df} (defaults "time", "status",
#'   "risk"). \code{status} must be 1 = event, 0 = censored, and higher
#'   \code{risk} is expected to mean higher hazard.
#' @return a list with the \code{c_index}, its standard error \code{se}, the
#'   number of usable samples \code{n} and the number of events \code{n_event}.
signature_cindex <- function(df, time = "time", status = "status",
                             risk = "risk") {
  for (col in c(time, status, risk))
    if (!col %in% names(df)) stop("column not found: ", col)

  d <- data.frame(time     = as.numeric(df[[time]]),
                  status = as.integer(df[[status]]),
                  risk     = as.numeric(df[[risk]]))
  d <- d[stats::complete.cases(d) & d$time > 0, , drop = FALSE]
  if (nrow(d) < 5L) stop("need >= 5 complete samples with positive time.")
  if (sum(d$status) < 2L) stop("need >= 2 events to estimate concordance.")

  # reverse = TRUE encodes the risk convention: a larger score predicts a
```

```
  # shorter survival time (matches concordance() applied to a coxph fit).
  cc <- survival::concordance(survival::Surv(time, status) ~ risk, data = d,
                              reverse = TRUE)

  list(c_index = unname(cc$concordance),
       se      = unname(sqrt(cc$var)),
       n       = nrow(d),
       n_event = sum(d$status))
}

# ---- Example (simulated data; select this block and run) --------------------
if (FALSE) {
  set.seed(1)
  n <- 250
  risk   <- rnorm(n)
  time   <- rexp(n, rate = 0.1 * exp(0.9 * risk))      # higher risk, shorter time
  status <- rbinom(n, 1, 0.7)
  df <- data.frame(time = time, status = status, risk = risk)
  res <- signature_cindex(df)
  res$c_index; res$se
}
# ==============================================================================
# signature_coxboost()  —  Boosted Cox prognostic signature (CoxBoost)
#
# Method : Likelihood-based componentwise boosting for the Cox model (CoxBoost).
#          The optimal penalty is located with optimCoxBoostPenalty, the optimal
#          number of boosting steps at that penalty is chosen by cross-validation
#          with cv.CoxBoost, and the final model is fit with CoxBoost at that
#          step/penalty. Because each boosting step updates a single component,
#          many genes keep a zero coefficient; the genes with non-zero
#          coefficients at the final step form the signature.
# Input  : x   numeric matrix, samples in rows, genes in columns (colnames = gene
#              symbols); typically log-scale expression.
#          y   survival data.frame with columns time (> 0) and status (1 = event,
#              0 = censored), rows aligned to x.
# Output : list(coef = data.frame(gene, coef) for non-zero genes ordered by
#          |coef|, penalty, optimal_step, n_selected). Compute only.
# Depends: CoxBoost.
# ==============================================================================

#' Boosted Cox prognostic signature via CoxBoost (compute only)
#'
#' @param x numeric matrix (samples x genes); colnames are gene symbols.
#' @param y survival data.frame aligned to \code{x} rows.
#' @param time,status column names in \code{y} (defaults "time", "status");
#'   \code{status} must be 1 = event, 0 = censored.
#' @param maxstepno maximum number of boosting steps searched by cross-validation
#'   (default 200).
#' @param seed integer seed set before the cross-validation folds are drawn, for
#'   reproducibility (default 1).
#' @return a list with the non-zero coefficient table (\code{gene}, \code{coef}),
#'   the selected \code{penalty}, the \code{optimal_step}, and \code{n_selected}.
signature_coxboost <- function(x, y, time = "time", status = "status",
                               maxstepno = 200, seed = 1) {
  if (!requireNamespace("CoxBoost", quietly = TRUE))
    stop("package 'CoxBoost' is required.")
  x <- as.matrix(x)
```

```
  if (!all(c(time, status) %in% names(y)))
    stop("`y` must contain columns: ", time, ", ", status)
  if (nrow(x) != nrow(y))
    stop("nrow(x) must equal nrow(y) (samples aligned row-wise).")
  if (is.null(colnames(x))) colnames(x) <- paste0("gene", seq_len(ncol(x)))

  tt <- as.numeric(y[[time]])
  st <- as.integer(y[[status]])
  ok <- is.finite(tt) & tt > 0 & st %in% c(0L, 1L) & stats::complete.cases(x)
  x   <- x[ok, , drop = FALSE]; tt <- tt[ok]; st <- st[ok]
  if (sum(st) < 5L) stop("need >= 5 events to fit CoxBoost.")

  set.seed(seed)
  pen <- CoxBoost::optimCoxBoostPenalty(time = tt, status = st, x = x,
                                        maxstepno = maxstepno, trace = FALSE)
  cv   <- CoxBoost::cv.CoxBoost(time = tt, status = st, x = x,
                                penalty = pen$penalty, maxstepno = maxstepno,
                                trace = FALSE)
  fit <- CoxBoost::CoxBoost(time = tt, status = st, x = x,
                            stepno = cv$optimal.step, penalty = pen$penalty)

  cf <- stats::coef(fit)
  if (is.null(names(cf))) names(cf) <- colnames(x)
  nz <- cf[cf != 0]
  cdf <- data.frame(gene = names(nz), coef = as.numeric(nz), row.names = NULL)
  cdf <- cdf[order(-abs(cdf$coef)), , drop = FALSE]
  rownames(cdf) <- NULL

  list(coef = cdf, penalty = pen$penalty, optimal_step = cv$optimal.step,
       n_selected = nrow(cdf))
}

# ---- Example (simulated data; select this block and run) --------------------
if (FALSE) {
  set.seed(1)
  n <- 150; p <- 25
  x <- matrix(rnorm(n * p), n, p, dimnames = list(NULL, paste0("Gene", 1:p)))
  beta <- c(rep(0.9, 4), rep(0, p - 4))                  # first 4 genes prognostic
  risk <- as.numeric(x %*% beta)
  time    <- rexp(n, rate = 0.1 * exp(risk))
  status <- rbinom(n, 1, 0.7)
  y <- data.frame(time = time, status = status)
  res <- signature_coxboost(x, y)
  res$coef; res$optimal_step
}
# ==============================================================================
# signature_elasticnet_cox()  –  Elastic-net-penalized Cox prognostic signature
#
# Method : Elastic-net Cox proportional-hazards regression: glmnet with
#          family = "cox" and a mixing parameter alpha in [0, 1] (0.5 by default)
#          that blends the LASSO (L1) and ridge (L2) penalties. The penalty
#          strength lambda is chosen by k-fold cross-validation (cv.glmnet,
#          partial-likelihood deviance); genes with non-zero coefficients at
#          lambda.min form the signature. Compared with pure LASSO, the L2 term
#          stabilizes selection among correlated genes.
# Input  : x   numeric matrix, samples in rows, genes in columns (colnames = gene
#              symbols); typically log-scale expression.
```

```r
#                   y    survival data.frame with columns time (> 0) and status (1 = event,
#                        0 = censored), rows aligned to x.
# Output : list(coef = data.frame(gene, coef) for non-zero genes ordered by
#                   |coef|, alpha, lambda_min, n_selected, cvfit). Compute only.
# Depends: glmnet, survival.
# ==============================================================================

#' Elastic-net-penalized Cox prognostic signature (compute only)
#'
#' @param x numeric matrix (samples x genes); colnames are gene symbols.
#' @param y survival data.frame aligned to \code{x} rows.
#' @param time,status column names in \code{y} (defaults "time", "status");
#'    \code{status} must be 1 = event, 0 = censored.
#' @param alpha elastic-net mixing parameter in [0, 1]; 1 = LASSO, 0 = ridge
#'    (default 0.5).
#' @param nfolds number of cross-validation folds (default 10).
#' @param seed integer seed set before cross-validation for a reproducible fold
#'    split and lambda.min (default 1).
#' @return a list with the non-zero coefficient table (\code{gene}, \code{coef}),
#'    \code{alpha}, \code{lambda_min}, \code{n_selected}, and the \code{cv.glmnet}
#'    object.
signature_elasticnet_cox <- function(x, y, time = "time", status = "status",
                                     alpha = 0.5, nfolds = 10, seed = 1) {
  x <- as.matrix(x)
  if (!all(c(time, status) %in% names(y)))
    stop("`y` must contain columns: ", time, ", ", status)
  if (nrow(x) != nrow(y))
    stop("nrow(x) must equal nrow(y) (samples aligned row-wise).")
  if (alpha < 0 || alpha > 1) stop("`alpha` must be in [0, 1].")
  if (is.null(colnames(x))) colnames(x) <- paste0("gene", seq_len(ncol(x)))

  tt <- as.numeric(y[[time]])
  st <- as.integer(y[[status]])
  ok <- is.finite(tt) & tt > 0 & st %in% c(0L, 1L) & stats::complete.cases(x)
  x   <- x[ok, , drop = FALSE]; tt <- tt[ok]; st <- st[ok]
  if (nrow(x) < 2L * nfolds)
    stop("too few complete samples (", nrow(x), ") for ", nfolds, "-fold CV.")

  surv <- survival::Surv(tt, st)
  set.seed(seed)
  cvfit <- glmnet::cv.glmnet(x, surv, family = "cox", alpha = alpha,
                             nfolds = nfolds)

  cf <- as.matrix(stats::coef(cvfit, s = "lambda.min"))
  nz <- cf[cf[, 1] != 0, , drop = FALSE]
  cdf <- data.frame(gene = rownames(nz), coef = as.numeric(nz[, 1]),
                    row.names = NULL)
  cdf <- cdf[order(-abs(cdf$coef)), , drop = FALSE]
  rownames(cdf) <- NULL

  list(coef = cdf, alpha = alpha, lambda_min = cvfit$lambda.min,
       n_selected = nrow(cdf), cvfit = cvfit)
}

# ---- Example (simulated data; select this block and run) --------------------
if (FALSE) {
  set.seed(1)
```

```
  n <- 160; p <- 40
  x <- matrix(rnorm(n * p), n, p, dimnames = list(NULL, paste0("Gene", 1:p)))
  beta <- c(rep(0.8, 6), rep(0, p - 6))                        # first 6 genes prognostic
  risk <- as.numeric(x %*% beta)
  time    <- rexp(n, rate = 0.1 * exp(risk))
  status <- rbinom(n, 1, 0.7)
  y <- data.frame(time = time, status = status)
  res <- signature_elasticnet_cox(x, y, alpha = 0.5)
  res$coef; res$n_selected
}
# ============================================================================
# signature_lasso_cox() — LASSO-penalized Cox prognostic signature
#
# Method : L1-penalized (LASSO) Cox proportional-hazards regression. glmnet is
#          fit with family = "cox" and alpha = 1 along a lambda path; the penalty
#          is chosen by k-fold cross-validation (cv.glmnet, partial-likelihood
#          deviance), and the genes with non-zero coefficients at lambda.min form
#          the signature.
# Input  : x   numeric matrix, samples in rows, genes in columns (colnames = gene
#              symbols); typically log-scale expression.
#          y   survival data.frame with columns time (> 0) and status (1 = event,
#              0 = censored), rows aligned to x.
# Output : list(coef = data.frame(gene, coef) for non-zero genes ordered by
#          |coef|, lambda_min, n_selected, cvfit). Compute only — no plotting.
# Depends: glmnet, survival.
# ============================================================================

#' LASSO-penalized Cox prognostic signature (compute only)
#'
#' @param x numeric matrix (samples x genes); colnames are gene symbols.
#' @param y survival data.frame aligned to \code{x} rows.
#' @param time,status column names in \code{y} (defaults "time", "status");
#'    \code{status} must be 1 = event, 0 = censored.
#' @param nfolds number of cross-validation folds (default 10).
#' @param seed integer seed set before cross-validation so the fold split — and
#'    therefore lambda.min — is reproducible (default 1).
#' @return a list with the non-zero coefficient table (\code{gene}, \code{coef}),
#'    \code{lambda_min}, \code{n_selected}, and the \code{cv.glmnet} object.
signature_lasso_cox <- function(x, y, time = "time", status = "status",
                                nfolds = 10, seed = 1) {
  x <- as.matrix(x)
  if (!all(c(time, status) %in% names(y)))
    stop("`y` must contain columns: ", time, ", ", status)
  if (nrow(x) != nrow(y))
    stop("nrow(x) must equal nrow(y) (samples aligned row-wise).")
  if (is.null(colnames(x))) colnames(x) <- paste0("gene", seq_len(ncol(x)))

  tt <- as.numeric(y[[time]])
  st <- as.integer(y[[status]])
  ok <- is.finite(tt) & tt > 0 & st %in% c(0L, 1L) & stats::complete.cases(x)
  x   <- x[ok, , drop = FALSE]; tt <- tt[ok]; st <- st[ok]
  if (nrow(x) < 2L * nfolds)
    stop("too few complete samples (", nrow(x), ") for ", nfolds, "-fold CV.")

  surv <- survival::Surv(tt, st)
  set.seed(seed)
  cvfit <- glmnet::cv.glmnet(x, surv, family = "cox", alpha = 1, nfolds = nfolds)
```

```
  cf <- as.matrix(stats::coef(cvfit, s = "lambda.min"))
  nz <- cf[cf[, 1] != 0, , drop = FALSE]
  cdf <- data.frame(gene = rownames(nz), coef = as.numeric(nz[, 1]),
                    row.names = NULL)
  cdf <- cdf[order(-abs(cdf$coef)), , drop = FALSE]
  rownames(cdf) <- NULL

  list(coef = cdf, lambda_min = cvfit$lambda.min,
       n_selected = nrow(cdf), cvfit = cvfit)
}

# ---- Example (simulated data; select this block and run) --------------------
if (FALSE) {
  set.seed(1)
  n <- 160; p <- 40
  x <- matrix(rnorm(n * p), n, p, dimnames = list(NULL, paste0("Gene", 1:p)))
  beta <- c(rep(0.8, 5), rep(0, p - 5))                # first 5 genes prognostic
  risk <- as.numeric(x %*% beta)
  time   <- rexp(n, rate = 0.1 * exp(risk))            # higher risk, shorter time
  status <- rbinom(n, 1, 0.7)
  y <- data.frame(time = time, status = status)
  res <- signature_lasso_cox(x, y)
  res$coef; res$lambda_min
}
# ==============================================================================
# signature_ridge_cox()  —  Ridge-penalized Cox prognostic model
#
# Method : L2-penalized (ridge) Cox proportional-hazards regression: glmnet with
#          family = "cox" and alpha = 0. The penalty lambda is chosen by k-fold
#          cross-validation (cv.glmnet, partial-likelihood deviance). Ridge
#          shrinks coefficients toward zero but never sets them exactly to zero,
#          so it does not perform variable selection  —  every gene is retained
#          with a shrunken coefficient. Useful when many correlated genes each
#          contribute a little and dropping any is undesirable.
# Input  : x   numeric matrix, samples in rows, genes in columns (colnames = gene
#              symbols); typically log-scale expression.
#          y   survival data.frame with columns time (> 0) and status (1 = event,
#              0 = censored), rows aligned to x.
# Output : list(coef = data.frame(gene, coef) for all genes ordered by |coef|,
#          lambda_min, n_features, cvfit). Compute only  —  no plotting.
# Depends: glmnet, survival.
# ==============================================================================

#' Ridge-penalized Cox prognostic model (compute only)
#'
#' @param x numeric matrix (samples x genes); colnames are gene symbols.
#' @param y survival data.frame aligned to \code{x} rows.
#' @param time,status column names in \code{y} (defaults "time", "status");
#'   \code{status} must be 1 = event, 0 = censored.
#' @param nfolds number of cross-validation folds (default 10).
#' @param seed integer seed set before cross-validation for a reproducible fold
#'   split and lambda.min (default 1).
#' @return a list with the full coefficient table (\code{gene}, \code{coef}; all
#'   genes, since ridge does not zero out coefficients), \code{lambda_min},
#'   \code{n_features}, and the \code{cv.glmnet} object.
signature_ridge_cox <- function(x, y, time = "time", status = "status",
```

```
                                                  nfolds = 10, seed = 1) {
  x <- as.matrix(x)
  if (!all(c(time, status) %in% names(y)))
    stop("`y` must contain columns: ", time, ", ", status)
  if (nrow(x) != nrow(y))
    stop("nrow(x) must equal nrow(y) (samples aligned row-wise).")
  if (is.null(colnames(x))) colnames(x) <- paste0("gene", seq_len(ncol(x)))

  tt <- as.numeric(y[[time]])
  st <- as.integer(y[[status]])
  ok <- is.finite(tt) & tt > 0 & st %in% c(0L, 1L) & stats::complete.cases(x)
  x   <- x[ok, , drop = FALSE]; tt <- tt[ok]; st <- st[ok]
  if (nrow(x) < 2L * nfolds)
    stop("too few complete samples (", nrow(x), ") for ", nfolds, "-fold CV.")

  surv <- survival::Surv(tt, st)
  set.seed(seed)
  cvfit <- glmnet::cv.glmnet(x, surv, family = "cox", alpha = 0, nfolds = nfolds)

  cf <- as.matrix(stats::coef(cvfit, s = "lambda.min"))
  cdf <- data.frame(gene = rownames(cf), coef = as.numeric(cf[, 1]),
                    row.names = NULL)
  cdf <- cdf[order(-abs(cdf$coef)), , drop = FALSE]
  rownames(cdf) <- NULL

  list(coef = cdf, lambda_min = cvfit$lambda.min,
       n_features = nrow(cdf), cvfit = cvfit)
}

# ---- Example (simulated data; select this block and run) --------------------
if (FALSE) {
  set.seed(1)
  n <- 160; p <- 30
  x <- matrix(rnorm(n * p), n, p, dimnames = list(NULL, paste0("Gene", 1:p)))
  beta <- c(rep(0.5, 8), rep(0, p - 8))                 # several weak contributors
  risk <- as.numeric(x %*% beta)
  time    <- rexp(n, rate = 0.1 * exp(risk))
  status <- rbinom(n, 1, 0.7)
  y <- data.frame(time = time, status = status)
  res <- signature_ridge_cox(x, y)
  head(res$coef); res$lambda_min
}
# =============================================================================
# signature_rsf()  —  Random survival forest variable importance
#
# Method : Random survival forest (randomForestSRC::rfsrc) grown on
#          Surv(time, status) ~ all features. Each tree splits on the log-rank
#          criterion; the ensemble yields an out-of-bag prediction error (based
#          on Harrell's C-index) and, with permutation importance enabled, a
#          variable importance (VIMP) score per feature. Ranking features by VIMP
#          gives a non-parametric, interaction-aware alternative to penalized Cox
#          for prioritizing prognostic genes.
# Input   : df   data.frame with columns time (> 0), status (1 = event,
#                0 = censored), and one column per candidate feature (gene).
# Output : list(vimp = data.frame(variable, vimp) ordered by vimp desc,
#          err_rate = final out-of-bag error, ntree). Compute only.
# Depends: randomForestSRC.
```

```r
# ==============================================================================

#' Random survival forest variable importance (compute only)
#'
#' @param df data.frame with the time, status and feature columns.
#' @param time,status column names in \code{df} (defaults "time", "status");
#'     \code{status} must be 1 = event, 0 = censored.
#' @param ntree number of trees in the forest (default 500).
#' @param seed integer; passed to \code{rfsrc} as its native negative-integer
#'     seed (\code{-abs(seed)}) for reproducibility (default 1).
#' @return a list with the VIMP table (\code{variable}, \code{vimp}; permutation
#'     importance, higher = more important), the final out-of-bag \code{err_rate}
#'     (1 - C-index), and \code{ntree}.
signature_rsf <- function(df, time = "time", status = "status",
                          ntree = 500, seed = 1) {
  if (!requireNamespace("randomForestSRC", quietly = TRUE))
    stop("package 'randomForestSRC' is required.")
  if (!all(c(time, status) %in% names(df)))
    stop("`df` must contain columns: ", time, ", ", status)
  feats <- setdiff(names(df), c(time, status))
  if (length(feats) < 2L)
    stop("need >= 2 feature columns besides time/status.")

  d <- df[stats::complete.cases(df[, c(time, status, feats)]), , drop = FALSE]
  d <- d[as.numeric(d[[time]]) > 0, , drop = FALSE]
  if (nrow(d) < 10L) stop("need >= 10 complete samples with positive time.")

  # Standardized frame so the formula's Surv(time, status) columns are present.
  work <- d[, feats, drop = FALSE]
  work$time   <- as.numeric(d[[time]])
  work$status <- as.integer(d[[status]])

  # rfsrc parses the Surv(...) term from the formula string (it does not
  # evaluate it), so a bare Surv() is required here and survival need not be
  # attached.
  fit <- randomForestSRC::rfsrc(
    Surv(time, status) ~ ., data = work,
    ntree = ntree, importance = "permute", seed = -abs(as.integer(seed)))

  vimp <- fit$importance
  vdf  <- data.frame(variable = names(vimp), vimp = as.numeric(vimp),
                     row.names = NULL)
  vdf  <- vdf[order(-vdf$vimp), , drop = FALSE]
  rownames(vdf) <- NULL

  err <- fit$err.rate
  err <- err[length(err)]                      # final cumulative OOB error

  list(vimp = vdf, err_rate = as.numeric(err), ntree = fit$ntree)
}

# ---- Example (simulated data; select this block and run) --------------------
if (FALSE) {
  set.seed(1)
  n <- 200; p <- 15
  X <- matrix(rnorm(n * p), n, p, dimnames = list(NULL, paste0("Gene", 1:p)))
  beta <- c(rep(1.0, 3), rep(0, p - 3))        # first 3 genes prognostic
```

```r
  risk <- as.numeric(X %*% beta)
  time    <- rexp(n, rate = 0.1 * exp(risk))
  status <- rbinom(n, 1, 0.7)
  df <- data.frame(time = time, status = status, X)
  res <- signature_rsf(df, ntree = 300)
  head(res$vimp); res$err_rate
}
# ==============================================================================
# signature_stepcox()  —  Stepwise Cox model selection by AIC
#
# Method : Stepwise variable selection for a Cox proportional-hazards model.
#          Starting from the full model Surv(time, status) ~ all features,
#          MASS::stepAIC adds and/or drops terms to minimize the Akaike
#          information criterion. The direction ("both", "backward", "forward")
#          controls whether terms may only be dropped, only added, or both. The
#          retained terms and their Cox coefficients form the signature.
# Input  : df   data.frame with columns time (> 0), status (1 = event,
#               0 = censored), and one column per candidate feature (gene).
# Output : list(coef = data.frame(feature, coef, HR, se, z, p.value) for the
#          selected terms, n_selected, aic, formula). Compute only.
# Depends: survival, MASS.
# ==============================================================================

#' Stepwise Cox model selection by AIC (compute only)
#'
#' @param df data.frame with the time, status and feature columns.
#' @param time,status column names in \code{df} (defaults "time", "status");
#'    \code{status} must be 1 = event, 0 = censored.
#' @param direction stepwise search direction passed to
#'    \code{\link[MASS]{stepAIC}}: "both" (default), "backward" or "forward".
#' @param trace if TRUE, print the stepAIC search trace (default FALSE).
#' @return a list with the selected-term coefficient table, the number of
#'    selected terms, the final model AIC, and the selected model formula.
signature_stepcox <- function(df, time = "time", status = "status",
                              direction = c("both", "backward", "forward"),
                              trace = FALSE) {
  direction <- match.arg(direction)
  if (!all(c(time, status) %in% names(df)))
    stop("`df` must contain columns: ", time, ", ", status)
  feats <- setdiff(names(df), c(time, status))
  if (length(feats) < 2L)
    stop("need >= 2 feature columns besides time/status.")

  d <- df[stats::complete.cases(df[, c(time, status, feats)]), , drop = FALSE]
  d <- d[as.numeric(d[[time]]) > 0, , drop = FALSE]
  if (nrow(d) < length(feats) + 2L)
    stop("too few complete samples (", nrow(d), ") for ", length(feats),
         " features.")

  work <- d[, feats, drop = FALSE]
  work$.surv <- survival::Surv(as.numeric(d[[time]]), as.integer(d[[status]]))
  full <- survival::coxph(.surv ~ ., data = work)

  step <- MASS::stepAIC(full, direction = direction, trace = trace)
  cf    <- summary(step)$coefficients
  if (is.null(cf) || nrow(cf) == 0L)
    return(list(coef = data.frame(feature = character(0), coef = numeric(0),
```

```
                                   HR = numeric(0), se = numeric(0),
                                   z = numeric(0), p.value = numeric(0)),
                  n_selected = 0L, aic = stats::AIC(step),
                  formula = deparse(stats::formula(step))))

  tab <- data.frame(feature = rownames(cf),
                      coef       = cf[, "coef"],
                      HR          = cf[, "exp(coef)"],
                      se          = cf[, "se(coef)"],
                      z            = cf[, "z"],
                      p.value = cf[, "Pr(>|z|)"],
                      row.names = NULL)
  list(coef = tab, n_selected = nrow(tab), aic = stats::AIC(step),
       formula = deparse(stats::formula(step)))
}

# ---- Example (simulated data; select this block and run) --------------------
if (FALSE) {
  set.seed(1)
  n <- 200; p <- 12
  X <- matrix(rnorm(n * p), n, p, dimnames = list(NULL, paste0("Gene", 1:p)))
  beta <- c(rep(0.9, 3), rep(0, p - 3))                    # first 3 genes prognostic
  risk <- as.numeric(X %*% beta)
  time     <- rexp(n, rate = 0.1 * exp(risk))
  status <- rbinom(n, 1, 0.7)
  df <- data.frame(time = time, status = status, X)
  res <- signature_stepcox(df, direction = "both")
  res$coef; res$formula
}
# =============================================================================
# signature_survivalroc()  —  Time-dependent ROC of a risk score (survivalROC)
#
# Method : Time-dependent ROC curve for a continuous marker (e.g. a prognostic
#              risk score) at a single landmark time, using survivalROC::survivalROC.
#              Sensitivity/specificity are computed over marker cutoffs with the
#              censoring-adjusted Kaplan-Meier method ("KM"); the nearest-neighbour
#              estimator ("NNE") is also available and then needs a smoothing span.
#              The area under the curve summarizes discrimination at that time.
# Input   : df                  data.frame with columns time (follow-up), status
#                                  (1 = event, 0 = censored) and a continuous marker column.
#              predict_time   landmark time at which to build the ROC curve.
# Output : list(AUC, roc_df = data.frame(cutoff, TP, FP), predict_time, method).
#              Compute only  —  no plotting (roc_df is returned for the caller to plot).
# Depends: survivalROC.
# =============================================================================

#' Time-dependent ROC of a risk score at one landmark time (compute only)
#'
#' @param df data.frame with the time, status and marker columns.
#' @param time,status,marker column names in \code{df} (defaults "time",
#'    "status", "risk"). \code{status} must be 1 = event, 0 = censored.
#' @param predict_time landmark time at which the ROC curve is built; defaults to
#'    the median follow-up time.
#' @param method "KM" (default) or "NNE" passed to
#'    \code{\link[survivalROC]{survivalROC}}.
#' @param span smoothing span for method "NNE"; if NULL a data-driven default of
#'    \code{0.25 * n^(-0.2)} is used. Ignored for method "KM".
```

```r
#' @return a list with the \code{AUC}, an ROC \code{data.frame} (cutoff, TP, FP),
#'     the \code{predict_time} and the \code{method}.
signature_survivalroc <- function(df, time = "time", status = "status",
                                  marker = "risk", predict_time = NULL,
                                  method = c("KM", "NNE"), span = NULL) {
  if (!requireNamespace("survivalROC", quietly = TRUE))
    stop("package 'survivalROC' is required.")
  method <- match.arg(method)
  for (col in c(time, status, marker))
    if (!col %in% names(df)) stop("column not found: ", col)

  d <- data.frame(time    = as.numeric(df[[time]]),
                  status = as.integer(df[[status]]),
                  marker = as.numeric(df[[marker]]))
  d <- d[stats::complete.cases(d) & d$time > 0, , drop = FALSE]
  if (nrow(d) < 10L) stop("need >= 10 complete samples with positive time.")

  if (is.null(predict_time)) predict_time <- stats::median(d$time)
  if (method == "NNE" && is.null(span)) span <- 0.25 * nrow(d)^(-0.20)

  roc <- survivalROC::survivalROC(Stime = d$time, status = d$status,
                                  marker = d$marker,
                                  predict.time = predict_time,
                                  method = method, span = span)

  roc_df <- data.frame(cutoff = as.numeric(roc$cut.values),
                       TP = as.numeric(roc$TP), FP = as.numeric(roc$FP),
                       row.names = NULL)
  list(AUC = as.numeric(roc$AUC), roc_df = roc_df,
       predict_time = predict_time, method = method)
}

# ---- Example (simulated data; select this block and run) --------------------
if (FALSE) {
  set.seed(1)
  n <- 300
  risk    <- rnorm(n)
  time    <- rexp(n, rate = 0.1 * exp(0.9 * risk))        # higher risk, shorter time
  status <- rbinom(n, 1, 0.7)
  df <- data.frame(time = time, status = status, risk = risk)
  res <- signature_survivalroc(df, predict_time = 5, method = "KM")
  res$AUC; head(res$roc_df)
}
# ==============================================================================
# signature_timeroc()  —  Time-dependent AUC of a risk score (timeROC)
#
# Method : Time-dependent ROC analysis for a continuous marker (e.g. a prognostic
#          risk score) under right censoring, using timeROC::timeROC with the
#          incident/dynamic (cumulative-case) definition and inverse-probability-
#          of-censoring weighting. The area under the ROC curve (AUC) is computed
#          at each requested landmark time, quantifying how well the score
#          discriminates subjects who have the event by that time from those who
#          have not.
# Input  : df      data.frame with columns time (follow-up), status (1 = event,
#                  0 = censored) and a continuous marker column.
#          times   numeric vector of landmark times at which to evaluate AUC.
# Output : data.frame(time, AUC)  —  one row per landmark time. Compute only.
```

```
# Depends: timeROC (which in turn needs the survival package on the search path;
#          library(timeROC) attaches it automatically).
# ============================================================================

#' Time-dependent AUC of a risk score (compute only)
#'
#' @param df data.frame with the time, status and marker columns.
#' @param time,status,marker column names in \code{df} (defaults "time",
#'   "status", "risk"). \code{status} must be 1 = event, 0 = censored.
#' @param times numeric vector of landmark times; defaults to the 25/50/75th
#'   percentiles of the observed follow-up times.
#' @param cause the event value in \code{status} treated as the event of
#'   interest (default 1).
#' @return data.frame with columns \code{time} and \code{AUC}.
signature_timeroc <- function(df, time = "time", status = "status",
                              marker = "risk", times = NULL, cause = 1) {
  if (!requireNamespace("timeROC", quietly = TRUE))
    stop("package 'timeROC' is required.")
  for (col in c(time, status, marker))
    if (!col %in% names(df)) stop("column not found: ", col)

  d <- data.frame(time   = as.numeric(df[[time]]),
                  status = as.integer(df[[status]]),
                  marker = as.numeric(df[[marker]]))
  d <- d[stats::complete.cases(d) & d$time > 0, , drop = FALSE]
  if (nrow(d) < 10L) stop("need >= 10 complete samples with positive time.")

  if (is.null(times))
    times <- as.numeric(stats::quantile(d$time, probs = c(0.25, 0.5, 0.75)))
  times <- sort(unique(times[is.finite(times) & times > 0]))
  if (length(times) == 0L) stop("no valid landmark `times`.")

  roc <- timeROC::timeROC(T = d$time, delta = d$status, marker = d$marker,
                          cause = cause, times = times, iid = FALSE)

  data.frame(time = as.numeric(roc$times), AUC = as.numeric(roc$AUC),
             row.names = NULL)
}

# ---- Example (simulated data; select this block and run) -------------------
if (FALSE) {
  library(survival)                    # timeROC's IPC weighting calls Surv()
  set.seed(1)
  n <- 300
  risk   <- rnorm(n)
  time   <- rexp(n, rate = 0.1 * exp(0.9 * risk))      # higher risk, shorter time
  status <- rbinom(n, 1, 0.7)
  df <- data.frame(time = time, status = status, risk = risk)
  res <- signature_timeroc(df, times = c(2, 5, 8))
  res
}
# ============================================================================
# cmap_xsum() — Connectivity Map eXtreme Sum (XSum) score
#
# Method : XSum connectivity (Cheng et al., 2014). A query signature is split
#          into up- and down-regulated gene sets. Each reference profile
#          (perturbation) is reduced to its "extreme" set — the topN highest and
```

```
#                   topN lowest genes by profile value, all other genes zeroed. The score
#                   is    sum(reference values of query-up genes in the extreme set)
#                   minus sum(reference values of query-down genes in the extreme set).
#                   A strongly negative XSum means the perturbation reverses the query
#                   signature (a candidate for repurposing); positive means it mimics it.
# Input    : up, down    character vectors of query up/down gene symbols.
#                   reference genes x perturbations numeric matrix (rownames = gene
#                                   symbols); values are the reference expression / statistic.
# Output : data.frame(perturbation, xsum) ordered by xsum (most negative =
#                   strongest reversal first). Compute only  —  no plotting.
# Depends: base R only.
# ==============================================================================

#' Connectivity Map XSum (eXtreme Sum) connectivity score
#'
#' @param up character vector of query up-regulated gene symbols.
#' @param down character vector of query down-regulated gene symbols.
#' @param reference genes x perturbations numeric matrix; rownames are gene
#'      symbols, one column per perturbation (drug / knockdown / etc.).
#' @param topN size of each extreme tail (highest and lowest genes) kept per
#'      reference profile (default 500); capped at \code{floor(nrow/2)}.
#' @return data.frame(perturbation, xsum), ordered by \code{xsum} ascending.
cmap_xsum <- function(up, down, reference, topN = 500) {
  reference <- as.matrix(reference)
  if (is.null(rownames(reference)))
    stop("`reference` must have gene symbols as rownames.")
  if (is.null(colnames(reference)))
    colnames(reference) <- paste0("perturbation", seq_len(ncol(reference)))

  genes <- rownames(reference)
  n         <- nrow(reference)
  topN    <- min(topN, floor(n / 2))
  if (topN < 1L) stop("`reference` has too few genes for topN >= 1.")

  up      <- intersect(up,      genes)
  down <- intersect(down, genes)
  if (!length(up) && !length(down))
    stop("neither `up` nor `down` genes are present in `reference`.")

  xsum <- vapply(seq_len(ncol(reference)), function(j) {
    r       <- reference[, j]
    ord    <- order(r)                                        # ascending by profile value
    keep <- numeric(n); names(keep) <- genes           # non-extreme genes stay 0
    lo      <- ord[seq_len(topN)]                             # most-negative tail
    hi      <- ord[seq.int(n - topN + 1L, n)]               # most-positive tail
    keep[lo] <- r[lo]
    keep[hi] <- r[hi]
    sum(keep[up]) - sum(keep[down])
  }, numeric(1))

  out <- data.frame(perturbation = colnames(reference), xsum = xsum,
                                row.names = NULL)
  out <- out[order(out$xsum), ]                               # strongest reversal on top
  rownames(out) <- NULL
  out
}
```

```r
# ---- Example (simulated data; select this block and run) --------------------
if (FALSE) {
  set.seed(1)
  genes <- paste0("Gene", 1:2000)
  reference <- matrix(rnorm(2000 * 10), nrow = 2000,
                      dimnames = list(genes, paste0("P", 1:10)))
  up   <- paste0("Gene", 1:50)
  down <- paste0("Gene", 51:100)
  # P1 reverses the query: query-up genes pushed low, query-down genes high.
  reference[up,   "P1"] <- reference[up,   "P1"] - 5
  reference[down, "P1"] <- reference[down, "P1"] + 5
  res <- cmap_xsum(up, down, reference, topN = 200)
  head(res)          # P1 carries the most negative XSum (strongest reversal)
}
# ==============================================================================
# drug_response_correlation() — Gene expression vs drug sensitivity correlation
#
# Method : Correlate the expression of one or more genes against per-sample drug
#          sensitivity (e.g. IC50 or AUC) with stats::cor.test (Spearman by
#          default): one test per gene x drug pair, on the samples where both
#          values are finite, then Benjamini-Hochberg across all tests. Supply
#          your own drug-sensitivity values — no reference database is embedded.
# Input  : expr   numeric matrix, genes in rows (rownames = gene symbols),
#                 samples in columns.
#          drug_sensitivity   either a named numeric vector (one drug; names =
#                 sample IDs) or a samples x drugs numeric matrix / data.frame
#                 (rownames = sample IDs, one column per drug).
#          genes character vector of gene symbols (rows of `expr`) to test.
# Output : data.frame(gene, drug, rho, p.value, p.adj, n) ordered by p.value.
#          Compute only — no plotting.
# Depends: base R only (Hmisc::rcorr is a faster alternative for large panels).
# ==============================================================================

#' Correlate gene expression against drug sensitivity
#'
#' @param expr numeric matrix (genes x samples); rownames are gene symbols,
#'   columns are samples.
#' @param drug_sensitivity per-sample drug sensitivity: a named numeric vector
#'   (single drug; names = sample IDs) or a samples x drugs numeric matrix /
#'   data.frame (rownames = sample IDs, one column per drug).
#' @param genes character vector of gene symbols to test (rows of \code{expr}).
#' @param method correlation method, "spearman" (default) or "pearson".
#' @param p_adjust multiple-testing method for \code{\link[stats]{p.adjust}}
#'   (default "BH"), applied across all gene x drug tests.
#' @param min_pairs minimum complete (both-finite) sample pairs per test
#'   (default 3).
#' @return data.frame, one row per gene x drug pair, ordered by \code{p.value}.
drug_response_correlation <- function(expr, drug_sensitivity, genes,
                                      method = c("spearman", "pearson"),
                                      p_adjust = "BH", min_pairs = 3) {
  method <- match.arg(method)
  expr   <- as.matrix(expr)
  if (is.null(colnames(expr)))
    stop("`expr` must have sample IDs as column names.")

  # Normalise drug sensitivity to a samples x drugs matrix.
  if (is.null(dim(drug_sensitivity))) {
```

```r
    if (is.null(names(drug_sensitivity)))
      stop("a vector `drug_sensitivity` must be named by sample ID.")
    ds <- matrix(drug_sensitivity, ncol = 1,
                 dimnames = list(names(drug_sensitivity), "drug"))
  } else {
    ds <- as.matrix(drug_sensitivity)
    if (is.null(rownames(ds)))
      stop("a matrix `drug_sensitivity` must have sample IDs as rownames.")
    if (is.null(colnames(ds)))
      colnames(ds) <- paste0("drug", seq_len(ncol(ds)))
  }

  genes <- intersect(genes, rownames(expr))
  if (!length(genes)) stop("none of `genes` are rows of `expr`.")
  common <- intersect(colnames(expr), rownames(ds))
  if (length(common) < min_pairs)
    stop("only ", length(common), " shared samples; need >= ", min_pairs, ".")
  expr  <- expr[genes, common, drop = FALSE]
  ds    <- ds[common, , drop = FALSE]
  drugs <- colnames(ds)

  grid <- expand.grid(gene = genes, drug = drugs, stringsAsFactors = FALSE)
  stat <- t(vapply(seq_len(nrow(grid)), function(i) {
    x  <- as.numeric(expr[grid$gene[i], ])
    y  <- as.numeric(ds[, grid$drug[i]])
    ok <- is.finite(x) & is.finite(y)
    n  <- sum(ok)
    if (n < min_pairs) return(c(rho = NA_real_, p = NA_real_, n = n))
    ht <- suppressWarnings(stats::cor.test(x[ok], y[ok], method = method))
    c(rho = unname(ht$estimate), p = ht$p.value, n = n)
  }, numeric(3)))

  out <- data.frame(gene = grid$gene, drug = grid$drug,
                    rho = stat[, "rho"], p.value = stat[, "p"],
                    p.adj = stats::p.adjust(stat[, "p"], method = p_adjust),
                    n = stat[, "n"], row.names = NULL, check.names = FALSE)
  out <- out[order(out$p.value), ]
  rownames(out) <- NULL
  out
}

# ---- Example (simulated data; select this block and run) --------------------
if (FALSE) {
  set.seed(1)
  samples <- paste0("S", 1:40)
  expr <- matrix(rnorm(100 * 40), nrow = 100,
                 dimnames = list(paste0("Gene", 1:100), samples))
  # Two drugs; sensitivity to Drug A tracks Gene1 expression.
  drugA <- 0.8 * expr["Gene1", ] + rnorm(40, sd = 0.5)
  drugB <- rnorm(40)
  ds <- cbind(DrugA = drugA, DrugB = drugB)          # samples x drugs
  res <- drug_response_correlation(expr, ds, genes = c("Gene1", "Gene2"),
                                   method = "spearman")
  head(res)
}
# =============================================================================
# drug_response_oncopredict()  —  Impute drug response with oncoPredict
```

```
#
# Method : Ridge-regression imputation of drug sensitivity (Geeleher et al.'s
#          pRRophetic approach, as reimplemented in oncoPredict::calcPhenotype).
#          A model is trained on a reference panel of cell lines / samples with
#          known drug response and applied to a test expression matrix to predict
#          per-sample sensitivity for every drug. All reference matrices are
#          supplied by the caller – nothing is embedded.
# Input  : training_expr    genes x samples numeric matrix (reference profiles).
#          training_ptype   reference drug response: a named numeric vector (one
#                           drug) or a samples x drugs numeric matrix, sample
#                           order matching columns of training_expr.
#          test_expr        genes x samples numeric matrix to predict for.
# Output : test-samples x drugs numeric matrix of imputed sensitivity, read back
#          from the DrugPredictions.csv that calcPhenotype writes.
# Depends: oncoPredict (Bioconductor / GitHub).
# ============================================================================

#' Impute drug response with oncoPredict::calcPhenotype
#'
#' @param training_expr genes x samples numeric matrix of reference expression.
#' @param training_ptype reference drug response: a named numeric vector (single
#'   drug) or a samples x drugs numeric matrix aligned to \code{training_expr}.
#' @param test_expr genes x samples numeric matrix to impute response for.
#' @param output_dir directory to run calcPhenotype in; it writes
#'   \code{calcPhenotype_Output/DrugPredictions.csv} there (default a tempdir).
#' @param batch_correct homogenisation method passed to calcPhenotype (default
#'   "eb"); use "standardize" or "none" for large panels.
#' @param power_transform logical; power-transform the phenotype (default TRUE).
#' @param min_num_samples minimum training samples required (default 10).
#' @param remove_low_varying fraction of lowest-variance genes to drop
#'   (default 0.2).
#' @return a test-samples x drugs numeric matrix of predicted sensitivity.
drug_response_oncopredict <- function(training_expr, training_ptype, test_expr,
                                      output_dir = tempfile("oncopredict_"),
                                      batch_correct = "eb",
                                      power_transform = TRUE,
                                      min_num_samples = 10,
                                      remove_low_varying = 0.2) {
  if (!requireNamespace("oncoPredict", quietly = TRUE))
    stop("Package 'oncoPredict' is required.")
  training_expr <- as.matrix(training_expr)
  test_expr     <- as.matrix(test_expr)
  if (is.null(dim(training_ptype)))
    training_ptype <- matrix(training_ptype, ncol = 1,
                             dimnames = list(names(training_ptype), "drug"))
  training_ptype <- as.matrix(training_ptype)

  # calcPhenotype writes 'calcPhenotype_Output/DrugPredictions.csv' into the
  # working directory, so run it inside `output_dir` and restore the wd after.
  dir.create(output_dir, showWarnings = FALSE, recursive = TRUE)
  old_wd <- getwd(); on.exit(setwd(old_wd), add = TRUE)
  setwd(output_dir)

  oncoPredict::calcPhenotype(
    trainingExprData      = training_expr,
    trainingPtype         = training_ptype,
    testExprData          = test_expr,
```

```r
        batchCorrect                = batch_correct,
        powerTransformPhenotype = power_transform,
        removeLowVaryingGenes    = remove_low_varying,
        minNumSamples                = min_num_samples,
        selection                    = 1,
        printOutput                  = TRUE)

    pred_file <- file.path("calcPhenotype_Output", "DrugPredictions.csv")
    if (!file.exists(pred_file))
        stop("calcPhenotype did not produce ", pred_file, ".")
    pred <- utils::read.csv(pred_file, row.names = 1, check.names = FALSE)
    as.matrix(pred)        # test samples x drugs (imputed sensitivity)
}

# ---- Example (simulated data; select this block and run) --------------------
if (FALSE) {
    library(oncoPredict)
    set.seed(1)
    genes <- paste0("Gene", 1:500)
    train_expr <- matrix(rnorm(500 * 60), nrow = 500,
                                  dimnames = list(genes, paste0("CL", 1:60)))
    # One drug; IC50 depends on Gene1 in the training panel.
    train_ic50 <- 5 - 0.7 * train_expr["Gene1", ] + rnorm(60, sd = 0.3)
    test_expr    <- matrix(rnorm(500 * 20), nrow = 500,
                                  dimnames = list(genes, paste0("T", 1:20)))
    pred <- drug_response_oncopredict(train_expr, train_ic50, test_expr)
    head(pred)        # 20 test samples x 1 drug
}
```
