# Supplementary material for: Prognostic and Immunological Value of CTNNB1 in Pancreatic Adenocarcinoma: A Comprehensive NGS and Multicomponent Analysis
Source: Genet Res (Camb). 2026 Sep 9;2026:8700258. doi: 10.1155/genr/8700258 (PMC13555821; doi:10.1155/genr/8700258)
Supplement: Supplementary file 1 [file GENR-2026-8700258-s001.zip › Supplementary_Figures_Legends.docx]

**Supplementary Figure 1. Independent GEO cohort validation and HPA-antibody-based immunohistochemical characterization of CTNNB1 localization in normal and pancreatic ductal adenocarcinoma tissues.** (A) Validation in three independent GEO cohorts: GSE28735 ( n = 89), GSE62452 ( n = 129), and GSE71729 ( n = 338).(B) Normal pancreatic tissue showing strong and continuous membranous expression of CTNNB1 in exocrine glandular cells (Antibody: HPA029160). (C) PDAC tissue showing prominent cytoplasmic accumulation of CTNNB1 with attenuated membranous staining in tumor cells(Antibody: HPA029160). Scale bar: 200 μm.

**Supplementary Figure 2. Comprehensive analyses of the tumor immune microenvironment correlated with CTNNB1 in TCGA-PAAD cohort.** (A) Correlation analysis of *CTNNB1* expression with tumor-infiltrating immune cell abundance calculated by five independent bioinformatic algorithms (TIMER, EPIC, MCPcounter, CIBERSORT and xCell). Asterisks denote statistical significance. (B) Heatmap showing the correlation between *CTNNB1* expression and four classes of immune-related genes (immunostimulators, chemokines, immunoinhibitors and HLA genes) in PDAC. Red color indicates positive correlation while blue represents negative correlation. *P<0.05, **P<0.01, ***P<0.001. (C) Multi-omics heatmap of immunomodulatory genes across four *CTNNB1* expression quartiles (Q1–Q4, from low to high) in PDAC. Genes were classified into six functional clusters: co-stimulators, co-inhibitors, ligands, receptors, cell adhesion and antigen presentation-related genes. Five parameters including mRNA expression, DNA methylation, copy number amplification frequency, deletion frequency and immune functional annotation (Stimulatory/Inhibitory/N/A) were exhibited. Red denotes upregulation/increase and blue indicates downregulation/decrease, with color bar ranging from −2 to 2. (D) Correlation heatmap between *CTNNB1* expression and PDAC immune and genomic phenotypes, including immune signature score, genomic instability, TME and immunotherapy-related indexes.

**Supplementary Figure 3. Pan-cancer screening and multi-cohort validation of *CTNNB1*-associated candidate therapeutic drugs in pancreatic ductal adenocarcinoma (PDAC).** (A) Ranked pan-cancer correlation analysis between *CTNNB1* expression and drug susceptibility based on four pharmacogenomic databases (PRISM, CTRP, GDSC1, GDSC2); significantly negatively correlated compounds were selected as candidate agents for subsequent validation. (B–E) Spearman’s rank correlation scatter plots validated the negative association between *CTNNB1* expression and drug sensitivity across independent PDAC cohorts: Obidoxime for PRISM (B), MK-0752 for CTRP (C), AZD1332_1463 for GDSC1 (D), Dasatinib_1079 for GDSC2 (E). Analyzed cohorts included TCGA-PDAC (n = 176), GSE21501 (n = 132), GSE62452 (n = 69), GSE78229 (n = 50), GSE79668 (n = 51), and E-MTAB-6134 (n = 308). (F) Connectivity score distribution of Clofibrate from CMap database; a low negative connectivity score indicated its potential to reverse oncogenic transcriptional signatures induced by aberrant *CTNNB1* overexpression in PDAC.
